# A divide-and-conquer phylogenomic approach based on character supermatrices resolves early steps in the evolution of the *Archaea*

**DOI:** 10.1186/s12862-021-01952-0

**Published:** 2022-01-05

**Authors:** Monique Aouad, Jean-Pierre Flandrois, Frédéric Jauffrit, Manolo Gouy, Simonetta Gribaldo, Céline Brochier-Armanet

**Affiliations:** 1 Université de Lyon, Université Lyon 1, CNRS, UMR5558, Laboratoire de Biométrie et Biologie Évolutive, 43 bd du 11 novembre 1918, 69622 Villeurbanne, France; 2 Technology Research Department, Innovation Unit, bioMérieux SA, Marcy Étoile, France; 3 Department of Microbiology, Unit “Evolutionary Biology of the Microbial Cell”, UMR2001, Institut Pasteur, Paris, France; 4 École Supérieure de Biologie-Biochimie-Biotechnologies, Université Catholique de Lyon, 10 place des archives, 69002 Lyon, France

**Keywords:** DPANN, Asgard, *Stygia*, *Diaforarchaea*, *Altiarchaea*, Root

## Abstract

**Background:**

The recent rise in cultivation-independent genome sequencing has provided key material to explore uncharted branches of the Tree of Life. This has been particularly spectacular concerning the *Archaea*, projecting them at the center stage as prominently relevant to understand early stages in evolution and the emergence of fundamental metabolisms as well as the origin of eukaryotes. Yet, resolving deep divergences remains a challenging task due to well-known tree-reconstruction artefacts and biases in extracting robust ancient phylogenetic signal, notably when analyzing data sets including the three Domains of Life. Among the various strategies aimed at mitigating these problems, divide-and-conquer approaches remain poorly explored, and have been primarily based on reconciliation among single gene trees which however notoriously lack ancient phylogenetic signal.

**Results:**

We analyzed sub-sets of full supermatrices covering the whole Tree of Life with specific taxonomic sampling to robustly resolve different parts of the archaeal phylogeny in light of their current diversity. Our results strongly support the existence and early emergence of two main clades, Cluster I and Cluster II, which we name *Ouranosarchaea* and *Gaiarchaea*, and we clarify the placement of important novel archaeal lineages within these two clades. However, the monophyly and branching of the fast evolving nanosized DPANN members remains unclear and worth of further study.

**Conclusions:**

We inferred a well resolved rooted phylogeny of the *Archaea* that includes all recently described phyla of high taxonomic rank. This phylogeny represents a valuable reference to study the evolutionary events associated to the early steps of the diversification of the archaeal domain. Beyond the specifics of archaeal phylogeny, our results demonstrate the power of divide-and-conquer approaches to resolve deep phylogenetic relationships, which should be applied to progressively resolve the entire Tree of Life.

**Supplementary Information:**

The online version contains supplementary material available at 10.1186/s12862-021-01952-0.

## Background

The recent rise in cultivation-independent genome sequencing has provided key material to explore the so-called 'microbial dark matter' and led to the discovery of a myriad of new major bacterial and archaeal lineages with unexpected metabolic and ecological features [1–4]. This has profoundly changed our understanding of microbial biodiversity and challenged established scenarios for the emergence of present-day lineages.

Overlooked for a long time, the *Archaea* have gained a central place in the Tree of Life [5–7]. Not only are they at the origin of important metabolisms such as methanogenesis, but it is now largely accepted that they are at the origin of *Eucarya* [8]. In particular, phylogenetic analyses of recently obtained genomes has identified the Asgard archaea as the closest relatives of *Eucarya*, consistently with their enrichment in Eukaryote Signature Proteins (ESP) [8–11]. However, resolving the deepest divergences in the Tree of Life is a very challenging task. Most recent studies aiming at reconstructing Domain-level phylogenies of the *Archaea* have relied on phylogenetic analyses of large supermatrices of universal genes [1, 6, 10–22]. Supermatrices are very efficient because they combine the weak phylogenetic signal contained in single genes. However, they are also very sensitive to systematic biases, among which the most frequently encountered are those resulting from substitutional biases, multiple substitutions occurring at the same sites, and across-sites evolutionary rate variations [23, 24]. Systematic biases can mask the historical phylogenetic signal and lead to incorrect inferences [25]. The choice of methods specifically designed to overcome these issues, such as the recoding of amino acids, the removal of the fastest-evolving sites and/or species, and the use of sophisticated models of sequence evolution (e.g., site or branch heterogeneous), have disclosed several major tree reconstruction artefacts affecting the deep phylogeny of the archaeal domain. For instance, *Methanopyrus kandleri* has been shown to be a member of the *Methanomada* super-class and not an early diverging lineage [26]. More recently, the position of *Halobacteria* and *Nanohaloarchaeota*, two lineages of extreme halophilic archaea, has been clarified by showing that they emerged independently from two distinct methanogen Class II lineages [27], contradicting the hypothesis that *Nanohaloarchaeota* belong to the DPANN, a proposed deep-branching archaeal superphylum gathering fast-evolving nanosized archaea [1]. Finally, it also revealed that *Methanonatronarchaeia*, a unique archaeal lineage of extremely halophilic, moderately thermophilic, methyl-reducing methanogens, proposed to represent evolutionary intermediates on the path from methanogens to extreme halophiles due to their supposed close relationships with *Halobacteria* [28], branch more deeply in the archaeal phylogeny and independently adapted to these environments [29, 30].

Besides the use of specific methods and models to overcome these systematic biases, divide-and-conquer strategies are also powerful approaches which nevertheless remain poorly explored. They rely on breaking the dataset into smaller subsets, inferring optimal trees for these subsets, and finally combining the resulting trees into a larger tree [24]. Such strategies allow the use of larger taxonomic samplings and more markers, produce higher-quality alignments, and detect more easily tree reconstruction artifacts. For example, such an approach was applied to test the 2Domain versus 3Domain Tree of Life topology by separately analyzing datasets of concatenated protein supermatrices containing *Archaea* and *Eucarya* on one side, and *Archaea* and *Bacteria* on the other [16]. Reconciling the unrooted *Archaea* / *Eucarya* and the rooted *Archaea* / *Bacteria* trees robustly placed *Eucarya* as the sister lineage of the TACK superphylum (no Asgard genomes were available at that time) and disclosed an unexpected root of the archaeal domain [16] that challenges the ‘traditional’ one located between *Euryarchaeota* and the TACK superphylum [22]. Here, the deepest divergence occurred within *Euryarchaeota* and more precisely between two well-supported clades, which were named Cluster I and Cluster II [16]. These results imply a reconsideration of the early evolution of the *Archaea*, and notably all inferences on their last common ancestor.

More recently, Williams and colleagues applied another two-step strategy [31]. First, they inferred a robust unrooted phylogeny of *Archaea* using both supermatrices and supertrees. Then, they applied a probabilistic gene-species tree reconciliation model on 31,236 archaeal gene families that placed the root in-between the DPANN superphylum and a large clade encompassing all other archaea, this clade being further divided into the *Euryarchaeota* on the one hand and the TACK/Asgard groups on the other hand [31]. This approach is interesting because it does not require the use of an outgroup, thus reducing the risk of tree reconstruction artefacts introduced by the long branch of *Bacteria*. Yet, the efficiency of reconciliation approaches relies strongly on the quality of single gene trees, and these often do not contain enough information to obtain firm statistical support for particular nodes, especially the most ancient. Single gene trees are also very sensitive to stochastic errors resulting from the relative short length of the multiple alignments [24] and do not allow to use the most sophisticated evolutionary models which necessitate large multiple alignments. Finally, these approaches rely upon gene-tree rooting techniques for reconciliation with species trees that could be strongly biased [32].

Over the past two years, the tree of *Archaea* has been considerably enriched by the discovery of new major archaeal lineages (e.g. the *Altiarchaea*, the *Stygia*, the *Bathyarchaeota*, the *Theionarchaea*, the *Methanofastidosa*, the *Heimdallarchaeota*, the *Thorarchaeota*, the *Odinarchaeota*, the *Verstraetearchaeota*), some of them representing early-diverging lineages (see [5, 6] and references therein). These taxa are important to understand the earliest steps of the diversification of the *Archaea* and the nature of the last common archaeal ancestor. Here, we evaluated the divide-and-conquer approach based on supermatrices by including these novel data. Our results firmly confirm a specific relationship between *Eucarya* and the Asgard, and strongly support the existence and ancient divergence between Cluster I and Cluster II.

## Results

### A robust unrooted phylogeny of the *Archaea*

**Fig. 1:**
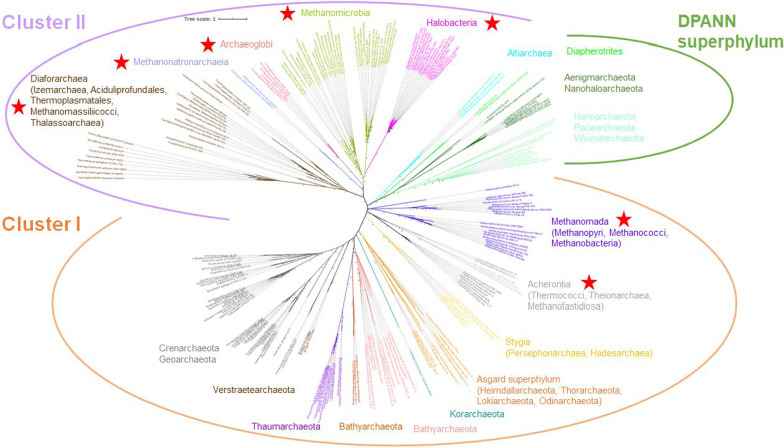
Unrooted Bayesian phylogeny of the *Archaea.* The tree was built using the A supermatrix (72 protein families, 218 taxa, 16,006 amino acids positions). The tree was inferred with PHYLOBAYES using the CAT + GTR + G4 model. The scale bar corresponds to the average number of substitutions per site. Values at branch correspond to posterior probabilities (for clarity, values lower than 0.95 are omitted). Members of *Euryarchaeota* are indicated by red stars to highlight that they do not group together. Cluster I and Cluster II correspond to two major clades as proposed by Raymann et al. (2015). A larger display of the tree is shown in Additional file 1: Fig. S1

We retrieved homologues of the 81 protein families from the study by Raymann et al. (2015) from 435 archaeal proteomes representative of current archaeal diversity. Nine protein families presented complex evolutionary patterns combining multiple horizontal gene transfers (HGT), gene duplications, and gene losses, leading to the non-monophyly of known archaeal orders, and were therefore removed from further analysis. Finally, to avoid taxonomic bias, we selected 218 out of the 435 archaeal genomes by keeping at least three representatives for each archaeal order when possible and only one strain per species. The retaining 72 protein families were concatenated into a supermatrix gathering 16,006 amino acid positions (A supermatrix). The BI and ML analyses of the A supermatrix provided consistent and robust unrooted trees of the archaeal domain (Fig. 1, Additional file 1: Figs. S1 and S2). Notably, they recover a number of clades for which we proposed placeholder names [6, 26]. These names are not meant to identify taxonomic ranks but will help scientific communication about these clades. Remarkably, neither of the two trees recovered the monophyly of *Euryarchaeota*, but instead showed a clear distinction between Cluster I and Cluster II archaea. In fact, *Methanomada* (i.e., *Methanopyri*, *Methanobacteria*, and *Methanococci*) and *Acherontia* (*Thermococci*, *Theionarchaea*, and *Methanofastidosa*) are not monophyletic with other euryarchaeota (i.e., *Diaforarchaea*, *Archaeoglobi*, *Methanonatronarchaeia*, and *Stenosarchaea*) and branch with Cluster I lineages (Fig. 1).

**Fig. 2:**
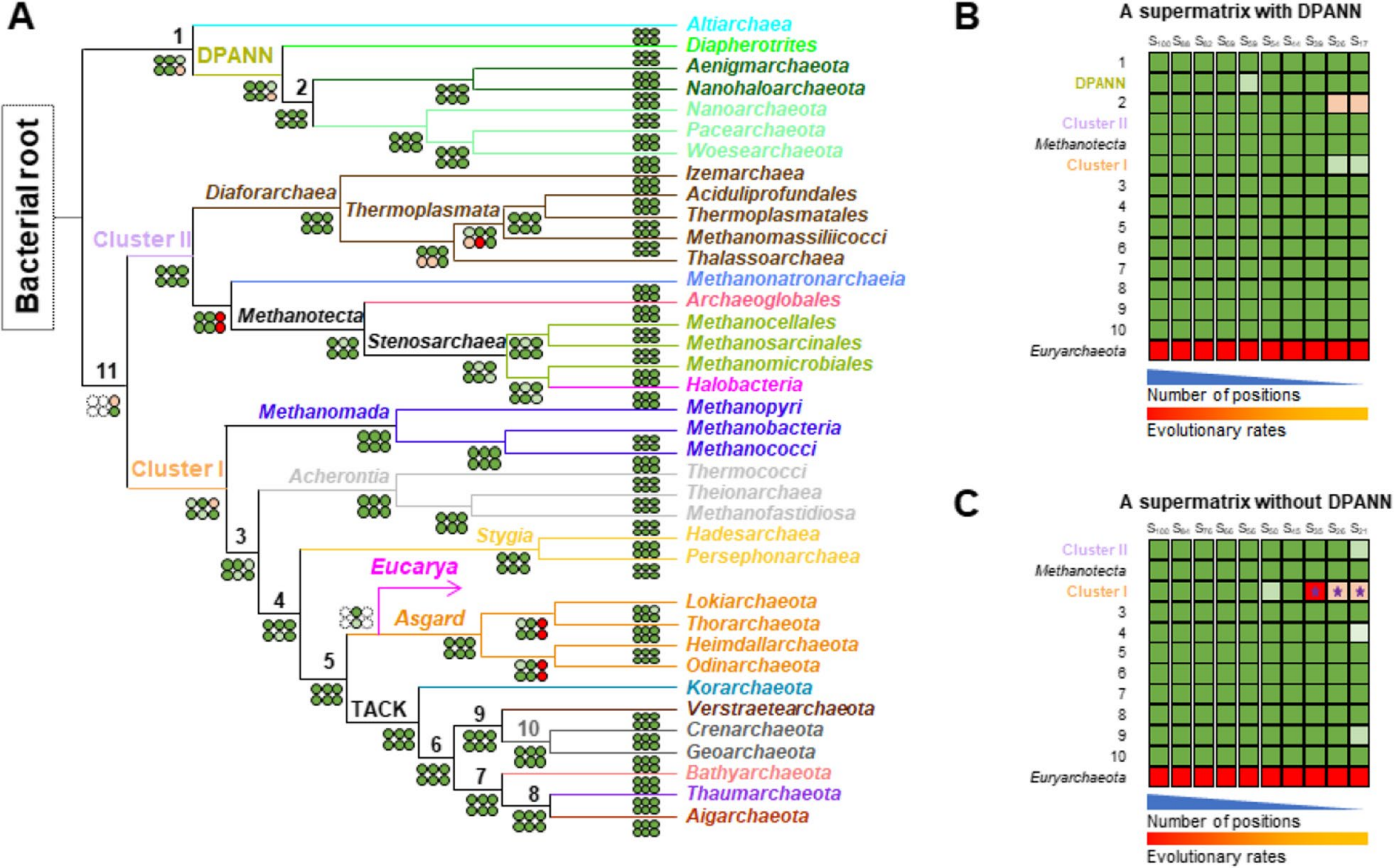
Support of the archaeal topology by the different supermatrices and desaturation analyses. **A** The topology corresponds to the Bayesian tree shown in Fig. 1. Dots correspond to the support of each node by the different analyses. Top dots represent, from left to right, posterior probabilities (PP) of BI trees built with the A, AE, and AB supermatrices, respectively. Bottom dots represent, from left to right, bootstrap values (BV) of ML trees built with the A, AE, and AB supermatrices, respectively. Green signifies branches supported by a PP ≥ 0.95 or a BV ≥ 95%, light green signifies branches supported by a PP < 0.95 or a BV < 95%, light red signifies alternative branching supported by a PP < 0.95 or a BV < 95%, red signifies alternative branching supported by a PP ≥ 0.95 or a BV ≥ 95%. The corresponding ML and BI trees are provided as Additional file 1: Figs. S1, S2, S5, S6, S9, and S10. **B** Results of the slow-fast procedure to the A supermatrix displayed as supports for the main internal branches of the archaeal phylogeny shown in panel (A). The percentage of positions kept for tree inferences is indicated. For instance, S_82_ indicates that 82% of the amino acid positions of the A supermatrix were kept, while the 18% fastest evolving sites were removed. Green squares: branches supported by a PP ≥ 0.95 or a BV ≥ 95%, light green squares: branches supported by a PP < 0.95 or a BV < 95%, light red squares: alternative branching supported by a PP < 0.95 or a BV < 95%, red squares: alternative branching supported by a PP ≥ 0.95 or a BV ≥ 95%. Full trees are provided as Additional file 1: Fig. S3. **C** Identical to B, except that the DPANN sequences were removed from the A supermatrix. Full trees are provided as Additional file 1: Fig. S4

A detailed comparison of supports for these clades and their relationships by the BI and ML analyses and the different supermatrices is presented in Fig. 2A. More precisely, all major lineages (orders, classes, superclasses, phyla, and superphyla) were strongly supported as well as their relationships (most posterior probabilities (PP) = 1 and bootstrap values (BV) > 90%) (green dots in Fig. 2A) in agreement with some recent reports [6, 9, 18]. Regarding Cluster I, both trees support the early emergence of *Korarchaeota* within the TACK superphylum, the clustering of *Crenarchaeota* with *Verstraetarchaeota*, the sisterhood of *Bathyarchaeota* with *Thaumarchaeota* and *Aigarchaeota,* and the branching of *Stygia* in the stem leading to the TACK and Asgard. Concerning the DPANN, both trees inferred a specific relationship with the *Altiarchaea*, as proposed elsewhere [33]. Within Cluster II, both trees strongly support the close relationship between *Halobacteria* and *Methanomicrobiales*, while *Methanocellales* and *Methanosarcinales* formed two sister-lineages in agreement with a recent report [27]. Furthermore, *Methanonatronarchaeia* branched in the stem of *Methanotecta* in agreement with two recent studies showing that *Halobacteria* and *Methanonatronarchaeia* do not represent two sister-lineages [29, 30], as initially proposed [28]. Within DPANN, we have recovered placements consistent with previous works: (i) a basal branching of the *Diapherotrites*, (ii) the sistership of the *Aenigmarchaeota* and the *Nanohaloarchaeota*, and (iii) the grouping of the *Pacearchaeota*, the *Woesearchaeota*, and the *Nanoarchaeota* [31, 33]. The only areas of the archaeal tree that remain unresolved are the internal branching within *Diaforarchaea*, the base of Cluster I, and the relationships within the Asgard (light green and orange dots in Fig. 2A).

We investigated whether the relationships within *Archaea* are affected by tree-reconstruction artefacts by applying the desaturation method to the A supermatrix. The progressive removal of the fastest evolving sites did not reveal major changes in topology or branch support, even when excluding the DPANN (Fig. 2B, C and Additional file 1: Figs. S3 and S4). In particular, the monophyly of *Euryarchaeota* was never observed in these analyses due to the branching of *Altiarchaea* and/or DPANN between Cluster I and Cluster II (Fig. 2B and Additional file 1: Fig. S3) or within Cluster I, as the sister-lineage of *Methanomada* (purple stars, Fig. 2C and Additional file 1: Fig. S4).

### Branching of *Eucarya* with respect to *Archaea*

**Fig. 3:**
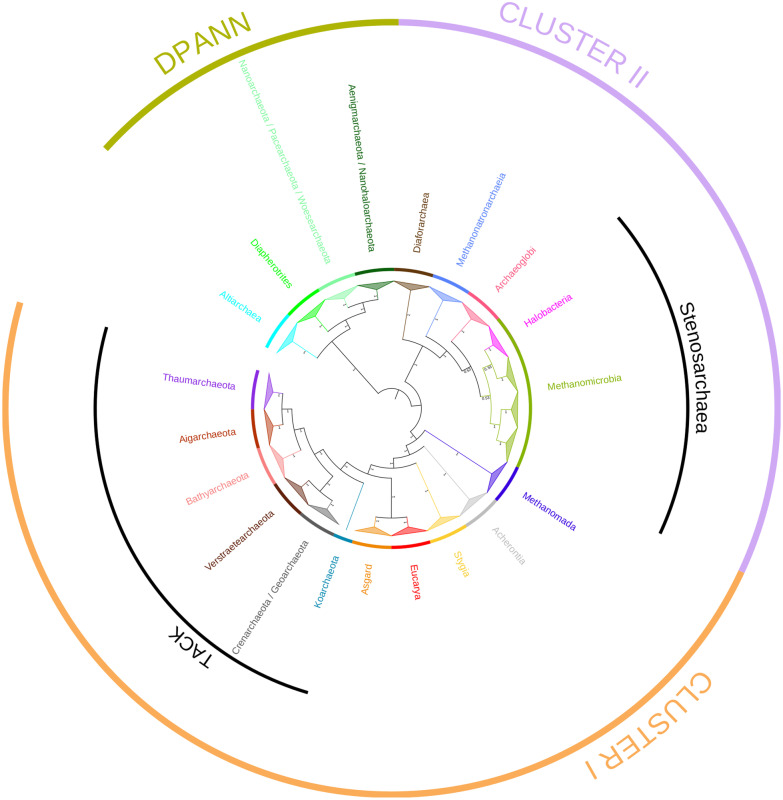
Phylogenetic position of *Eucarya* relative to *Archaea*. Cladogram representation of the unrooted Bayesian tree shown in Additional file 1: Fig. S5. The tree was built using the AE supermatrix (64 protein families, 236 taxa, 13,468 amino acids positions). The tree was inferred with PHYLOBAYES using the CAT + GTR + G4 model. For clarity, the relationships among species within clades have been collapsed. Branch length does not correspond to evolutionary distances. Supports at branches correspond to posterior probabilities

**Fig. 4:**
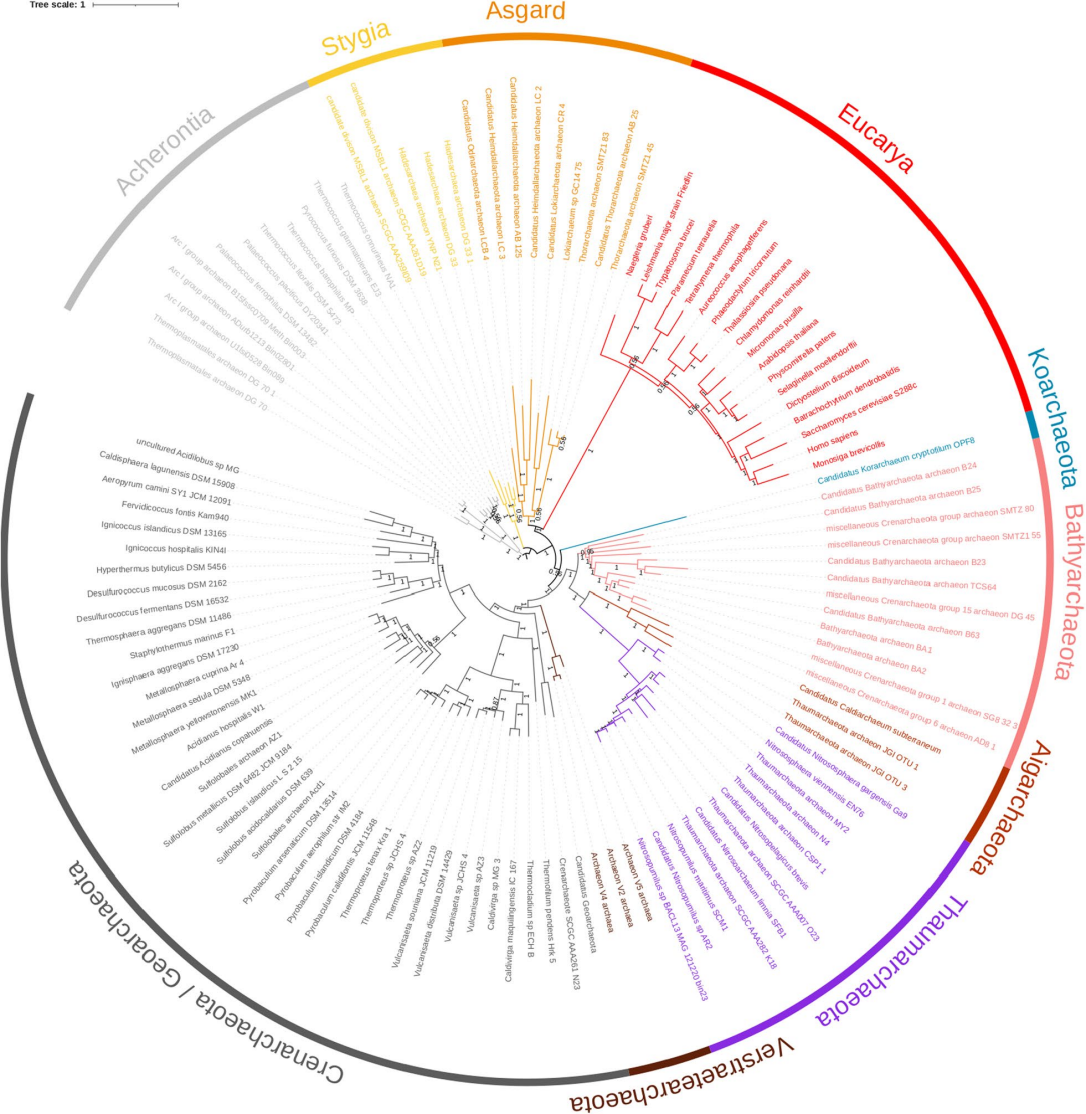
Phylogenetic position of *Eucarya* relative to Cluster I archaea. Unrooted Bayesian phylogenetic tree built using the AE supermatrix (64 protein families, 114 taxa, 13,468 amino acids positions). The tree was inferred with PHYLOBAYES using the CAT + GTR + G4 model. Supports at branches correspond to posterior probabilities

To investigate further the branching of *Eucarya* with respect to *Archaea*, we analyzed a supermatrix containing the 64 protein families shared by *Archaea* and *Eucarya* (AE supermatrix, 13,468 amino acid positions). We obtained consistent and well-resolved unrooted phylogenies (Fig. 3, and Additional file 1: Figs. S5 and S6). The internal archaeal topology previously inferred by the A supermatrix was largely supported by the AE supermatrix (green dots in Fig. 2A), indicating that adding eukaryotes neither introduces tree reconstruction artifacts nor does it blur the phylogenetic signal contained in the data. In both trees the *Eucarya* displayed a very long stem and branched as sister of the Asgard superphylum (PP = 1, BV = 86%, Fig. 3, and Additional file 1: Figs. S5 and S6). To investigate this relationship in more detail and avoid potential artifacts due to the presence of very distantly related lineages or very long branches such as the ones leading to *Halobacteria* or DPANN, we subsampled the original dataset into one including only eukaryotes and their closest archaeal relatives. More precisely, the TACK, the Asgard, the *Eucarya*, and the *Stygia* represented the ingroup, while the *Acherontia* was used of outgroup. The resulting tree is fully resolved and consistently supports the Asgard as the sister-lineage of *Eucarya* (PP = 1, Fig. 4). A sister-grouping of Asgard and *Eucarya* was also observed when applying two different amino acid recoding schemes (here Dayhoff4 and Dayhoff6, Additional file 1: Figs. S7 and S8). These results suggest that the sistership of *Eucarya* and Asgard is robust and confirm their close relationships with TACK and *Stygia* within Cluster I.

### Deep divergences of the *Archaea* inferred by using Bacteria as outgroup

**Fig. 5:**
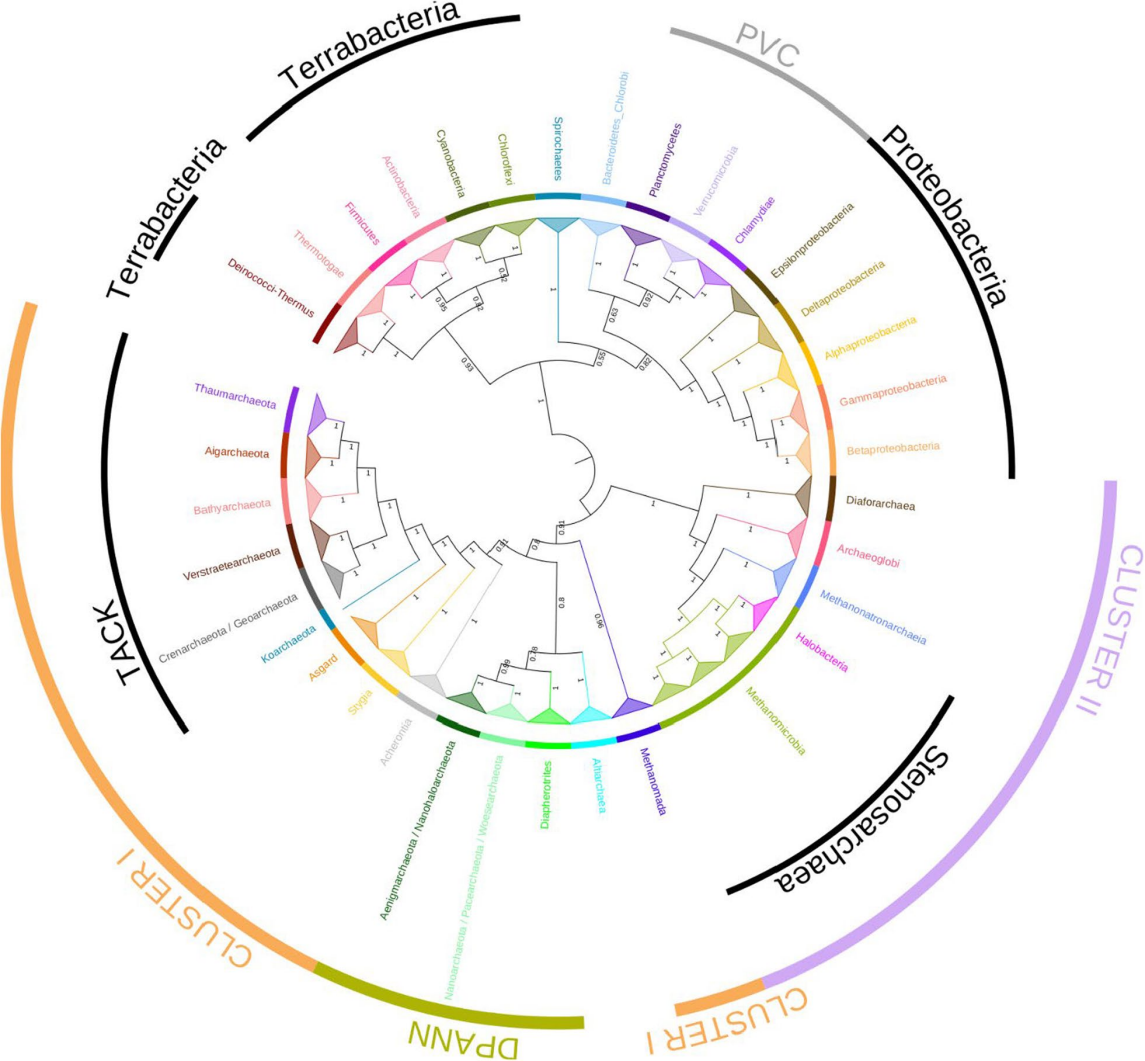
Inference of early divergences in the *Archaea* based on the AB supermatrix. Cladogram representation of the unrooted Bayesian tree shown in Additional file 1: Fig. S9. The tree was built using the AB supermatrix (41 protein families, 285 taxa, 7853 amino acids positions). The tree was inferred with PHYLOBAYES using the CAT + GTR + G4 model. For clarity, the relationships among species within clades have been collapsed. Branch length does not correspond to evolutionary distances. Supports at branches correspond to posterior probabilities

We next sought to investigate the deepest divergences within *Archaea*, notably by resolving the branching of new lineages (Asgard, *Verstraetearchaeota*, *Stygia*, *Methanonatronarchaeia*, and *Altiarchaea*) that were not analyzed in Raymann et al. (2015). We therefore proceeded to root the archaeal tree by using a bacterial outgroup using the 41 protein families shared by *Archaea* and *Bacteria* (AB supermatrix, 7,853 amino acid positions). The inferred ML and BI trees were overall robust and consistent (Fig. 5, Additional file 1: Figs. S9 and S10). In particular, the monophyly of major bacterial groups such as the *Chlorobi* / *Bacteroidetes*, *Spirochaetes*, *Chloroflexi*, *Deinococcus*-*Thermus*, *Cyanobacteria*, *Actinobacteria*, *Firmicutes*, and *Thermotogae*, including *Proteobacteria* and PVC that are particularly difficult to infer, were robustly supported (most PP = 1, BV > 95%). Furthermore, the BI tree recovered the deep split between *Terrabacteria* (*Deinococcus*-*Thermus*, *Chloroflexi*, *Cyanobacteria*, *Actinobacteria*, and *Firmicutes*) and *Thermotogae* on the one hand, and other major bacterial lineages on the other hand (PP = 0.93, Fig. 5 and Additional file 1: Fig. S9), consistently with previous reports [16, 34, 35]. The ML tree is overall consistent with the BI tree, albeit relationships among bacterial lineages are less supported (Additional file 1: Fig. S10). In particular, the position of *Deinococcus*-*Thermus* was unresolved in the ML tree.

Most interestingly, the two trees are again incompatible with the monophyly of *Euryarchaeota*. In fact, both trees supported monophyly of Cluster I (PP = 0.91 and BV = 92%) and Cluster II (PP = 1 and BV = 100%). More precisely, the *Methanomada* represent the deepest branches of the Cluster I (Figs. 2A and 5, and Additional file 1: Figs. S9 and S10), whereas *Acherontia* appear to be the sister-lineage of the large clade encompassing the TACK, the Asgard and the *Stygia* (BI = 1 and BV = 100%, Fig. 5). The speciation between Cluster I and Cluster II occurred deeply in the BI and ML trees of *Archaea*, meaning these two lineages are ancient.

It is worth to notice that the ML and the BI trees are inconsistent regarding the phylogenetic position of DPANN. According to the BI tree, the root is located in-between Cluster I and Cluster II, while the DPANN and *Altiarchaea* branch within Cluster I, albeit with a non-significant support (PP = 0.8, Fig. 5). In contrast, according to the ML tree, the root of *Archaea* is located within the large clade encompassing DPANN and *Altiarchaea*, meaning that this group is paraphyletic (BV = 99%, Additional file 1: Fig. S10). More precisely, the clade grouping *Aenigmarchaeota*, *Nanohaloarchaeota*, *Nanoarchaeota*, *Woesearchaeota*, and *Pacearchaeota* represent the very first diverging lineage, while *Diapherotrites* and *Altiarchaea* emerge later. Even if the relationships among DPANN are not fully resolved, the ML tree suggests that the DPANN superphylum diverged before the speciation between Clusters I and II (BV = 100%). Because BI and trees differ in the placement of the root with respect to DPANN and because the inclusion of the bacterial outgroup impacts the position of DPANN, we wonder whether the DPANN lineages impact the placement of the root itself. To address this question, we removed the DPANN and *Altiarchaea* lineages from the AB supermatrix. The BI tree inferred with this new AB dataset is fully consistent with previous ML and BI trees and supports Cluster I and Cluster II (Additional file 1: Figs. S9-S11). More precisely, the bacterial outgroup branches in-between Cluster I and Cluster II (PP = 0.89), and more importantly, the grouping *Acherontia* with TACK, Asgard, and Stygia taxa is again strongly supported (PP = 1, Additional file 1: Fig. S11).

Based on these rooted archaeal trees, the placement of other recently described lineages can be robustly inferred. For example, the *Verstraetearchaeota*, the Asgard, and the *Stygia* reliably branch with Cluster I lineages (all PP = 1 and BV > 95%), while the *Methanonatronarchaeia* belong to Cluster II (PP = 1 and BV = 100%), in agreement with recent reports [28, 29].

Altogether, these results indicate that the root of *Archaea* can be confidently excluded from within Cluster I or II, although the precise phylogenetic position of DPANN lineages and their relationship with *Altiarchaea* remain to be assessed by future analyses including additional sampling from these lineages.

## Discussion

In recent years, the availability of new genome sequences, especially for archaea and bacteria, along with the development of more realistic phylogenetic models and methods, has provided key material and tools to investigate ancient relationships and evolutionary events. Yet, deep evolutionary relationships within Domains or even the entire Tree of Life have to be robustly assessed by using suitable phylogenetic markers and taxonomic sampling. Here, we have shown that a divide-and-conquer approach based on character supermatrices is a reliable and promising approach to successfully resolve deep divergences by combining the phylogenetic signal brought by large supermatrices and at the same time using different sub-samplings of taxa and markers specifically adapted to different parts of the phylogeny.

Our results provide a robust rooted phylogeny of the *Archaea* allowing to decipher the relative order of divergence of important clades and pinpointing Asgard as the closest relatives of *Eucarya*. The genomic sampling of the Asgard is rapidly increasing, and notably the sequencing of the first Asgard representatives in isolated form [36] will certainly allow to determine more accurately their relationship with eukaryotes. Our data also strongly indicate that *Euryarchaeota* do not form a monophyletic group. Instead, we further support the hypothesis that one of the earliest divergences in the *Archaea* separated two large clades, Cluster I and Cluster II, which we proposed to name *Ouranosarchaea* and *Gaiarchaea*, two ancient and major Greek divinities representing sky and earth, respectively. Again, these names are placeholders and not taxonomic proposals. Because methanogens are present in both clades, whether the root of the *Archaea* lies between Cluster I and II or before their divergence from DPANN is of primary importance to infer whether this metabolism was present in the last archaeal common ancestor. Accordingly, a major open question is whether the monophyly and the basal branching of DPANN are real or rather due to an artifact. While the phylogenetic signal contained in the A and AE supermatrices suggests that DPANN and *Altiarchaea* branch between Cluster I or Cluster II, their phylogenetic position remains unresolved by the AB dataset. This could reflect a lack of phylogenetic signal due the smaller set of markers analyzed and/or to biases resulting from the introduction of the very distant bacterial lineage to root the tree. Investigating the monophyly of the DPANN superphylum, its links with *Altiarchaea*, and its position in relation to Cluster I, Cluster II, remains one of the biggest methodological challenges, which would require the assembly of specific datasets and the implementation of protocols specifically designed to address this issue.

Our analysis significantly increases the ecological and molecular diversity of Cluster I by showing that many recently described lineages belong to this clade. Within Cluster I, the relative divergence order from root to tips is: *Methanomada*, *Acherontia*, *Stygia*, and finally TACK and Asgard. Moreover, we robustly confirm the specific evolutionary relationship between *Eucarya* and Asgard [9–11, 17, 18, 36], although additional genomic sampling of this group will be necessary to resolve more precisely this relationship. We show here that new Cluster I members represent important close outgroups to retrace even further back in time the processes that led to the emergence of eukaryotes from archaeal ancestors.

## Conclusions

The fast pace in acquisition of genomic data from a larger and larger fraction of microbial diversity imposes a giant challenge for obtaining large-scale phylogenies, and hoping to be able to resolve them from tip to toe based on one unique large concatenation [37] does not seem to be a realistic goal. Instead, this prompts for a collective effort to develop new methodological solutions. Here, we propose a divide-and-conquer approach based on the use of character supermatrices instead, to keep higher information, and we apply it to resolve the Domain-level phylogeny of the Archaea, as well as their relationships with eukaryotes. This approach allows the inference of a well resolved rooted phylogeny of the *Archaea* that includes all recently described phyla of high taxonomic rank. This phylogeny represents a valuable reference to study the evolutionary events associated to the early steps of the diversification of the archaeal domain, including the emergence of eukaryotes. Beyond this study, we would like to stress that divide-and-conquer approaches based on supermatrices represent a promising strategy to resolve deep phylogenetic relationships, which should be applied to progressively resolve the entire Tree of Life.

## Methods

### Dataset assembly

435 representative archaeal genomes and their corresponding proteomes were retrieved from the National Center for Biotechnology Information (NCBI) (Additional file 1: Table S1). The 81 protein families from the study by Raymann and colleagues (2015) Additional file 1: Table S2) were updated using the engine of the RiboDB database that combines reciprocal Best-Blast-Hits (rBBH) and Hidden Markov Model (HMM) profiles to identify homologues [38]. More precisely, sequences of the 81 protein families were used as seeds for rBBH to query the 435 proteomes with BLASTP and to build HMM profiles. Subject protein sequences displaying High Scoring Pairs E-values < 10^–4^ or matching with HMM profiles with an E-values < 10^–1^ according to HMMER version 3.1b2 were retained.

The 81 updated protein families were aligned with MAFFT version 7.215 with the high-quality L-INS-I option [39] and trimmed using BMGE version 1.12 with the BLOSUM30 substitution matrix [40]. Trimmed multiple alignments were used to build maximum likelihood trees with IQTREE version 1.6.9 using the best-suited evolutionary model according to ModelFinder (Bayesian information criterion) [41, 42]. Visual inspection of the resulting trees revealed that nine out of the 81 protein families presented complex evolutionary patterns mixing multiple horizontal gene transfers (HGT), gene duplications, and gene losses, leading to known archaeal orders to be non-monophyletic (Additional file 1: Table S2). This is likely due to increased taxonomic sampling which discloses previously undetectable complex evolutionary histories. These nine families were therefore eliminated from further analyses. The remaining 72 families recovered the monophyly of known classes and orders within each of the three domains of life. Among these 72 selected markers, 33 are present in the three domains of life, eight are shared only by *Archaea* and *Bacteria*, and 31 are found only in *Archaea* and *Eucarya*. The phylogeny of these protein families revealed a few punctual HGT or gene duplications. The corresponding sequences were removed from the datasets.

Finally, to avoid taxonomic bias and to reduce computational burden, we selected 218 out of the 435 archaeal genomes by keeping at least three representatives for each archaeal order when possible and only one strain per species (Additional file 1: Tables S1 and S2).

### Supermatrix construction

Three supermatrices were built by combining various subsets of the 72 protein families and taxonomic samplings (Additional file 1: Table S1):

1. The A supermatrix gathered the archaeal sequences from the 72 protein families (218 representative strains including DPANN, 16,006 amino acid positions);
2. The AE supermatrix gathered the eukaryotic and archaeal sequences from 64 protein families: 33 universal families and 31 families shared between *Eucarya* and *Archaea* (236 representative strains including DPANN, 13,468 amino acid positions);
3. The AB supermatrix gathered bacterial and archaeal sequences from 41 protein families: 33 universal families and eight families shared between *Archaea* and *Bacteria* (285 representative strains including DPANN, 7,853 amino acid positions).

For each supermatrix, the single protein families were aligned and trimmed by considering each separate taxonomic sampling in order to limit alignment errors and to maximize the number of positions kept during the trimming step.

### Phylogenetic analysis

Bayesian inference (BI) analyses were performed with PHYLOBAYES version 4.1 [43] with the CAT + GTR + G4 model. The CAT + GTR model allows the amino acid replacement patterns at different sites of a protein alignment to be described by distinct substitution processes [44]. A gamma distribution (G4) with four discrete classes of sites and an estimated alpha parameter was used to model the heterogeneity of evolutionary rates across sites. For some analyses, recoding schemes were applied in order to reduce the substitutional saturation and thus the risk of tree reconstruction artefacts. The Dayhohh4 and Dayhoff6 recoding schemes, corresponding to the four- and six-Dayhoff’s amino acid families were used. They correspond to [(A,G,P, S, T), (D,E,N,Q), (H,K,R), (F,Y,W,I,L,M,V) plus cysteine treated as missing data (C = ?)] and to [(A,G,P, S, T), (D,E,N,Q), (H,K,R), (F,Y,W), (I,L,M,V), (C), respectively.

For tree reconstruction, two chains were run in parallel for at least 10,000 cycles. The first 1,500 trees were discarded as “burnin” and one out of two of the remaining trees from each chain was sampled to test for convergence (maxdiff < 0.3) and to compute 50% majority rule consensus trees.

Maximum likelihood (ML) trees were inferred with IQ-TREE [41], using the LG + C20 + G4 profile mixture model, a ML variant of Bayesian CAT model. Branch supports were computed using the non-parametric procedure implemented in IQ-TREE (100 replicates).

### Desaturation analysis

The progressive site-by-site removal of the fastest-evolving sites was performed using the Slow-Fast method implemented in SLOW-FASTER [45]. At each step, a BI phylogenetic tree was inferred, allowing to follow the impact of the removed positions on important branches [24, 46]. This approach requires the definition of reliable monophyletic groups to estimate the evolutionary rate of each site [46]. Groups corresponding to undisputable monophyletic classes or phyla were considered. For *Archaea*, we defined 19 groups: DPANN group 1 (*Diapherotrites*), DPANN group 2 (*Aenigmarchaeota* and *Nanohaloarchaeota*), DPANN group 3 (*Pacearchaeota*, *Woesearchaeota*, and *Nanoarchaeota*), *Stygia*, *Acherontia*, Crenarchaeota group 1 (*Desulfurococcales*), Crenarchaeota group 2 (*Sulfolobales*), Crenarchaeota group 3 (*Thermoproteales*), Crenarchaeota group 4 (*Geoarchaeota*), *Verstraetearchaeota*, *Thaumarchaeota*, *Aigarchaeota*, *Diaforarchaea*, *Methanomicrobia*, *Methanomada*, *Archaeoglobi*, Asgard, *Bathyarchaeota*, and *Altiarchaea*. *Halobacteria* were not included because their very long stem is a potential source of tree reconstruction artifacts, and they represent a late-emerging lineage whose placement is robustly assessed.

To avoid biases due to unbalanced taxonomic sampling, each group was represented by three-to-seven representatives.

## Supplementary Information

**Additional file 1: Figure S1.** Unrooted Bayesian phylogeny of Archaea. The tree corresponds to the A supermatrix (72 protein families, 218 taxa, 16,006 amino acids positions). The tree was inferred with PHYLOBAYES using the CAT+GTR+G4 model. Values at branch correspond to branch lengths (top) and posterior probabilities (bottom). The scale bar indicates the average number of substitutions per site. **Figure S2.** Unrooted Maximum Likelihood phylogeny of Archaea. The tree corresponds to the A supermatrix (72 protein families (218 taxa, 16,006 amino acids positions). The tree was inferred with IQ-TREE using the LG+C20+G4 model. Values at branch correspond to branch lengths (top) and bootstrap values (bottom). The scale bar indicates the average number of substitutions per site. **Figure S3.** Unrooted Bayesian phylogeny of Archaea inferred through the Slow-Fast procedure. Starting from the A supermatrix (72 protein families, 218 taxa, 16,006 amino acids positions), the fastest evolving sites were removed progressively. At each step, a Bayesian tree was inferred with PHYLOBAYES using the CAT+GTR+G4 model. Values at branch correspond to posterior probabilities. The scale bars indicate the average number of substitutions per site. **Figure S4.** Unrooted Bayesian phylogeny of Archaea inferred through the Slow-Fast procedure by excluding the DPANN. Starting from the A supermatrix (72 protein families, 199 taxa, 15,430 amino acids positions), the fastest evolving sites were removed progressively. At each step, a Bayesian tree was inferred with PHYLOBAYES using the CAT+GTR+G4 model. Values at branch correspond to posterior probabilities. The scale bars indicate the average number of substitutions per site. **Figure S5.** Unrooted Bayesian phylogeny of Archaea and Eucarya. The tree corresponds to the AE supermatrix (61 protein families, 236 taxa, 13,468 amino acids positions). The tree was inferred with PHYLOBAYES using the CAT+GTR+G4 model. Values at branch correspond to branch lengths (top) and posterior probabilities (bottom). The scale bar indicates the average number of substitutions per site. **Figure S6.** Unrooted maximum likelihood phylogeny of Archaea and Eucarya. The tree corresponds to the AE supermatrix (61 protein families, 236 taxa, 13,468 amino acids positions). The tree was inferred with IQ-TREE using the LG+C20+G4 model. Values at branch correspond to branch lengths (top) and bootstrap values (bottom). The scale bar indicates the average number of substitutions per site. **Figure S7.** Phylogenetic position of the Eucarya relatively to Cluster I lineages by Dayhoff4 recoding. Unrooted Bayesian phylogenetic tree built using the AE supermatrix (64 protein families, 114 taxa, 13,468 amino acids positions). The tree was inferred with PHYLOBAYES using the CAT+GTR+G4 model and the amino acid recoding scheme Dayhoff4. The length of the branches corresponds to evolutionary distances. Supports at branch correspond to posterior probabilities. **Figure S8.** Phylogenetic position of the Eucarya relatively to Cluster I lineages by Dayhoff6 recoding. Unrooted Bayesian phylogenetic tree built using the AE supermatrix (64 protein families, 114 taxa, 13,468 amino acids positions). The tree was inferred with PHYLOBAYES using the CAT+GTR+G4 model and the amino acid recoding scheme Dayhoff6. The length of the branches corresponds to evolutionary distances. Supports at branch correspond to posterior probabilities. **Figure S9.** Rooted Bayesian phylogeny of the Archaea. The tree corresponds to the AB supermatrix (41 protein families, 285 taxa, 7,853 amino acids positions). The tree was inferred with PHYLOBAYES using the CAT+GTR+G4 model. Values at branch correspond to branch lengths (top) and posterior probabilities (bottom). The scale bar indicates the average number of substitutions per site. **Figure S10.** Rooted maximum likelihood phylogeny of Archaea. The tree corresponds to the AB supermatrix (41 protein families, 285 taxa, 7,853 amino acids positions). The tree was inferred with IQ-TREE using the LG+C20+G4 model. Values at branch correspond to branch lengths (top) and bootstrap values (bottom). The scale bar indicates the average number of substitutions per site. **Figure S11.** Rooted Bayesian phylogeny of Archaea without DPANN and Altiarchaea. The tree corresponds to a version AB supermatrix built without DPANN and Altiarchaea (41 protein families, 261 taxa, 7,952 amino acids positions). Values at branch correspond to branch lengths (top) and posterior probabilities (bottom). The scale bar indicates the average number of substitutions per site. **Table S1.** List of the taxa used in the study (435 archaea, 67 bacteria, and 18 eukaryotes). The 42 archaeal species useid in the study by Raymann et al. (2015) are indicated. The 218 representative archaeal species kept for phylogenetic analyses are in green. Their presence in the 72 archaeal protein families used in this study is indicated. Representative species kept for the Slow-Fast analysis are in dark green. **Table S2.** List of markers from Raymann et al. (2015) used in this study. For each marker, the corresponding COG name and general description are also indicated. The nine markers displaying complex evolutionary histories are indicated in red. These markers were not retained for supermatrices construction.

## Data Availability

All data generated or analysed during this study are included in this published article and its supplementary information files.

## Abbreviations

ESP: Eukaryote signature proteins
HGT: Horizontal gene transfers
PP: Posterior probabilities
BV: Bootstrap values
AE supermatrix: The supermatrix containing protein families shared by Archaea and Eucarya
AB supermatrix: The supermatrix containing protein families shared by Archaea and Bacteria
NCBI: National Center for Biotechnology Information
rBBH: Reciprocal Best-Blast-Hits
HMM: Hidden Markov Model
BI: Bayesian inference
GA: Gamma distribution
ML: Maximum likelihood
