# Supplementary material for: A divide-and-conquer phylogenomic approach based on character supermatrices resolves early steps in the evolution of the Archaea
Source: BMC Ecol Evol. 2022 Jan 5;22:1. doi: 10.1186/s12862-021-01952-0 (PMC8734073; doi:10.1186/s12862-021-01952-0)
Supplement: Supplementary file 1 — Additional file 1: Figure S1. Unrooted Bayesian phylogeny of Archaea. The tree corresponds to the A supermatrix (72 protein families, 218 taxa, 16,006 amino acids positions). The tree was inferred with PHYLOBAYES using the CAT+GTR+G4 model. Values at branch correspond to branch lengths (top) and posterior probabilities (bottom). The scale bar indicates the average number of substitutions per site. Figure S2. Unrooted Maximum Likelihood phylogeny of Archaea. The tree corresponds to the A supermatrix (72 protein families (218 taxa, 16,006 amino acids positions). The tree was inferred with IQ-TREE using the LG+C20+G4 model. Values at branch correspond to branch lengths (top) and bootstrap values (bottom). The scale bar indicates the average number of substitutions per site. Figure S3. Unrooted Bayesian phylogeny of Archaea inferred through the Slow-Fast procedure. Starting from the A supermatrix (72 protein families, 218 taxa, 16,006 amino acids positions), the fastest evolving sites were removed progressively. At each step, a Bayesian tree was inferred with PHYLOBAYES using the CAT+GTR+G4 model. Values at branch correspond to posterior probabilities. The scale bars indicate the average number of substitutions per site. Figure S4. Unrooted Bayesian phylogeny of Archaea inferred through the Slow-Fast procedure by excluding the DPANN. Starting from the A supermatrix (72 protein families, 199 taxa, 15,430 amino acids positions), the fastest evolving sites were removed progressively. At each step, a Bayesian tree was inferred with PHYLOBAYES using the CAT+GTR+G4 model. Values at branch correspond to posterior probabilities. The scale bars indicate the average number of substitutions per site. Figure S5. Unrooted Bayesian phylogeny of Archaea and Eucarya. The tree corresponds to the AE supermatrix (61 protein families, 236 taxa, 13,468 amino acids positions). The tree was inferred with PHYLOBAYES using the CAT+GTR+G4 model. Values at branch correspond to branch lengths (top [file 12862_2021_1952_MOESM1_ESM.pdf]

Supplementary information

A divide-and-conquer phylogenomic approach based on character supermatrices resolves early steps in the evolution of the *Archaea*

Monique Aouad[1,$], Jean-Pierre Flandrois[1], Frédéric Jauffrit[1,2], Manolo Gouy[1], Simonetta Gribaldo[3,*], and Céline Brochier-Armanet[1,*]

Affiliations:

[1] Université de Lyon, Université Lyon 1, CNRS, UMR5558, Laboratoire de Biométrie et Biologie Évolutive, 43 bd du 11 novembre 1918, F-69622, Villeurbanne, France.

[2] Technology Research Department, Innovation Unit, bioMérieux SA, Marcy Étoile, France

[3] Department of Microbiology, Unit "Evolutionary Biology of the Microbial Cell", UMR2001, Institut Pasteur, Paris, France

[$] École Supérieure de Biologie-Biochimie-Biotechnologies, Université Catholique de Lyon, 10 place des archives, 69002 Lyon, France.

***Corresponding authors:** Céline Brochier-Armanet (celine.brochier-armanet@univ-lyon1.fr)

Supplementary Figures.

Supplementary Figure S1. Unrooted Bayesian phylogeny of *Archaea*.

The tree corresponds to the A supermatrix (72 protein families, 218 taxa, 16,006 amino acids positions). The tree was inferred with PHYLOBAYES using the CAT+GTR+G4 model. Values at branch correspond to branch lengths (top) and posterior probabilities (bottom). The scale bar indicates the average number of substitutions per site.

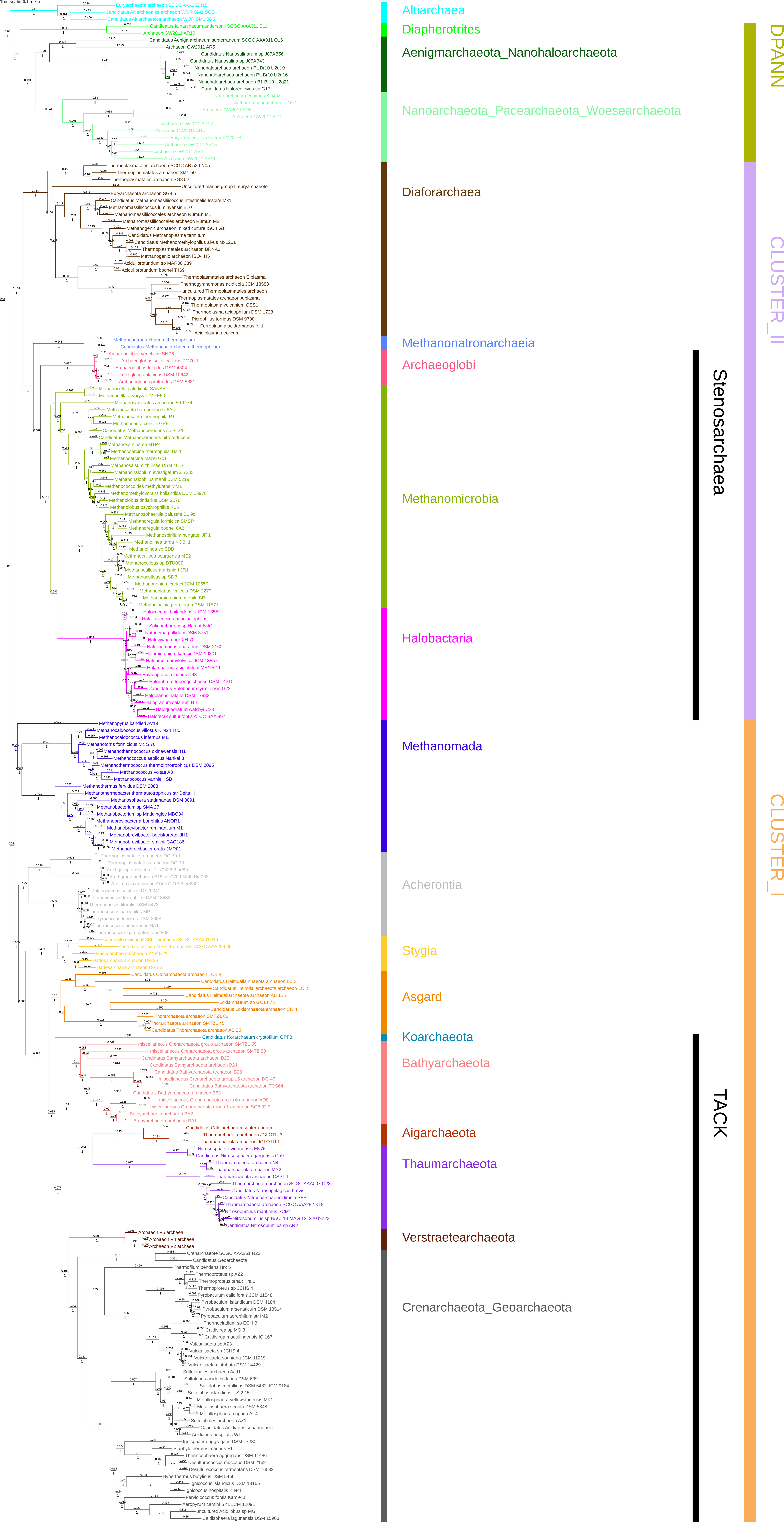

Tree scale: 0.1
Altiarchaea
Diapherotrites
Aenigmarchaeota_Nanohaloarchaeota
Nanoarchaeota_Pacearchaeota_Woesearchaeota
Diaforarchaea
Methanonatronarchaeia
Archaeoglobi
Methanomicrobia
Halobactaria
Methanomada
Acherontia
Stygia
Asgard
Koarchaeota
Bathyarchaeota
Aigarchaeota
Thaumarchaeota
Verstraetearchaeota
Crenarchaeota_Geoarchaeota
DPANN
CLUSTER_II
CLUSTER_I
Stenosarchaea
TACK

Supplementary Figure S2. Unrooted Maximum Likelihood phylogeny of *Archaea*.

The tree corresponds to the A supermatrix (72 protein families (218 taxa, 16,006 amino acids positions). The tree was inferred with IQ-TREE using the LG+C20+G4 model. Values at branch correspond to branch lengths (top) and bootstrap values (bottom). The scale bar indicates the average number of substitutions per site.

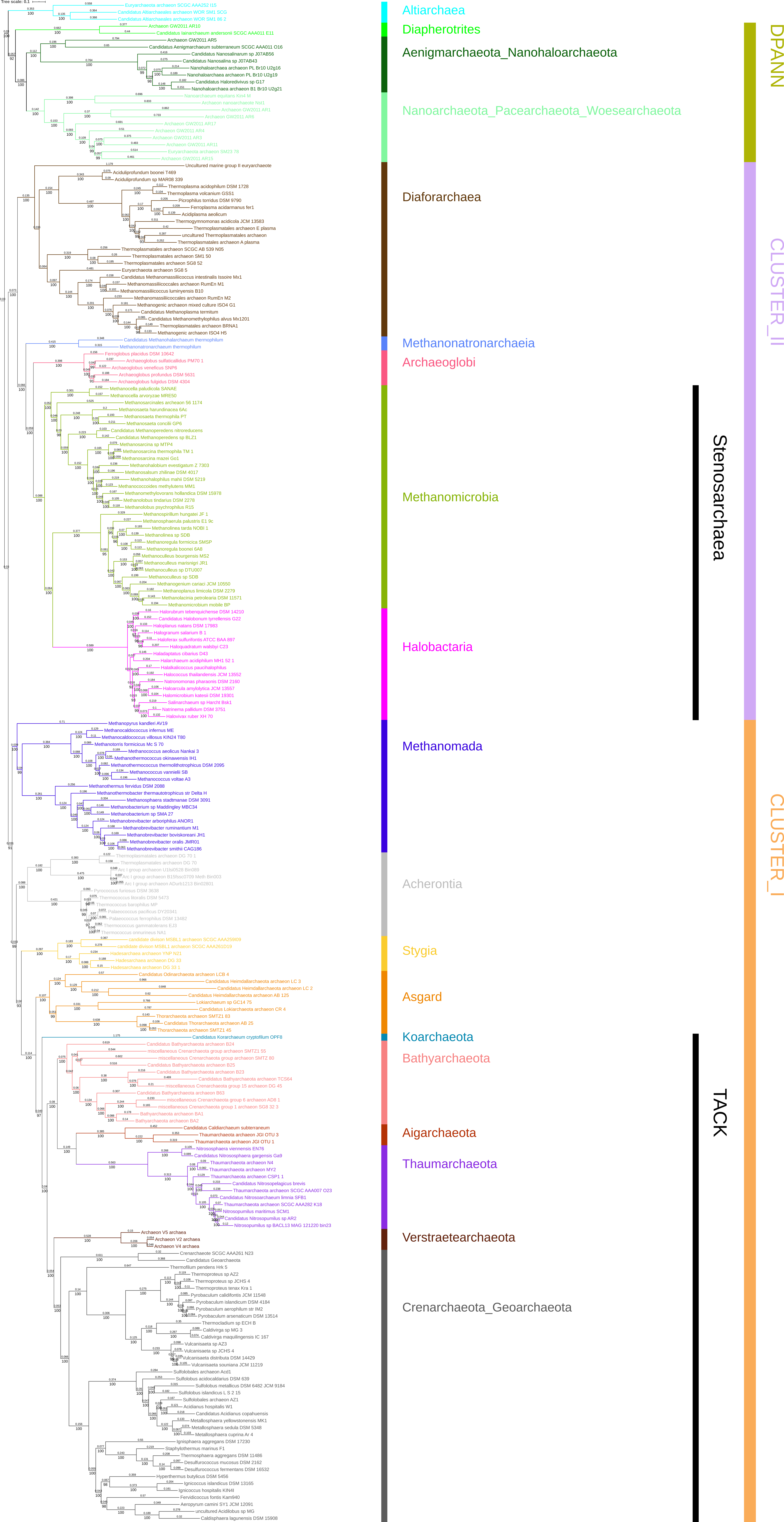

Tree scale: 0.1
Euryarchaeota archaeon SCGC AAA252 I15
Candidatus Altiarchaeales archaeon WOR SM1 SCG
Candidatus Altiarchaeales archaeon WOR SM1 86 2
Archaeon GW2011 AR10
Candidatus Iainarchaeum andersonii SCGC AAA011 E11
Archaeon GW2011 AR5
Candidatus Aenigmarchaeum subterraneum SCGC AAA011 O16
Candidatus Nanosalinarum sp J07AB56
Candidatus Nanosalina sp J07AB43
Nanohaloarchaea archaeon PL Br10 U2g16
Nanohaloarchaea archaeon PL Br10 U2g19
Candidatus Haloredivivus sp G17
Nanohaloarchaea archaeon B1 Br10 U2g21
Nanoarchaeum equitans Kin4 M
Archaeon nanoarchaeote Nst1
Archaeon GW2011 AR1
Archaeon GW2011 AR6
Archaeon GW2011 AR17
Archaeon GW2011 AR4
Archaeon GW2011 AR3
Archaeon GW2011 AR11
Euryarchaeota archaeon SM23 78
Archaeon GW2011 AR15
Uncultured marine group II euryarchaeote
Aciduliprofundum boonei T469
Aciduliprofundum sp MAR08 339
Thermoplasma acidophilum DSM 1728
Thermoplasma volcanium GSS1
Picrophilus torridus DSM 9790
Ferroplasma acidarmanus fer1
Acidiplasma aeolicum
Thermogymnomonas acidicola JCM 13583
Thermoplasmatales archaeon E plasma
uncultured Thermoplasmatales archaeon
Thermoplasmatales archaeon A plasma
Thermoplasmatales archaeon SCGC AB 539 N05
Thermoplasmatales archaeon SM1 50
Thermoplasmatales archaeon SG8 52
Euryarchaeota archaeon SG8 5
Candidatus Methanomassiliicoccus intestinalis Issoire Mx1
Methanomassiliicoccales archaeon RumEn M1
Methanomassiliicoccus luminyensis B10
Methanomassiliicoccales archaeon RumEn M2
Methanogenic archaeon mixed culture ISO4 G1
Candidatus Methanoplasma termitum
Candidatus Methanomethylophilus alvus Mx1201
Thermoplasmatales archaeon BRNA1
Methanogenic archaeon ISO4 H5
Candidatus Methanohalarchaeum thermophilum
Methanonatronarchaeum thermophilum
Ferroglobus placidus DSM 10642
Archaeoglobus sulfaticallidus PM70 1
Archaeoglobus veneficus SNP6
Archaeoglobus profundus DSM 5631
Archaeoglobus fulgidus DSM 4304
Methanocella paludicola SANAE
Methanocella arvoryzae MRE50
Methanosarcinales archaeon 56 1174
Methanosaeta harundinacea 6Ac
Methanosaeta thermophila PT
Methanosaeta concilii GP6
Candidatus Methanoperedens nitroreducens
Candidatus Methanoperedens sp BLZ1
Methanosarcina sp MTP4
Methanosarcina thermophila TM 1
Methanosarcina mazei Go1
Methanohalobium evestigatum Z 7303
Methanosalsum zhilinae DSM 4017
Methanohalophilus mahii DSM 5219
Methanococcoides methylutens MM1
Methanomethylovorans hollandica DSM 15978
Methanolobus tindarius DSM 2278
Methanolobus psychrophilus R15
Methanospirillum hungatei JF 1
Methanosphaerula palustris E1 9c
Methanolinea tarda NOBI 1
Methanolinea sp SDB
Methanoregula formicica SMSP
Methanoregula boonei 6A8
Methanoculleus bourgensis MS2
Methanoculleus marisnigri JR1
Methanoculleus sp DTU007
Methanoculleus sp SDB
Methanogenium cariaci JCM 10550
Methanoplanus limicola DSM 2279
Methanolacinia petrolearia DSM 11571
Methanomicrobium mobile BP
Halorubrum tebenquichense DSM 14210
Candidatus Halobonum tyrrellensis G22
Haloplanus natans DSM 17983
Halogranum salarium B 1
Haloferax sulfurifontis ATCC BAA 897
Haloquadratum walsbyi C23
Haladaptatus cibarius D43
Halarchaeum acidiphilum MH1 52 1
Halalkalicoccus paucihalophilus
Halococcus thailandensis JCM 13552
Natronomonas pharaonis DSM 2160
Haloarcula amylolytica JCM 13557
Halomicrobium katesii DSM 19301
Salinarchaeum sp Harcht Bsk1
Natrinema pallidum DSM 3751
Halovivax ruber XH 70
Methanopyrus kandleri AV19
Methanocaldococcus infernus ME
Methanocaldococcus villosus KIN24 T80
Methanotorris formicicus Mc S 70
Methanococcus aeolicus Nankai 3
Methanothermococcus okinawensis IH1
Methanothermococcus thermolithotrophicus DSM 2095
Methanococcus vannielii SB
Methanococcus voltae A3
Methanothermus fervidus DSM 2088
Methanothermobacter thermautotrophicus str Delta H
Methanosphaera stadtmanae DSM 3091
Methanobacterium sp Maddingley MBC34
Methanobacterium sp SMA 27
Methanobrevibacter arboriphilus ANOR1
Methanobrevibacter ruminantium M1
Methanobrevibacter boviskoreani JH1
Methanobrevibacter oralis JMR01
Methanobrevibacter smithii CAG186
Thermoplasmatales archaeon DG 70 1
Thermoplasmatales archaeon DG 70
Arc I group archaeon U1lsi0528 Bin089
Arc I group archaeon B15fssc0709 Meth Bin003
Arc I group archaeon ADurb1213 Bin02801
Pyrococcus furiosus DSM 3638
Thermococcus litoralis DSM 5473
Thermococcus barophilus MP
Palaeococcus pacificus DY20341
Palaeococcus ferrophilus DSM 13482
Thermococcus gammatolerans EJ3
Thermococcus onnurineus NA1
candidate division MSBL1 archaeon SCGC AAA259I09
candidate division MSBL1 archaeon SCGC AAA261D19
Hadesarchaea archaeon YNP N21
Hadesarchaea archaeon DG 33
Hadesarchaea archaeon DG 33 1
Candidatus Odinarchaeota archaeon LCB 4
Candidatus Heimdallarchaeota archaeon LC 3
Candidatus Heimdallarchaeota archaeon LC 2
Candidatus Heimdallarchaeota archaeon AB 125
Lokiarchaeum sp GC14 75
Candidatus Lokiarchaeota archaeon CR 4
Thorarchaeota archaeon SMTZ1 83
Candidatus Thorarchaeota archaeon AB 25
Thorarchaeota archaeon SMTZ1 45
Candidatus Korarchaeum cryptofilum OPF8
Candidatus Bathyarchaeota archaeon B24
miscellaneous Crenarchaeota group archaeon SMTZ1 55
miscellaneous Crenarchaeota group archaeon SMTZ 80
Candidatus Bathyarchaeota archaeon B25
Candidatus Bathyarchaeota archaeon B23
Candidatus Bathyarchaeota archaeon TCS64
miscellaneous Crenarchaeota group 15 archaeon DG 45
Candidatus Bathyarchaeota archaeon B63
miscellaneous Crenarchaeota group 6 archaeon AD8 1
miscellaneous Crenarchaeota group 1 archaeon SG8 32 3
Bathyarchaeota archaeon BA1
Bathyarchaeota archaeon BA2
Candidatus Caldiarchaeum subterraneum
Thaumarchaeota archaeon JGI OTU 3
Thaumarchaeota archaeon JGI OTU 1
Nitrososphaera viennensis EN76
Candidatus Nitrososphaera gargensis Ga9
Thaumarchaeota archaeon N4
Thaumarchaeota archaeon MY2
Thaumarchaeota archaeon CSP1 1
Candidatus Nitrosopelagicus brevis
Thaumarchaeota archaeon SCGC AAA007 O23
Candidatus Nitrosoarchaeum limnia SFB1
Thaumarchaeota archaeon SCGC AAA282 K18
Nitrosopumilus maritimus SCM1
Candidatus Nitrosopumilus sp AR2
Nitrosopumilus sp BACL13 MAG 121220 bin23
Archaeon V5 archaea
Archaeon V2 archaea
Archaeon V4 archaea
Crenarchaeote SCGC AAA261 N23
Candidatus Geoarchaeota
Thermofilum pendens Hrk 5
Thermoproteus sp AZ2
Thermoproteus sp JCHS 4
Thermoproteus tenax Kra 1
Pyrobaculum calidifontis JCM 11548
Pyrobaculum islandicum DSM 4184
Pyrobaculum aerophilum str IM2
Pyrobaculum arsenaticum DSM 13514
Thermocladium sp ECH B
Caldivirga sp MG 3
Caldivirga maquilingensis IC 167
Vulcanisaeta sp AZ3
Vulcanisaeta sp JCHS 4
Vulcanisaeta distributa DSM 14429
Vulcanisaeta souniana JCM 11219
Sulfolobales archaeon Acd1
Sulfolobus acidocaldarius DSM 639
Sulfolobus metallicus DSM 6482 JCM 9184
Sulfolobus islandicus L S 2 15
Sulfolobales archaeon AZ1
Acidianus hospitalis W1
Candidatus Acidianus copahuensis
Metallosphaera yellowstonensis MK1
Metallosphaera sedula DSM 5348
Metallosphaera cuprina Ar 4
Ignisphaera aggregans DSM 17230
Staphylothermus marinus F1
Thermosphaera aggregans DSM 11486
Desulfurococcus mucosus DSM 2162
Desulfurococcus fermentans DSM 16532
Hyperthermus butylicus DSM 5456
Ignicoccus islandicus DSM 13165
Ignicoccus hospitalis KIN4I
Fervidicoccus fontis Kam940
Aeropyrum camini SY1 JCM 12091
uncultured Acidilobus sp MG
Caldisphaera lagunensis DSM 15908
Altiarchaea
Diapherotrites
Aenigmarchaeota_Nanohaloarchaeota
Nanoarchaeota_Pacearchaeota_Woesearchaeota
Diaforarchaea
Methanonatronarchaeia
Archaeoglobi
Methanomicrobia
Halobacteria
Methanomada
Acherontia
Stygia
Asgard
Koarchaeota
Bathyarchaeota
Aigarchaeota
Thaumarchaeota
Verstraetearchaeota
Crenarchaeota_Geoarchaeota
DPANN
CLUSTER_II
CLUSTER_I
Stenosarchaea
TACK

Supplementary Figure S3. Unrooted Bayesian phylogeny of *Archaea* inferred through the Slow-Fast procedure.

Starting from the A supermatrix (72 protein families, 218 taxa, 16,006 amino acids positions), the fastest evolving sites were removed progressively. At each step, a Bayesian tree was inferred with PHYLOBAYES using the CAT+GTR+G4 model. Values at branch correspond to posterior probabilities. The scale bars indicate the average number of substitutions per site.

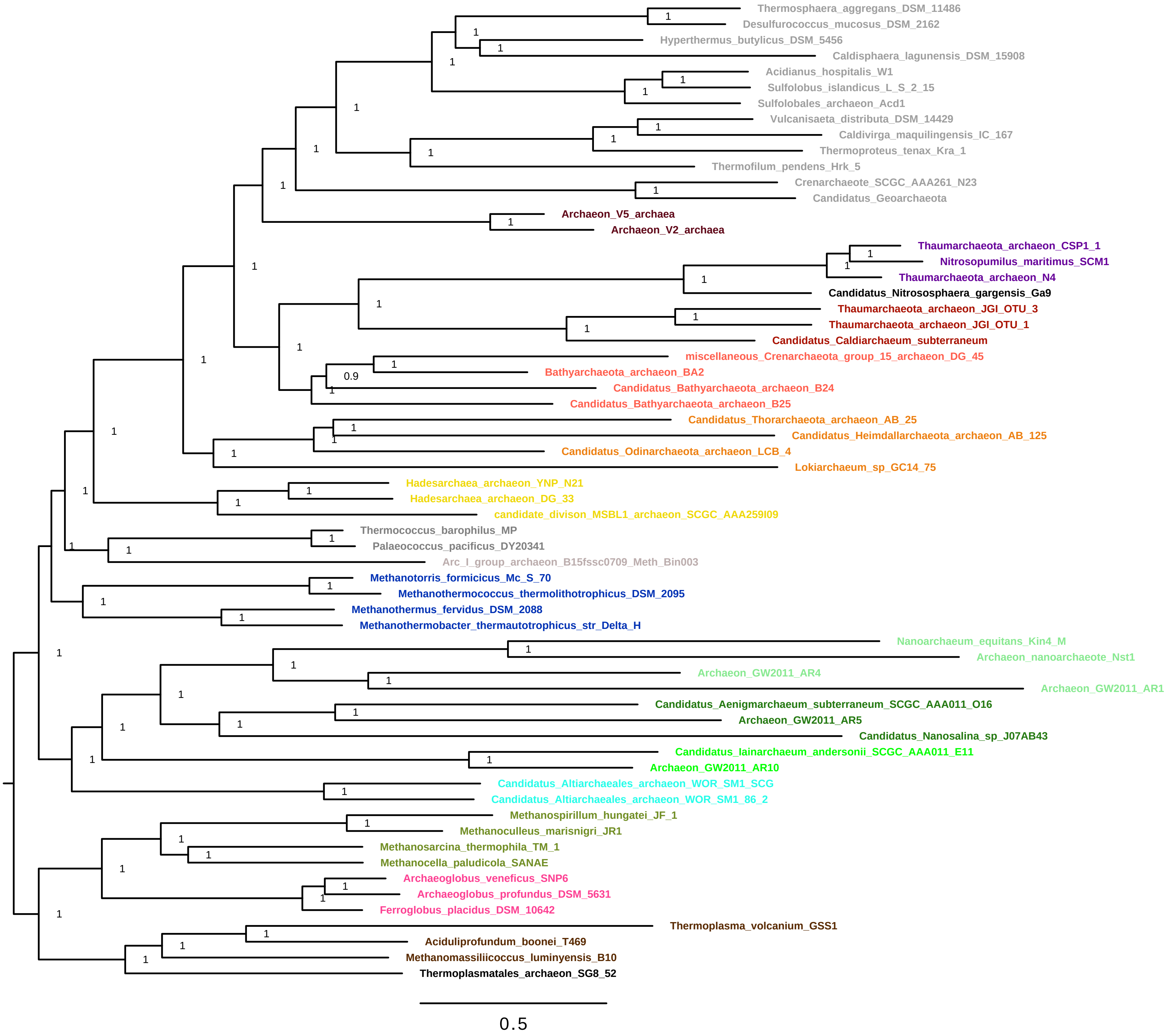
Thermosphaera_aggregans_DSM_11486
Desulfurococcus_mucosus_DSM_2162
Hyperthermus_butylicus_DSM_5456
Caldisphaera_lagunensis_DSM_15908
Acidianus_hospitalis_W1
Sulfolobus_islandicus_L_S_2_15
Sulfolobales_archaeon_Acd1
Vulcanisaeta_distributa_DSM_14429
Caldivirga_maquilingensis_IC_167
Thermoproteus_tenax_Kra_1
Thermofilum_pendens_Hrk_5
Crenarchaeote_SCGC_AAA261_N23
Candidatus_Geoarchaeota
Archaeon_V5_archaea
Archaeon_V2_archaea
Thaumarchaeota_archaeon_CSP1_1
Nitrosopumilus_maritimus_SCM1
Thaumarchaeota_archaeon_N4
Candidatus_Nitrososphaera_gargensis_Ga9
Thaumarchaeota_archaeon_JGI_OTU_3
Thaumarchaeota_archaeon_JGI_OTU_1
Candidatus_Caldiarchaeum_subterraneum
miscellaneous_Crenarchaeota_group_15_archaeon_DG_45
Bathyarchaeota_archaeon_BA2
Candidatus_Bathyarchaeota_archaeon_B24
Candidatus_Bathyarchaeota_archaeon_B25
Candidatus_Thorarchaeota_archaeon_AB_25
Candidatus_Heimdallarchaeota_archaeon_AB_125
Candidatus_Odinarchaeota_archaeon_LCB_4
Lokiarchaeum_sp_GC14_75
Hadesarchaea_archaeon_YNP_N21
Hadesarchaea_archaeon_DG_33
candidate_divison_MSBL1_archaeon_SCGC_AAA259I09
Thermococcus_barophilus_MP
Palaeococcus_pacificus_DY20341
Arc_I_group_archaeon_B15fssc0709_Meth_Bin003
Methanotorris_formicicus_Mc_S_70
Methanothermococcus_thermolithotrophicus_DSM_2095
Methanothermus_fervidus_DSM_2088
Methanothermobacter_thermautotrophicus_str_Delta_H
Nanoarchaeum_equitans_Kin4_M
Archaeon_nanoarchaeote_Nst1
Archaeon_GW2011_AR4
Archaeon_GW2011_AR1
Candidatus_Aenigmarchaeum_subterraneum_SCGC_AAA011_O16
Archaeon_GW2011_AR5
Candidatus_Nanosalina_sp_J07AB43
Candidatus_Iainarchaeum_andersonii_SCGC_AAA011_E11
Archaeon_GW2011_AR10
Candidatus_Altiarchaeales_archaeon_WOR_SM1_SCG
Candidatus_Altiarchaeales_archaeon_WOR_SM1_86_2
Methanospirillum_hungatei_JF_1
Methanoculleus_marisnigri_JR1
Methanosarcina_thermophila_TM_1
Methanocella_paludicola_SANAE
Archaeoglobus_veneficus_SNP6
Archaeoglobus_profundus_DSM_5631
Ferroglobus_placidus_DSM_10642
Thermoplasma_volcanium_GSS1
Aciduliprofundum_boonei_T469
Methanomassiliicoccus_luminyensis_B10
Thermoplasmatales_archaeon_SG8_52
0.5

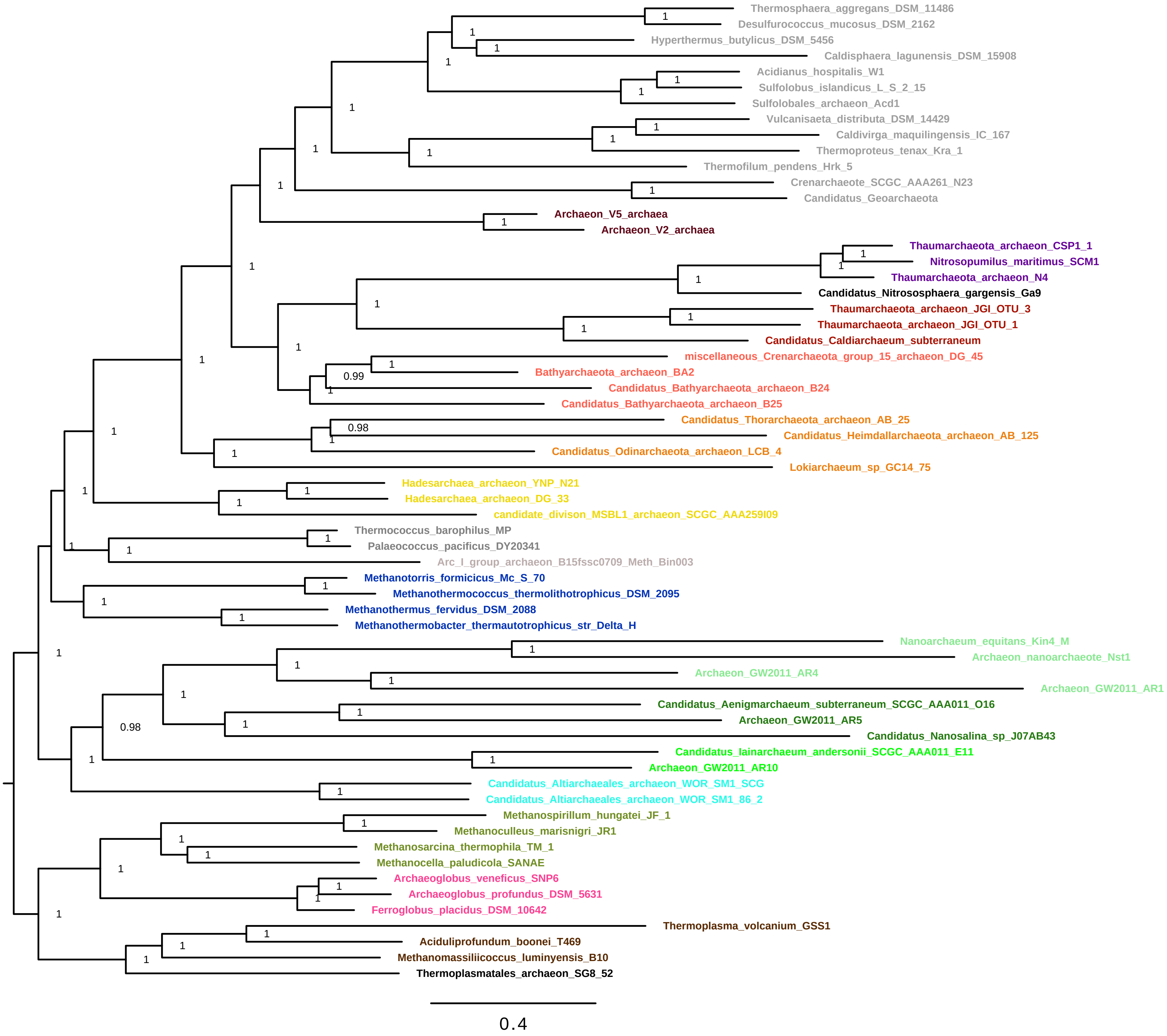
Thermosphaera_aggregans_DSM_11486
Desulfurococcus_mucosus_DSM_2162
Hyperthermus_butylicus_DSM_5456
Caldisphaera_lagunensis_DSM_15908
Acidianus_hospitalis_W1
Sulfolobus_islandicus_L_S_2_15
Sulfolobales_archaeon_Acd1
Vulcanisaeta_distributa_DSM_14429
Caldivirga_maquilingensis_IC_167
Thermoproteus_tenax_Kra_1
Thermofilum_pendens_Hrk_5
Crenarchaeote_SCGC_AAA261_N23
Candidatus_Geoarchaeota
Archaeon_V5_archaea
Archaeon_V2_archaea
Thaumarchaeota_archaeon_CSP1_1
Nitrosopumilus_maritimus_SCM1
Thaumarchaeota_archaeon_N4
Candidatus_Nitrososphaera_gargensis_Ga9
Thaumarchaeota_archaeon_JGI_OTU_3
Thaumarchaeota_archaeon_JGI_OTU_1
Candidatus_Caldiarchaeum_subterraneum
miscellaneous_Crenarchaeota_group_15_archaeon_DG_45
Bathyarchaeota_archaeon_BA2
Candidatus_Bathyarchaeota_archaeon_B24
Candidatus_Bathyarchaeota_archaeon_B25
Candidatus_Thorarchaeota_archaeon_AB_25
Candidatus_Heimdallarchaeota_archaeon_AB_125
Candidatus_Odinarchaeota_archaeon_LCB_4
Lokiarchaeum_sp_GC14_75
Hadesarchaea_archaeon_YNP_N21
Hadesarchaea_archaeon_DG_33
candidate_divison_MSBL1_archaeon_SCGC_AAA259I09
Thermococcus_barophilus_MP
Palaeococcus_pacificus_DY20341
Arc_I_group_archaeon_B15fssc0709_Meth_Bin003
Methanotorris_formicicus_Mc_S_70
Methanothermococcus_thermolithotrophicus_DSM_2095
Methanothermus_fervidus_DSM_2088
Methanothermobacter_thermautotrophicus_str_Delta_H
Nanoarchaeum_equitans_Kin4_M
Archaeon_nanoarchaeote_Nst1
Archaeon_GW2011_AR4
Archaeon_GW2011_AR1
Candidatus_Aenigmarchaeum_subterraneum_SCGC_AAA011_O16
Archaeon_GW2011_AR5
Candidatus_Nanosalina_sp_J07AB43
Candidatus_Iainarchaeum_andersonii_SCGC_AAA011_E11
Archaeon_GW2011_AR10
Candidatus_Altiarchaeales_archaeon_WOR_SM1_SCG
Candidatus_Altiarchaeales_archaeon_WOR_SM1_86_2
Methanospirillum_hungatei_JF_1
Methanoculleus_marisnigri_JR1
Methanosarcina_thermophila_TM_1
Methanocella_paludicola_SANAE
Archaeoglobus_veneficus_SNP6
Archaeoglobus_profundus_DSM_5631
Ferroglobus_placidus_DSM_10642
Thermoplasma_volcanium_GSS1
Aciduliprofundum_boonei_T469
Methanomassiliicoccus_luminyensis_B10
Thermoplasmatales_archaeon_SG8_52
0.4

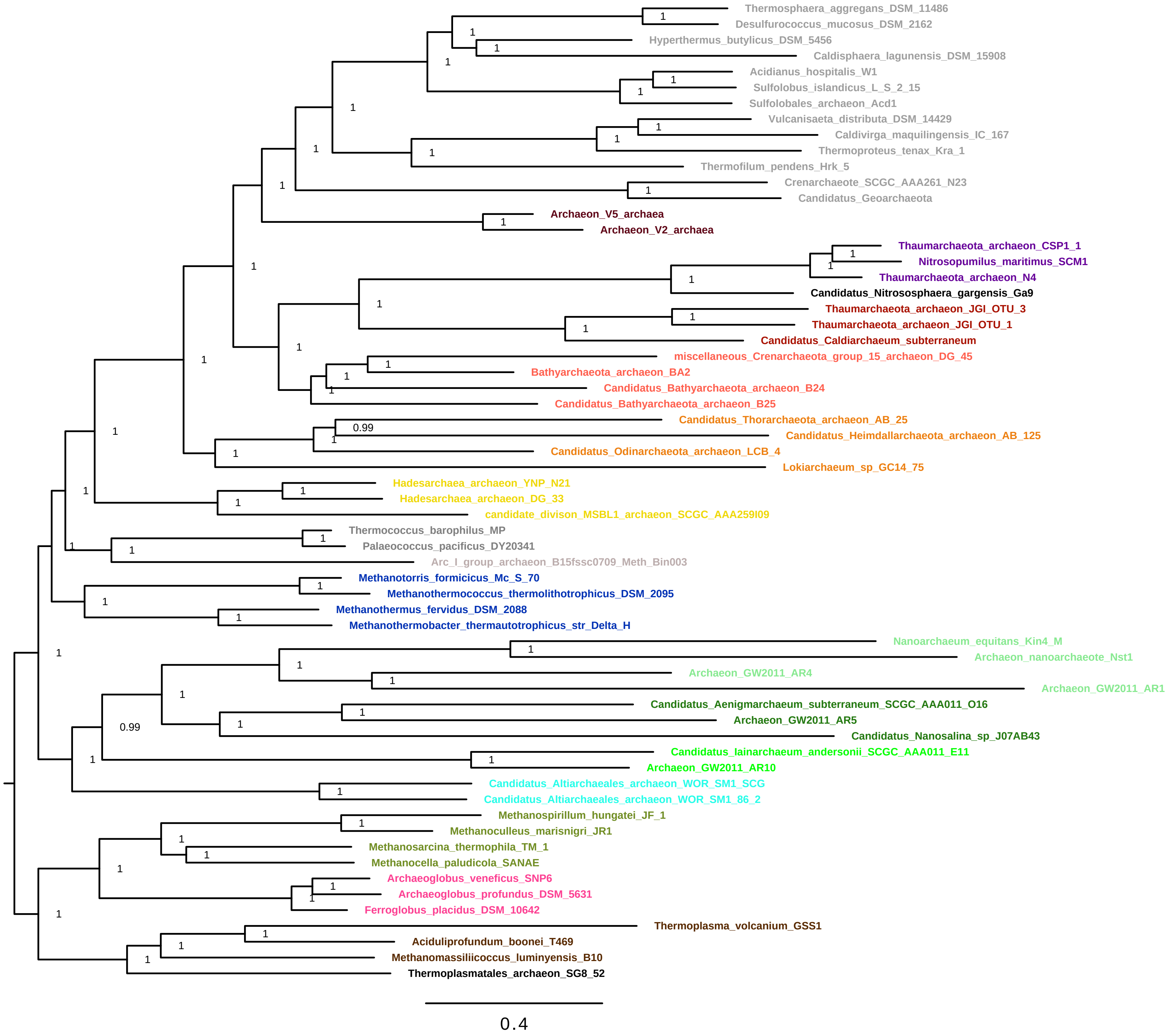

Thermosphaera_aggregans_DSM_11486
Desulfurococcus_mucosus_DSM_2162
Hyperthermus_butylicus_DSM_5456
Caldisphaera_lagunensis_DSM_15908
Acidianus_hospitalis_W1
Sulfolobus_islandicus_L_S_2_15
Sulfolobales_archaeon_Acd1
Vulcanisaeta_distributa_DSM_14429
Caldivirga_maquilingensis_IC_167
Thermoproteus_tenax_Kra_1
Thermofilum_pendens_Hrk_5
Crenarchaeote_SCGC_AAA261_N23
Candidatus_Geoarchaeota
Archaeon_V5_archaea
Archaeon_V2_archaea
Thaumarchaeota_archaeon_CSP1_1
Nitrosopumilus_maritimus_SCM1
Thaumarchaeota_archaeon_N4
Candidatus_Nitrososphaera_gargensis_Ga9
Thaumarchaeota_archaeon_JGI_OTU_3
Thaumarchaeota_archaeon_JGI_OTU_1
Candidatus_Caldiarchaeum_subterraneum
miscellaneous_Crenarchaeota_group_15_archaeon_DG_45
Bathyarchaeota_archaeon_BA2
Candidatus_Bathyarchaeota_archaeon_B24
Candidatus_Bathyarchaeota_archaeon_B25
Candidatus_Thorarchaeota_archaeon_AB_25
Candidatus_Heimdallarchaeota_archaeon_AB_125
Candidatus_Odinarchaeota_archaeon_LCB_4
Lokiarchaeum_sp_GC14_75
Hadesarchaea_archaeon_YNP_N21
Hadesarchaea_archaeon_DG_33
candidate_divison_MSBL1_archaeon_SCGC_AAA259I09
Thermococcus_barophilus_MP
Palaeococcus_pacificus_DY20341
Arc_I_group_archaeon_B15fssc0709_Meth_Bin003
Methanotorris_formicicus_Mc_S_70
Methanothermococcus_thermolithotrophicus_DSM_2095
Methanothermus_fervidus_DSM_2088
Methanothermobacter_thermautotrophicus_str_Delta_H
Nanoarchaeum_equitans_Kin4_M
Archaeon_nanoarchaeote_Nst1
Archaeon_GW2011_AR4
Archaeon_GW2011_AR1
Candidatus_Aenigmarchaeum_subterraneum_SCGC_AAA011_O16
Archaeon_GW2011_AR5
Candidatus_Nanosalina_sp_J07AB43
Candidatus_Iainarchaeum_andersonii_SCGC_AAA011_E11
Archaeon_GW2011_AR10
Candidatus_Altiarchaeales_archaeon_WOR_SM1_SCG
Candidatus_Altiarchaeales_archaeon_WOR_SM1_86_2
Methanospirillum_hungatei_JF_1
Methanoculleus_marisnigri_JR1
Methanosarcina_thermophila_TM_1
Methanocella_paludicola_SANAE
Archaeoglobus_veneficus_SNP6
Archaeoglobus_profundus_DSM_5631
Ferroglobus_placidus_DSM_10642
Thermoplasma_volcanium_GSS1
Aciduliprofundum_boonei_T469
Methanomassiliicoccus_luminyensis_B10
Thermoplasmatales_archaeon_SG8_52
0.99
0.99
0.4

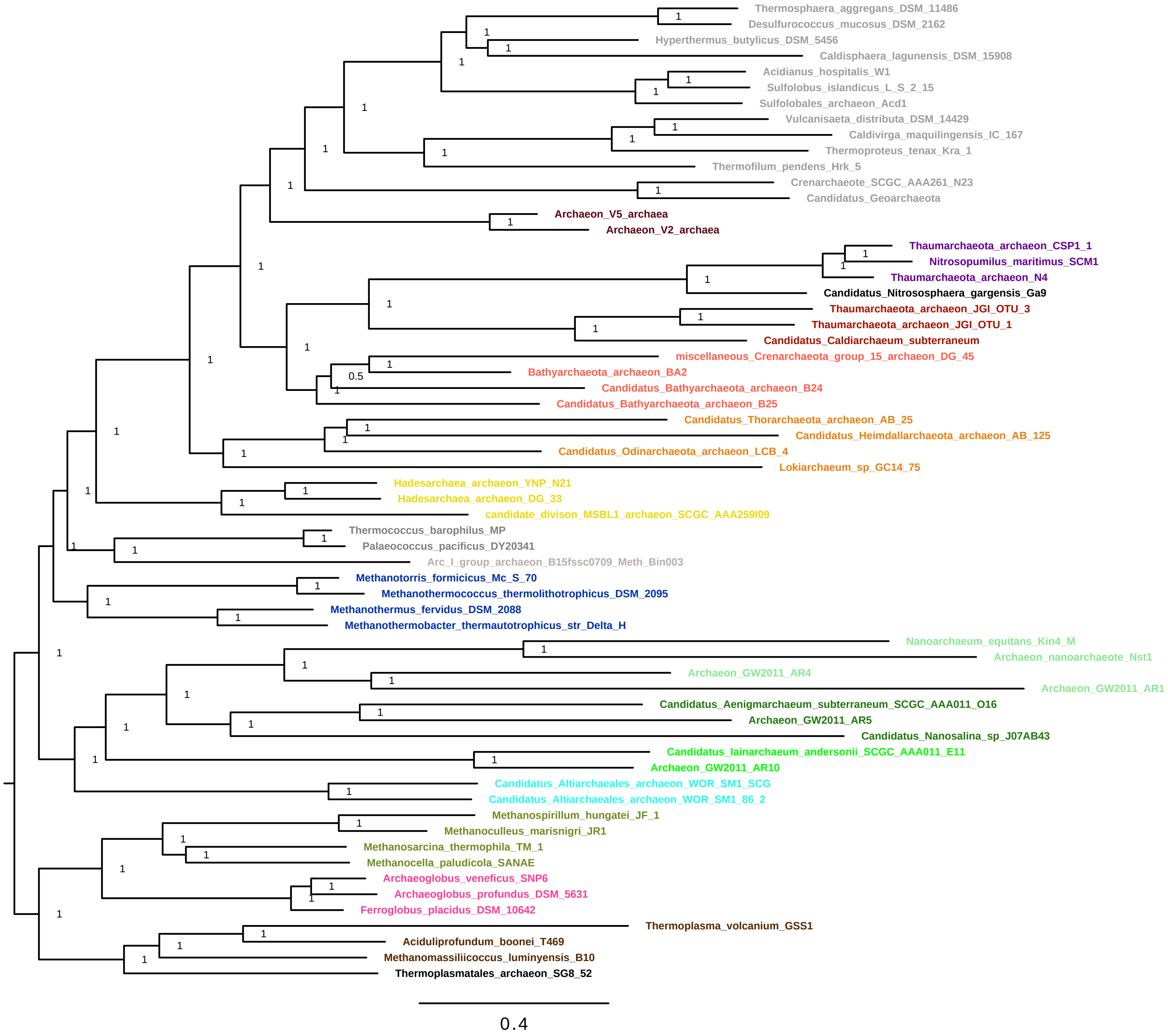

Thermosphaera_aggregans_DSM_11486
Desulfurococcus_mucosus_DSM_2162
Hyperthermus_butylicus_DSM_5456
Caldisphaera_lagunensis_DSM_15908
Acidianus_hospitalis_W1
Sulfolobus_islandicus_L_S_2_15
Sulfolobales_archaeon_Acd1
Vulcanisaeta_distributa_DSM_14429
Caldivirga_maquilingensis_IC_167
Thermoproteus_tenax_Kra_1
Thermofilum_pendens_Hrk_5
Crenarchaeote_SCGC_AAA261_N23
Candidatus_Geoarchaeota
Archaeon_V5_archaea
Archaeon_V2_archaea
Thaumarchaeota_archaeon_CSP1_1
Nitrosopumilus_maritimus_SCM1
Thaumarchaeota_archaeon_N4
Candidatus_Nitrososphaera_gargensis_Ga9
Thaumarchaeota_archaeon_JGI_OTU_3
Thaumarchaeota_archaeon_JGI_OTU_1
Candidatus_Caldiarchaeum_subterraneum
miscellaneous_Crenarchaeota_group_15_archaeon_DG_45
Bathyarchaeota_archaeon_BA2
Candidatus_Bathyarchaeota_archaeon_B24
Candidatus_Bathyarchaeota_archaeon_B25
Candidatus_Thorarchaeota_archaeon_AB_25
Candidatus_Heimdallarchaeota_archaeon_AB_125
Candidatus_Odinarchaeota_archaeon_LCB_4
Lokiarchaeum_sp_GC14_75
Hadesarchaea_archaeon_YNP_N21
Hadesarchaea_archaeon_DG_33
candidate_divison_MSBL1_archaeon_SCGC_AAA259I09
Thermococcus_barophilus_MP
Palaeococcus_pacificus_DY20341
Arc_I_group_archaeon_B15fssc0709_Meth_Bin003
Methanotorris_formicicus_Mc_S_70
Methanothermococcus_thermolithotrophicus_DSM_2095
Methanothermus_fervidus_DSM_2088
Methanothermobacter_thermautotrophicus_str_Delta_H
Nanoarchaeum_equitans_Kin4_M
Archaeon_nanoarchaeote_Nst1
Archaeon_GW2011_AR4
Archaeon_GW2011_AR1
Candidatus_Aenigmarchaeum_subterraneum_SCGC_AAA011_O16
Archaeon_GW2011_AR5
Candidatus_Nanosalina_sp_J07AB43
Candidatus_Iainarchaeum_andersonii_SCGC_AAA011_E11
Archaeon_GW2011_AR10
Candidatus_Altiarchaeales_archaeon_WOR_SM1_SCG
Candidatus_Altiarchaeales_archaeon_WOR_SM1_86_2
Methanospirillum_hungatei_JF_1
Methanoculleus_marisnigri_JR1
Methanosarcina_thermophila_TM_1
Methanocella_paludicola_SANAE
Archaeoglobus_veneficus_SNP6
Archaeoglobus_profundus_DSM_5631
Ferroglobus_placidus_DSM_10642
Thermoplasma_volcanium_GSS1
Aciduliprofundum_boonei_T469
Methanomassiliicoccus_luminyensis_B10
Thermoplasmatales_archaeon_SG8_52
0.5
0.4

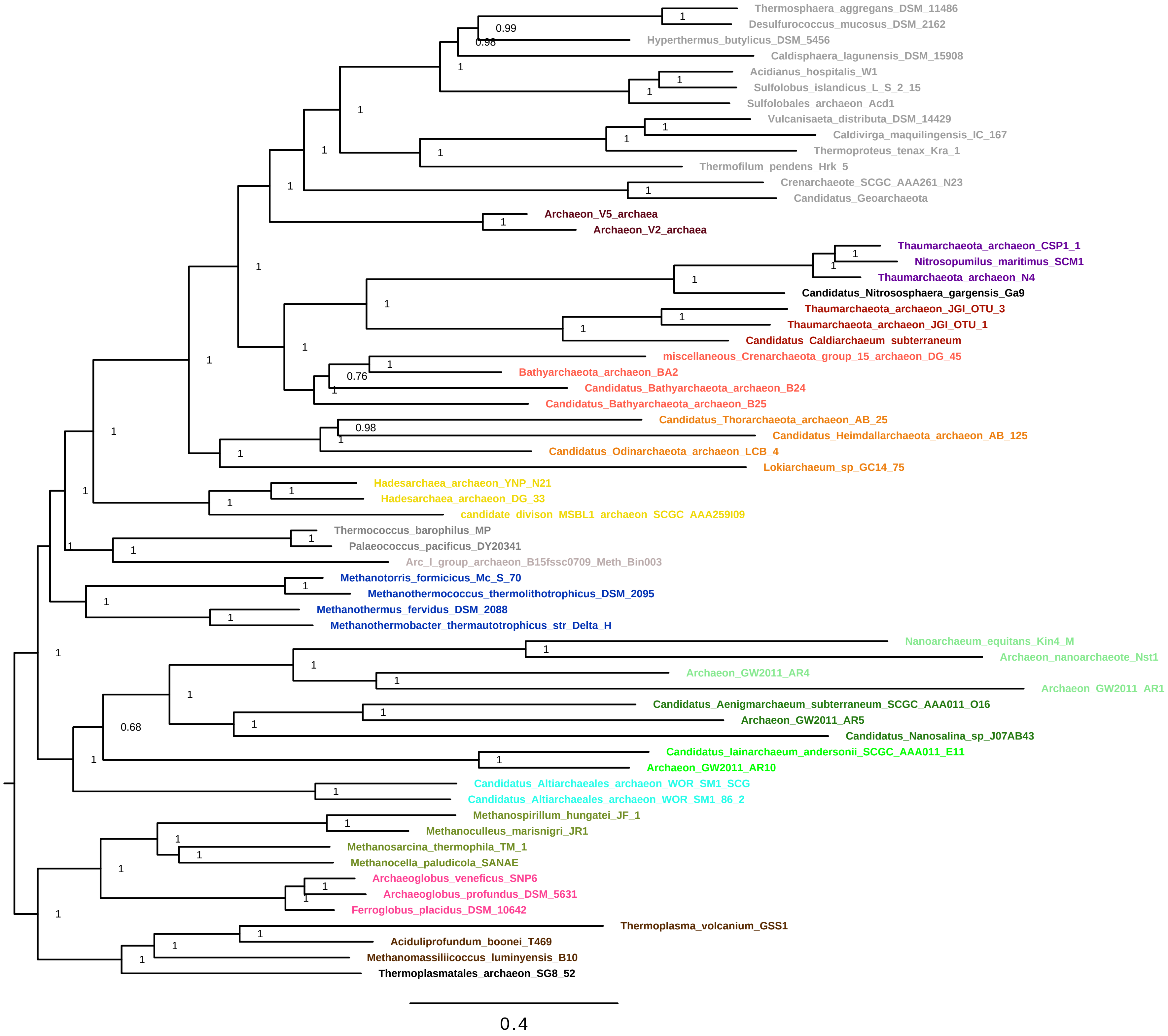

Thermosphaera_aggregans_DSM_11486
Desulfurococcus_mucosus_DSM_2162
Hyperthermus_butylicus_DSM_5456
Caldisphaera_lagunensis_DSM_15908
Acidianus_hospitalis_W1
Sulfolobus_islandicus_L_S_2_15
Sulfolobales_archaeon_Acd1
Vulcanisaeta_distributa_DSM_14429
Caldivirga_maquilingensis_IC_167
Thermoproteus_tenax_Kra_1
Thermofilum_pendens_Hrk_5
Crenarchaeote_SCGC_AAA261_N23
Candidatus_Geoarchaeota
Archaeon_V5_archaea
Archaeon_V2_archaea
Thaumarchaeota_archaeon_CSP1_1
Nitrosopumilus_maritimus_SCM1
Thaumarchaeota_archaeon_N4
Candidatus_Nitrososphaera_gargensis_Ga9
Thaumarchaeota_archaeon_JGI_OTU_3
Thaumarchaeota_archaeon_JGI_OTU_1
Candidatus_Caldiarchaeum_subterraneum
miscellaneous_Crenarchaeota_group_15_archaeon_DG_45
Bathyarchaeota_archaeon_BA2
Candidatus_Bathyarchaeota_archaeon_B24
Candidatus_Bathyarchaeota_archaeon_B25
Candidatus_Thorarchaeota_archaeon_AB_25
Candidatus_Heimdallarchaeota_archaeon_AB_125
Candidatus_Odinarchaeota_archaeon_LCB_4
Lokiarchaeum_sp_GC14_75
Hadesarchaea_archaeon_YNP_N21
Hadesarchaea_archaeon_DG_33
candidate_divison_MSBL1_archaeon_SCGC_AAA259I09
Thermococcus_barophilus_MP
Palaeococcus_pacificus_DY20341
Arc_I_group_archaeon_B15fssc0709_Meth_Bin003
Methanotorris_formicicus_Mc_S_70
Methanothermococcus_thermolithotrophicus_DSM_2095
Methanothermus_fervidus_DSM_2088
Methanothermobacter_thermautotrophicus_str_Delta_H
Nanoarchaeum_equitans_Kin4_M
Archaeon_nanoarchaeote_Nst1
Archaeon_GW2011_AR4
Archaeon_GW2011_AR1
Candidatus_Aenigmarchaeum_subterraneum_SCGC_AAA011_O16
Archaeon_GW2011_AR5
Candidatus_Nanosalina_sp_J07AB43
Candidatus_Iainarchaeum_andersonii_SCGC_AAA011_E11
Archaeon_GW2011_AR10
Candidatus_Altiarchaeales_archaeon_WOR_SM1_SCG
Candidatus_Altiarchaeales_archaeon_WOR_SM1_86_2
Methanospirillum_hungatei_JF_1
Methanoculleus_marisnigri_JR1
Methanosarcina_thermophila_TM_1
Methanocella_paludicola_SANAE
Archaeoglobus_veneficus_SNP6
Archaeoglobus_profundus_DSM_5631
Ferroglobus_placidus_DSM_10642
Thermoplasma_volcanium_GSS1
Aciduliprofundum_boonei_T469
Methanomassiliicoccus_luminyensis_B10
Thermoplasmatales_archaeon_SG8_52
0.99
0.98
0.76
0.68
0.4

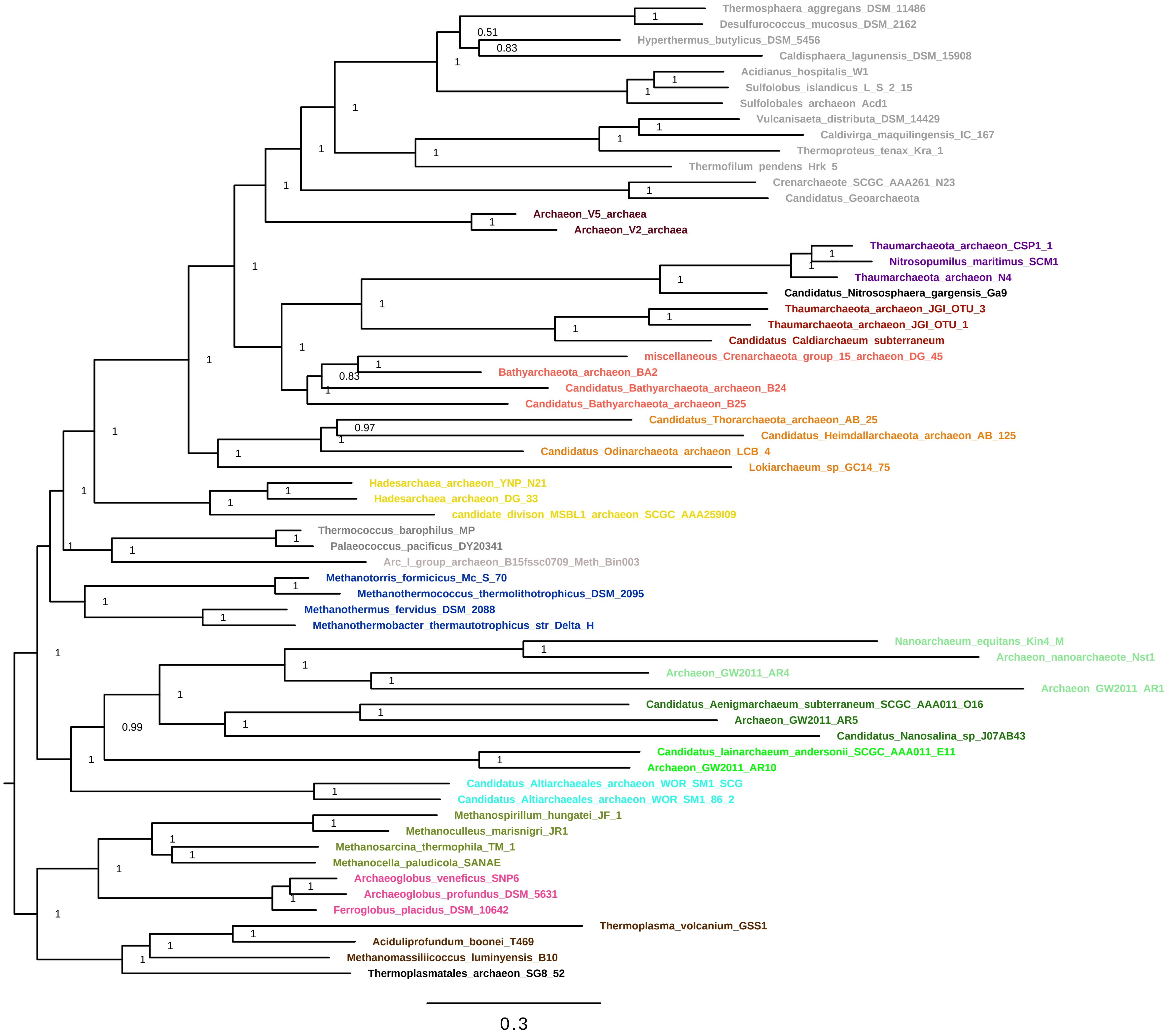
Thermosphaera_aggregans_DSM_11486
Desulfurococcus_mucosus_DSM_2162
Hyperthermus_butylicus_DSM_5456
Caldisphaera_lagunensis_DSM_15908
Acidianus_hospitalis_W1
Sulfolobus_islandicus_L_S_2_15
Sulfolobales_archaeon_Acd1
Vulcanisaeta_distributa_DSM_14429
Caldivirga_maquilingensis_IC_167
Thermoproteus_tenax_Kra_1
Thermofilum_pendens_Hrk_5
Crenarchaeote_SCGC_AAA261_N23
Candidatus_Geoarchaeota
Archaeon_V5_archaea
Archaeon_V2_archaea
Thaumarchaeota_archaeon_CSP1_1
Nitrosopumilus_maritimus_SCM1
Thaumarchaeota_archaeon_N4
Candidatus_Nitrososphaera_gargensis_Ga9
Thaumarchaeota_archaeon_JGI_OTU_3
Thaumarchaeota_archaeon_JGI_OTU_1
Candidatus_Caldiarchaeum_subterraneum
miscellaneous_Crenarchaeota_group_15_archaeon_DG_45
Bathyarchaeota_archaeon_BA2
Candidatus_Bathyarchaeota_archaeon_B24
Candidatus_Bathyarchaeota_archaeon_B25
Candidatus_Thorarchaeota_archaeon_AB_25
Candidatus_Heimdallarchaeota_archaeon_AB_125
Candidatus_Odinarchaeota_archaeon_LCB_4
Lokiarchaeum_sp_GC14_75
Hadesarchaea_archaeon_YNP_N21
Hadesarchaea_archaeon_DG_33
candidate_divison_MSBL1_archaeon_SCGC_AAA259I09
Thermococcus_barophilus_MP
Palaeococcus_pacificus_DY20341
Arc_I_group_archaeon_B15fssc0709_Meth_Bin003
Methanotorris_formicicus_Mc_S_70
Methanothermococcus_thermolithotrophicus_DSM_2095
Methanothermus_fervidus_DSM_2088
Methanothermobacter_thermautotrophicus_str_Delta_H
Nanoarchaeum_equitans_Kin4_M
Archaeon_nanoarchaeote_Nst1
Archaeon_GW2011_AR4
Archaeon_GW2011_AR1
Candidatus_Aenigmarchaeum_subterraneum_SCGC_AAA011_O16
Archaeon_GW2011_AR5
Candidatus_Nanosalina_sp_J07AB43
Candidatus_Iainarchaeum_andersonii_SCGC_AAA011_E11
Archaeon_GW2011_AR10
Candidatus_Altiarchaeales_archaeon_WOR_SM1_SCG
Candidatus_Altiarchaeales_archaeon_WOR_SM1_86_2
Methanospirillum_hungatei_JF_1
Methanoculleus_marisnigri_JR1
Methanosarcina_thermophila_TM_1
Methanocella_paludicola_SANAE
Archaeoglobus_veneficus_SNP6
Archaeoglobus_profundus_DSM_5631
Ferroglobus_placidus_DSM_10642
Thermoplasma_volcanium_GSS1
Aciduliprofundum_boonei_T469
Methanomassiliicoccus_luminyensis_B10
Thermoplasmatales_archaeon_SG8_52
0.3

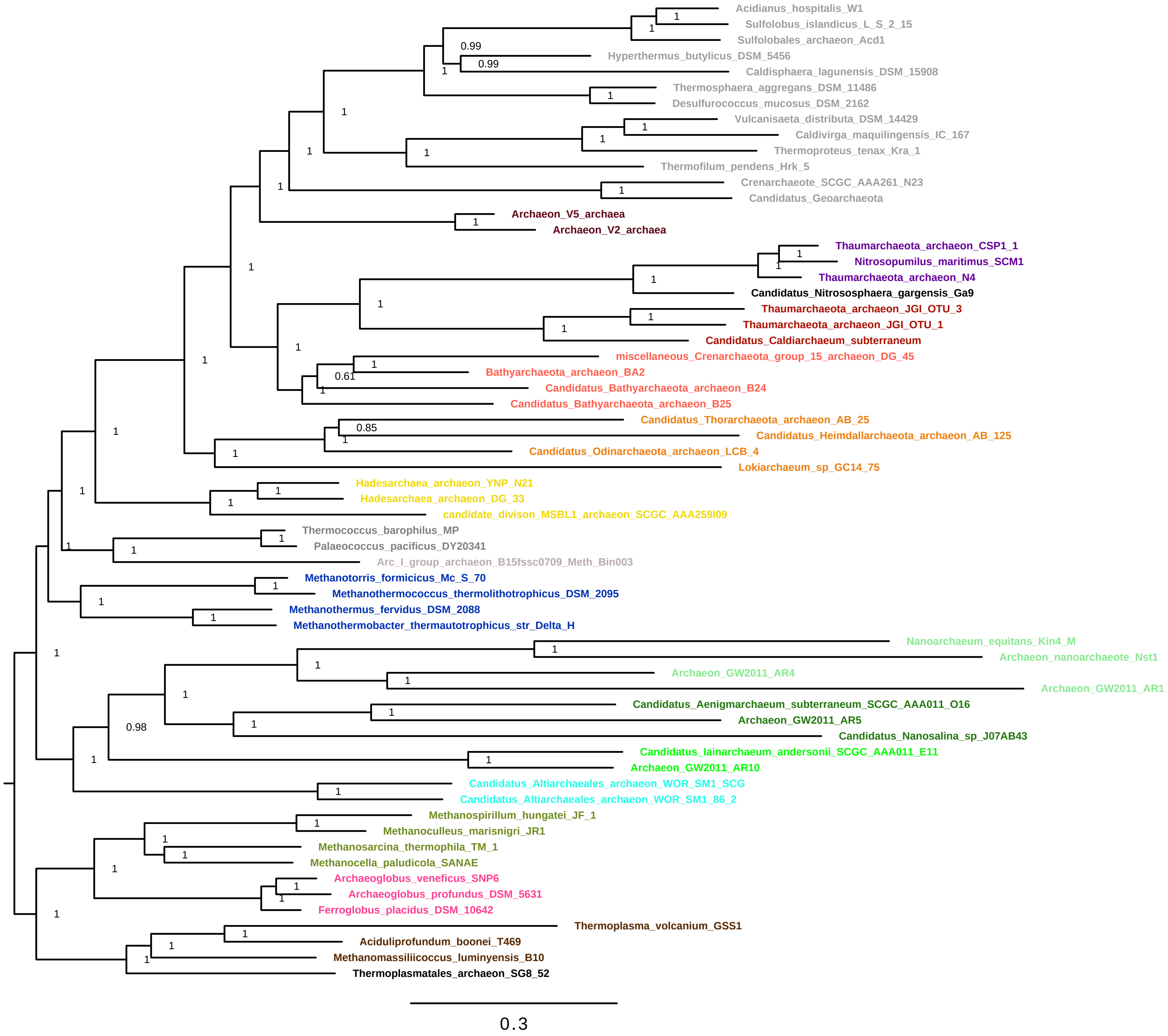

Acidianus_hospitalis_W1
Sulfolobus_islandicus_L_S_2_15
Sulfolobales_archaeon_Acd1
Hyperthermus_butylicus_DSM_5456
Caldisphaera_lagunensis_DSM_15908
Thermosphaera_aggregans_DSM_11486
Desulfurococcus_mucosus_DSM_2162
Vulcanisaeta_distributa_DSM_14429
Caldivirga_maquilingensis_IC_167
Thermoproteus_tenax_Kra_1
Thermofilum_pendens_Hrk_5
Crenarchaeote_SCGC_AAA261_N23
Candidatus_Geoarchaeota
Archaeon_V5_archaea
Archaeon_V2_archaea
Thaumarchaeota_archaeon_CSP1_1
Nitrosopumilus_maritimus_SCM1
Thaumarchaeota_archaeon_N4
Candidatus_Nitrososphaera_gargensis_Ga9
Thaumarchaeota_archaeon_JGI_OTU_3
Thaumarchaeota_archaeon_JGI_OTU_1
Candidatus_Caldiarchaeum_subterraneum
miscellaneous_Crenarchaeota_group_15_archaeon_DG_45
Bathyarchaeota_archaeon_BA2
Candidatus_Bathyarchaeota_archaeon_B24
Candidatus_Bathyarchaeota_archaeon_B25
Candidatus_Thorarchaeota_archaeon_AB_25
Candidatus_Heimdallarchaeota_archaeon_AB_125
Candidatus_Odinarchaeota_archaeon_LCB_4
Lokiarchaeum_sp_GC14_75
Hadesarchaea_archaeon_YNP_N21
Hadesarchaea_archaeon_DG_33
candidate_divison_MSBL1_archaeon_SCGC_AAA259I09
Thermococcus_barophilus_MP
Palaeococcus_pacificus_DY20341
Arc_I_group_archaeon_B15fssc0709_Meth_Bin003
Methanotorris_formicicus_Mc_S_70
Methanothermococcus_thermolithotrophicus_DSM_2095
Methanothermus_fervidus_DSM_2088
Methanothermobacter_thermautotrophicus_str_Delta_H
Nanoarchaeum_equitans_Kin4_M
Archaeon_nanoarchaeote_Nst1
Archaeon_GW2011_AR4
Archaeon_GW2011_AR1
Candidatus_Aenigmarchaeum_subterraneum_SCGC_AAA011_O16
Archaeon_GW2011_AR5
Candidatus_Nanosalina_sp_J07AB43
Candidatus_Iainarchaeum_andersonii_SCGC_AAA011_E11
Archaeon_GW2011_AR10
Candidatus_Altiarchaeales_archaeon_WOR_SM1_SCG
Candidatus_Altiarchaeales_archaeon_WOR_SM1_86_2
Methanospirillum_hungatei_JF_1
Methanoculleus_marisnigri_JR1
Methanosarcina_thermophila_TM_1
Methanocella_paludicola_SANAE
Archaeoglobus_veneficus_SNP6
Archaeoglobus_profundus_DSM_5631
Ferroglobus_placidus_DSM_10642
Thermoplasma_volcanium_GSS1
Aciduliprofundum_boonei_T469
Methanomassiliicoccus_luminyensis_B10
Thermoplasmatales_archaeon_SG8_52
0.3

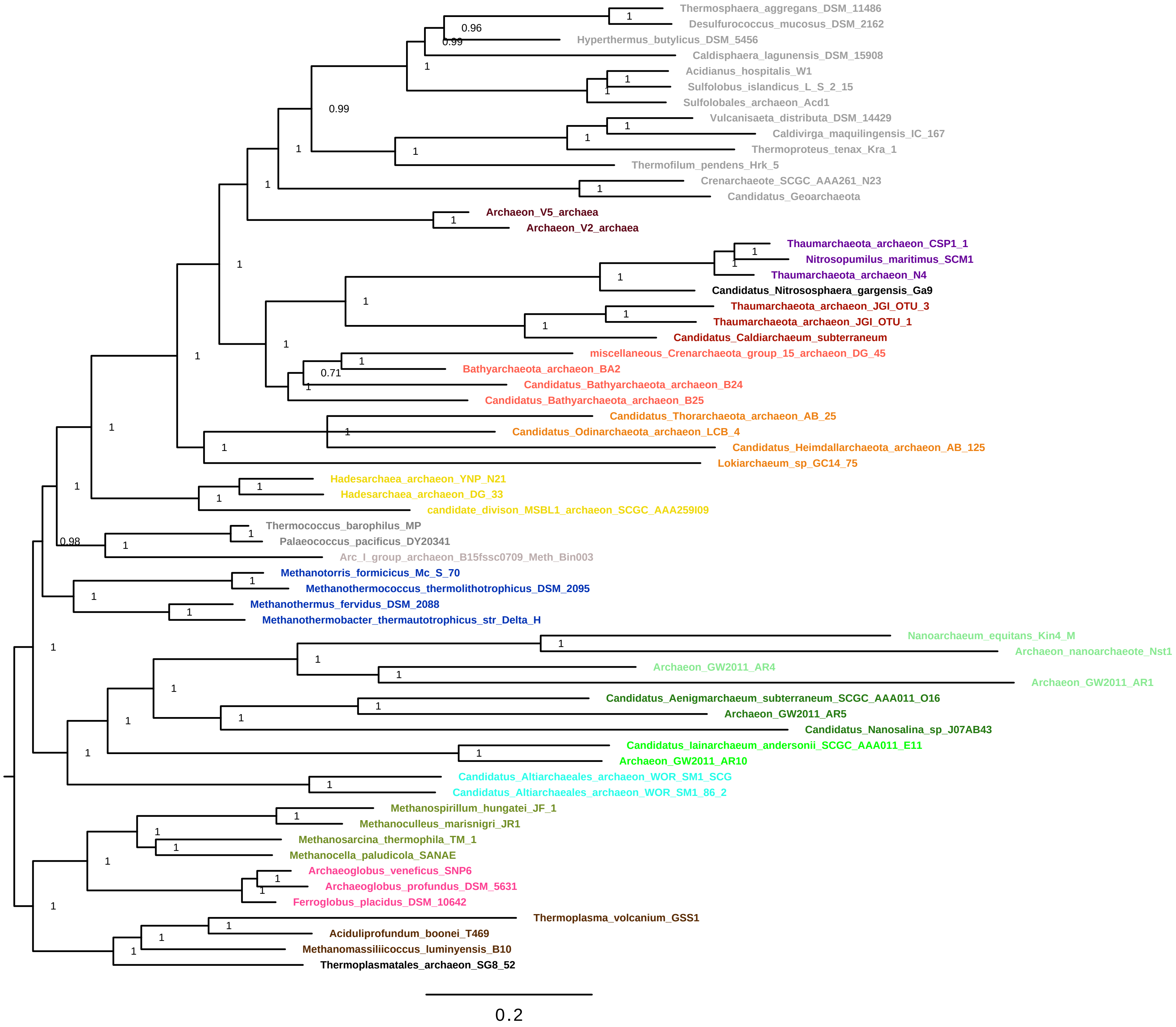

Thermosphaera_aggregans_DSM_11486
Desulfurococcus_mucosus_DSM_2162
Hyperthermus_butylicus_DSM_5456
Caldisphaera_lagunensis_DSM_15908
Acidianus_hospitalis_W1
Sulfolobus_islandicus_L_S_2_15
Sulfolobales_archaeon_Acd1
Vulcanisaeta_distributa_DSM_14429
Caldivirga_maquilingensis_IC_167
Thermoproteus_tenax_Kra_1
Thermofilum_pendens_Hrk_5
Crenarchaeote_SCGC_AAA261_N23
Candidatus_Geoarchaeota
Archaeon_V5_archaea
Archaeon_V2_archaea
Thaumarchaeota_archaeon_CSP1_1
Nitrosopumilus_maritimus_SCM1
Thaumarchaeota_archaeon_N4
Candidatus_Nitrososphaera_gargensis_Ga9
Thaumarchaeota_archaeon_JGI_OTU_3
Thaumarchaeota_archaeon_JGI_OTU_1
Candidatus_Caldiarchaeum_subterraneum
miscellaneous_Crenarchaeota_group_15_archaeon_DG_45
Bathyarchaeota_archaeon_BA2
Candidatus_Bathyarchaeota_archaeon_B24
Candidatus_Bathyarchaeota_archaeon_B25
Candidatus_Thorarchaeota_archaeon_AB_25
Candidatus_Odinarchaeota_archaeon_LCB_4
Candidatus_Heimdallarchaeota_archaeon_AB_125
Lokiarchaeum_sp_GC14_75
Hadesarchaea_archaeon_YNP_N21
Hadesarchaea_archaeon_DG_33
candidate_divison_MSBL1_archaeon_SCGC_AAA259I09
Thermococcus_barophilus_MP
Palaeococcus_pacificus_DY20341
Arc_I_group_archaeon_B15fssc0709_Meth_Bin003
Methanotorris_formicicus_Mc_S_70
Methanothermococcus_thermolithotrophicus_DSM_2095
Methanothermus_fervidus_DSM_2088
Methanothermobacter_thermautotrophicus_str_Delta_H
Nanoarchaeum_equitans_Kin4_M
Archaeon_nanoarchaeote_Nst1
Archaeon_GW2011_AR4
Archaeon_GW2011_AR1
Candidatus_Aenigmarchaeum_subterraneum_SCGC_AAA011_O16
Archaeon_GW2011_AR5
Candidatus_Nanosalina_sp_J07AB43
Candidatus_Iainarchaeum_andersonii_SCGC_AAA011_E11
Archaeon_GW2011_AR10
Candidatus_Altiarchaeales_archaeon_WOR_SM1_SCG
Candidatus_Altiarchaeales_archaeon_WOR_SM1_86_2
Methanospirillum_hungatei_JF_1
Methanoculleus_marisnigri_JR1
Methanosarcina_thermophila_TM_1
Methanocella_paludicola_SANAE
Archaeoglobus_veneficus_SNP6
Archaeoglobus_profundus_DSM_5631
Ferroglobus_placidus_DSM_10642
Thermoplasma_volcanium_GSS1
Aciduliprofundum_boonei_T469
Methanomassiliicoccus_luminyensis_B10
Thermoplasmatales_archaeon_SG8_52
0.2

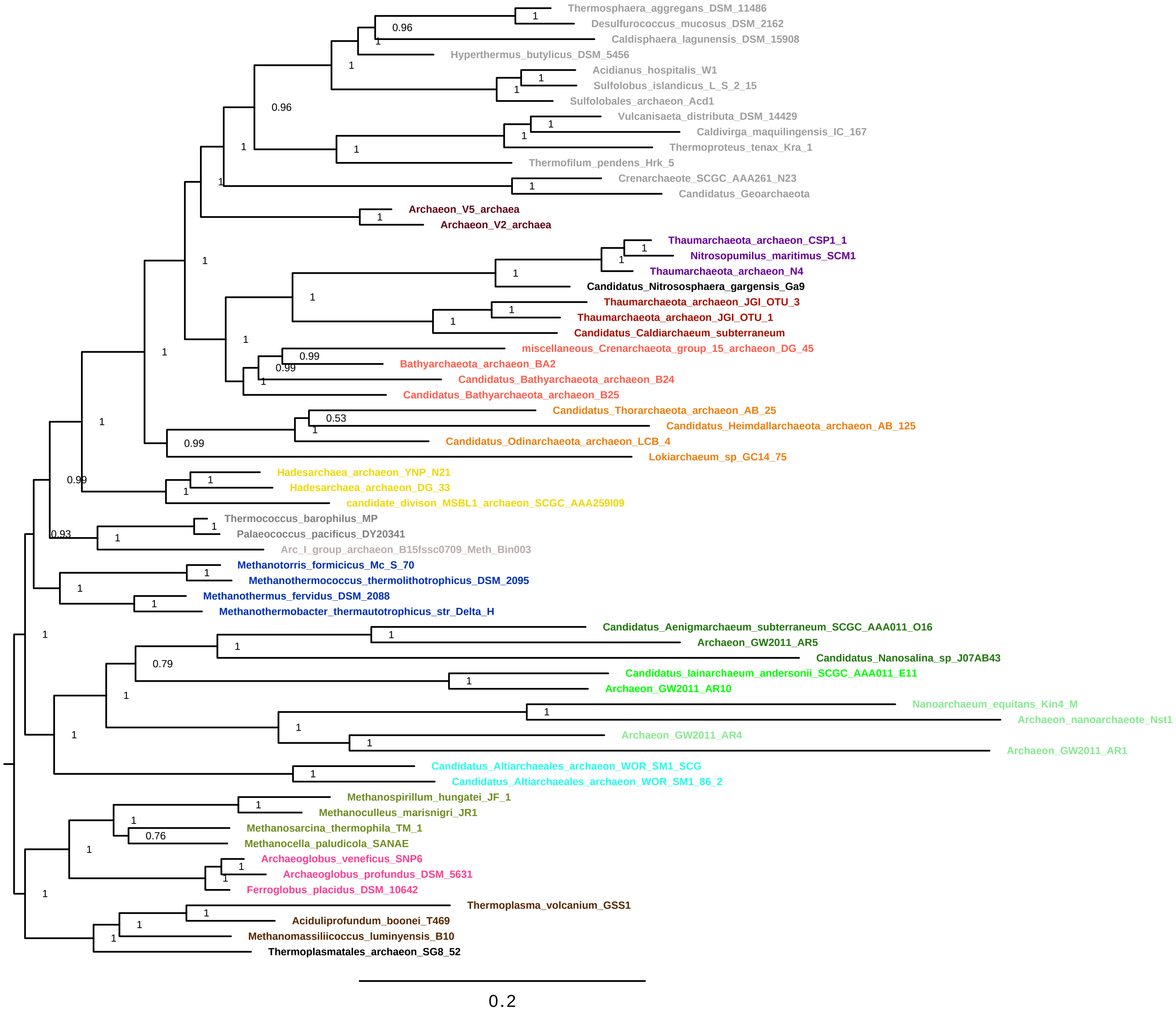
Thermosphaera_aggregans_DSM_11486
Desulfurococcus_mucosus_DSM_2162
Caldisphaera_lagunensis_DSM_15908
Hyperthermus_butylicus_DSM_5456
Acidianus_hospitalis_W1
Sulfolobus_islandicus_L_S_2_15
Sulfolobales_archaeon_Acd1
Vulcanisaeta_distributa_DSM_14429
Caldivirga_maquilingensis_IC_167
Thermoproteus_tenax_Kra_1
Thermofilum_pendens_Hrk_5
Crenarchaeote_SCGC_AAA261_N23
Candidatus_Geoarchaeota
Archaeon_V5_archaea
Archaeon_V2_archaea
Thaumarchaeota_archaeon_CSP1_1
Nitrosopumilus_maritimus_SCM1
Thaumarchaeota_archaeon_N4
Candidatus_Nitrososphaera_gargensis_Ga9
Thaumarchaeota_archaeon_JGI_OTU_3
Thaumarchaeota_archaeon_JGI_OTU_1
Candidatus_Caldiarchaeum_subterraneum
miscellaneous_Crenarchaeota_group_15_archaeon_DG_45
Bathyarchaeota_archaeon_BA2
Candidatus_Bathyarchaeota_archaeon_B24
Candidatus_Bathyarchaeota_archaeon_B25
Candidatus_Thorarchaeota_archaeon_AB_25
Candidatus_Heimdallarchaeota_archaeon_AB_125
Candidatus_Odinarchaeota_archaeon_LCB_4
Lokiarchaeum_sp_GC14_75
Hadesarchaea_archaeon_YNP_N21
Hadesarchaea_archaeon_DG_33
candidate_divison_MSBL1_archaeon_SCGC_AAA259I09
Thermococcus_barophilus_MP
Palaeococcus_pacificus_DY20341
Arc_I_group_archaeon_B15fssc0709_Meth_Bin003
Methanotorris_formicicus_Mc_S_70
Methanothermococcus_thermolithotrophicus_DSM_2095
Methanothermus_fervidus_DSM_2088
Methanothermobacter_thermautotrophicus_str_Delta_H
Candidatus_Aenigmarchaeum_subterraneum_SCGC_AAA011_O16
Archaeon_GW2011_AR5
Candidatus_Nanosalina_sp_J07AB43
Candidatus_Iainarchaeum_andersonii_SCGC_AAA011_E11
Archaeon_GW2011_AR10
Nanoarchaeum_equitans_Kin4_M
Archaeon_nanoarchaeote_Nst1
Archaeon_GW2011_AR4
Archaeon_GW2011_AR1
Candidatus_Altiarchaeales_archaeon_WOR_SM1_SCG
Candidatus_Altiarchaeales_archaeon_WOR_SM1_86_2
Methanospirillum_hungatei_JF_1
Methanoculleus_marisnigri_JR1
Methanosarcina_thermophila_TM_1
Methanocella_paludicola_SANAE
Archaeoglobus_veneficus_SNP6
Archaeoglobus_profundus_DSM_5631
Ferroglobus_placidus_DSM_10642
Thermoplasma_volcanium_GSS1
Aciduliprofundum_boonei_T469
Methanomassiliicoccus_luminyensis_B10
Thermoplasmatales_archaeon_SG8_52
0.2

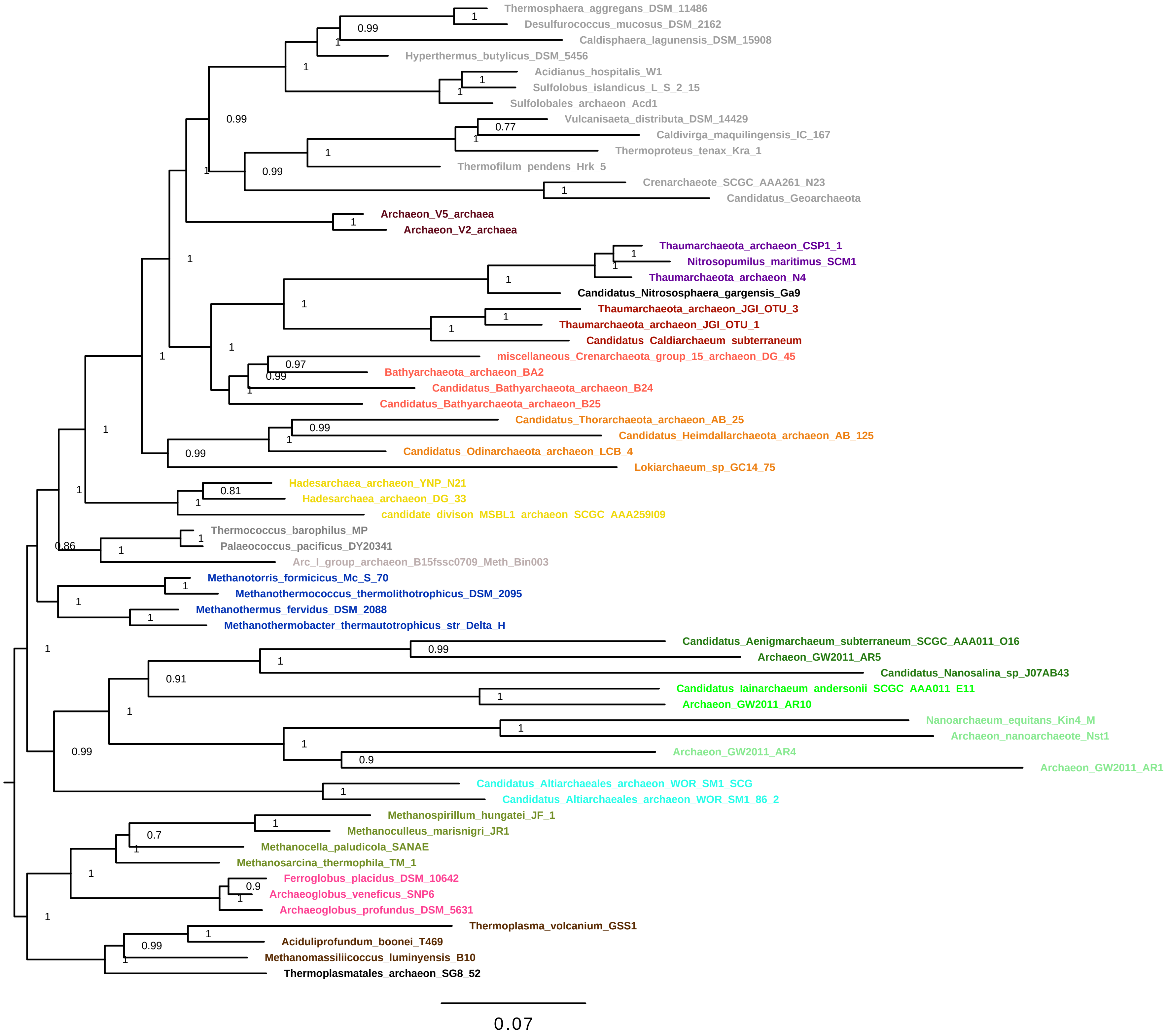
Thermosphaera_aggregans_DSM_11486
Desulfurococcus_mucosus_DSM_2162
Caldisphaera_lagunensis_DSM_15908
Hyperthermus_butylicus_DSM_5456
Acidianus_hospitalis_W1
Sulfolobus_islandicus_L_S_2_15
Sulfolobales_archaeon_Acd1
Vulcanisaeta_distributa_DSM_14429
Caldivirga_maquilingensis_IC_167
Thermoproteus_tenax_Kra_1
Thermofilum_pendens_Hrk_5
Crenarchaeote_SCGC_AAA261_N23
Candidatus_Geoarchaeota
Archaeon_V5_archaea
Archaeon_V2_archaea
Thaumarchaeota_archaeon_CSP1_1
Nitrosopumilus_maritimus_SCM1
Thaumarchaeota_archaeon_N4
Candidatus_Nitrososphaera_gargensis_Ga9
Thaumarchaeota_archaeon_JGI_OTU_3
Thaumarchaeota_archaeon_JGI_OTU_1
Candidatus_Caldiarchaeum_subterraneum
miscellaneous_Crenarchaeota_group_15_archaeon_DG_45
Bathyarchaeota_archaeon_BA2
Candidatus_Bathyarchaeota_archaeon_B24
Candidatus_Bathyarchaeota_archaeon_B25
Candidatus_Thorarchaeota_archaeon_AB_25
Candidatus_Heimdallarchaeota_archaeon_AB_125
Candidatus_Odinarchaeota_archaeon_LCB_4
Lokiarchaeum_sp_GC14_75
Hadesarchaea_archaeon_YNP_N21
Hadesarchaea_archaeon_DG_33
candidate_divison_MSBL1_archaeon_SCGC_AAA259I09
Thermococcus_barophilus_MP
Palaeococcus_pacificus_DY20341
Arc_I_group_archaeon_B15fssc0709_Meth_Bin003
Methanotorris_formicicus_Mc_S_70
Methanothermococcus_thermolithotrophicus_DSM_2095
Methanothermus_fervidus_DSM_2088
Methanothermobacter_thermautotrophicus_str_Delta_H
Candidatus_Aenigmarchaeum_subterraneum_SCGC_AAA011_O16
Archaeon_GW2011_AR5
Candidatus_Nanosalina_sp_J07AB43
Candidatus_Iainarchaeum_andersonii_SCGC_AAA011_E11
Archaeon_GW2011_AR10
Nanoarchaeum_equitans_Kin4_M
Archaeon_nanoarchaeote_Nst1
Archaeon_GW2011_AR4
Archaeon_GW2011_AR1
Candidatus_Altiarchaeales_archaeon_WOR_SM1_SCG
Candidatus_Altiarchaeales_archaeon_WOR_SM1_86_2
Methanospirillum_hungatei_JF_1
Methanoculleus_marisnigri_JR1
Methanocella_paludicola_SANAE
Methanosarcina_thermophila_TM_1
Ferroglobus_placidus_DSM_10642
Archaeoglobus_veneficus_SNP6
Archaeoglobus_profundus_DSM_5631
Thermoplasma_volcanium_GSS1
Aciduliprofundum_boonei_T469
Methanomassiliicoccus_luminyensis_B10
Thermoplasmatales_archaeon_SG8_52
0.07

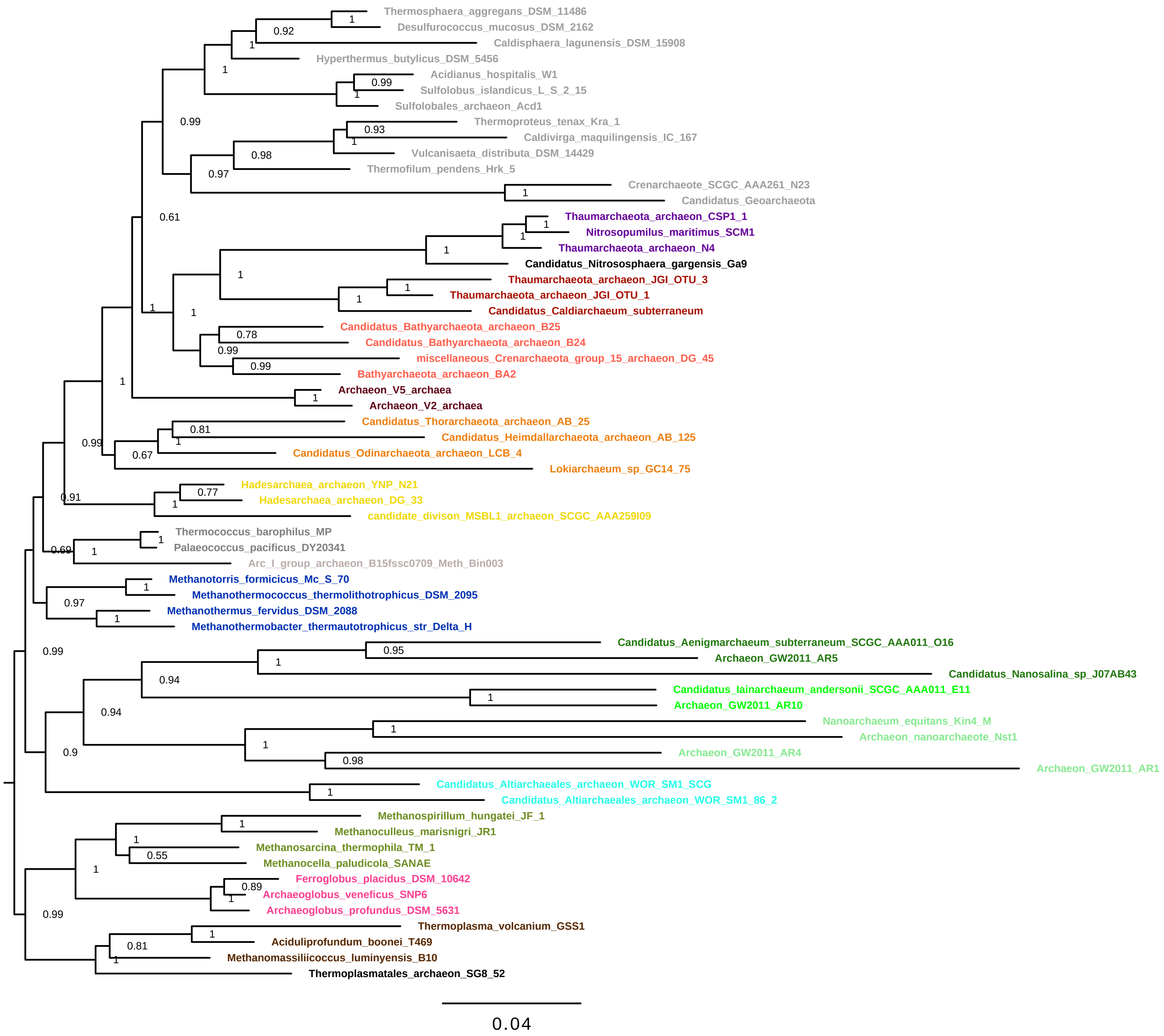
Thermosphaera_aggregans_DSM_11486
Desulfurococcus_mucosus_DSM_2162
Caldisphaera_lagunensis_DSM_15908
Hyperthermus_butylicus_DSM_5456
Acidianus_hospitalis_W1
Sulfolobus_islandicus_L_S_2_15
Sulfolobales_archaeon_Acd1
Thermoproteus_tenax_Kra_1
Caldivirga_maquilingensis_IC_167
Vulcanisaeta_distributa_DSM_14429
Thermofilum_pendens_Hrk_5
Crenarchaeote_SCGC_AAA261_N23
Candidatus_Geoarchaeota
Thaumarchaeota_archaeon_CSP1_1
Nitrosopumilus_maritimus_SCM1
Thaumarchaeota_archaeon_N4
Candidatus_Nitrososphaera_gargensis_Ga9
Thaumarchaeota_archaeon_JGI_OTU_3
Thaumarchaeota_archaeon_JGI_OTU_1
Candidatus_Caldiarchaeum_subterraneum
Candidatus_Bathyarchaeota_archaeon_B25
Candidatus_Bathyarchaeota_archaeon_B24
miscellaneous_Crenarchaeota_group_15_archaeon_DG_45
Bathyarchaeota_archaeon_BA2
Archaeon_V5_archaea
Archaeon_V2_archaea
Candidatus_Thorarchaeota_archaeon_AB_25
Candidatus_Heimdallarchaeota_archaeon_AB_125
Candidatus_Odinarchaeota_archaeon_LCB_4
Lokiarchaeum_sp_GC14_75
Hadesarchaea_archaeon_YNP_N21
Hadesarchaea_archaeon_DG_33
candidate_divison_MSBL1_archaeon_SCGC_AAA259I09
Thermococcus_barophilus_MP
Palaeococcus_pacificus_DY20341
Arc_I_group_archaeon_B15fssc0709_Meth_Bin003
Methanotorris_formicicus_Mc_S_70
Methanothermococcus_thermolithotrophicus_DSM_2095
Methanothermus_fervidus_DSM_2088
Methanothermobacter_thermautotrophicus_str_Delta_H
Candidatus_Aenigmarchaeum_subterraneum_SCGC_AAA011_O16
Archaeon_GW2011_AR5
Candidatus_Nanosalina_sp_J07AB43
Candidatus_Iainarchaeum_andersonii_SCGC_AAA011_E11
Archaeon_GW2011_AR10
Nanoarchaeum_equitans_Kin4_M
Archaeon_nanoarchaeote_Nst1
Archaeon_GW2011_AR4
Archaeon_GW2011_AR1
Candidatus_Altiarchaeales_archaeon_WOR_SM1_SCG
Candidatus_Altiarchaeales_archaeon_WOR_SM1_86_2
Methanospirillum_hungatei_JF_1
Methanoculleus_marisnigri_JR1
Methanosarcina_thermophila_TM_1
Methanocella_paludicola_SANAE
Ferroglobus_placidus_DSM_10642
Archaeoglobus_veneficus_SNP6
Archaeoglobus_profundus_DSM_5631
Thermoplasma_volcanium_GSS1
Aciduliprofundum_boonei_T469
Methanomassiliicoccus_luminyensis_B10
Thermoplasmatales_archaeon_SG8_52
0.04

Supplementary Figure S4. Unrooted Bayesian phylogeny of *Archaea* inferred through the Slow-Fast procedure by excluding the DPANN.

Starting from the A supermatrix (72 protein families, 199 taxa, 15,430 amino acids positions), the fastest evolving sites were removed progressively. At each step, a Bayesian tree was inferred with PHYLOBAYES using the CAT+GTR+G4 model. Values at branch correspond to posterior probabilities. The scale bars indicate the average number of substitutions per site.

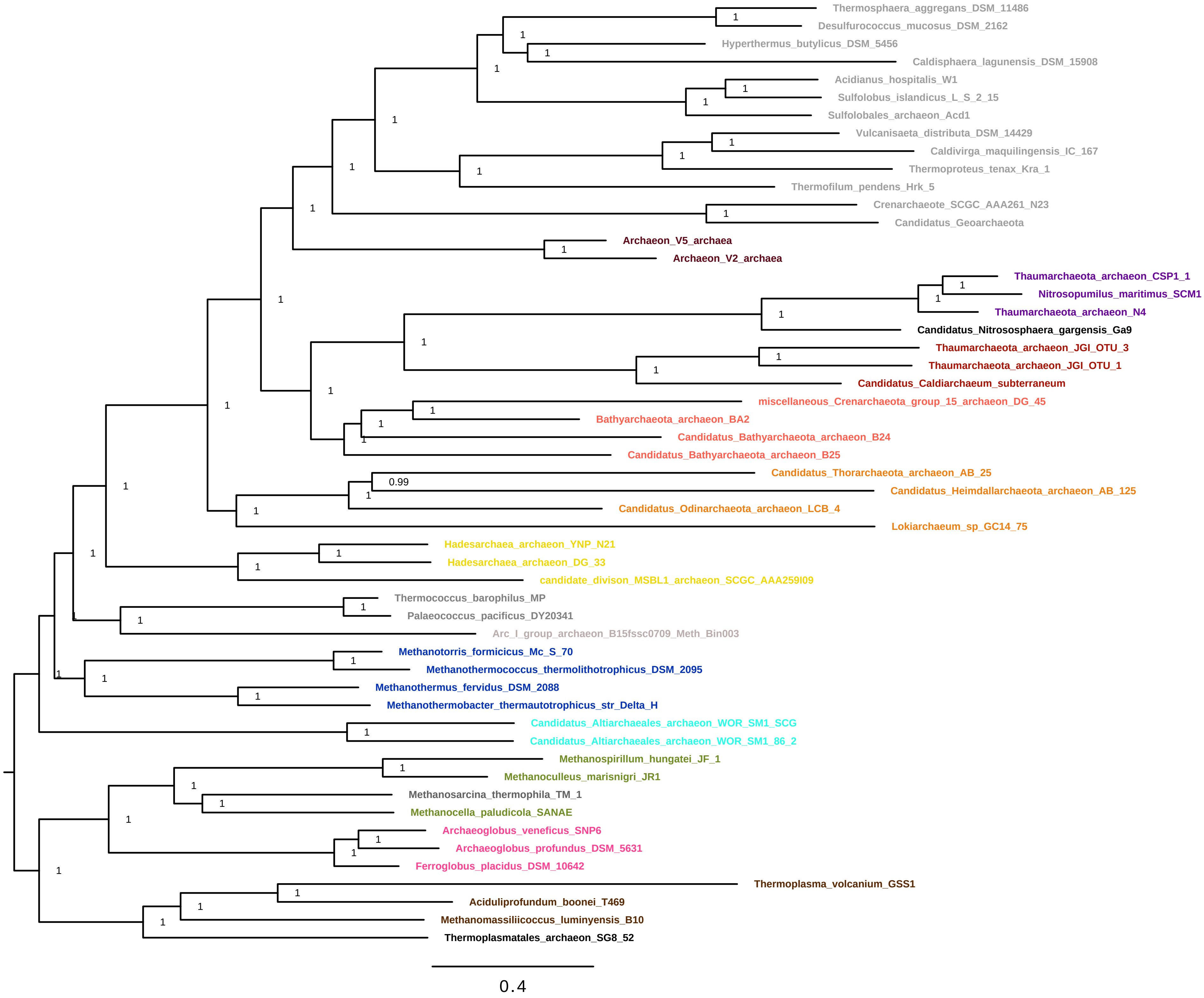
Thermosphaera_aggregans_DSM_11486
Desulfurococcus_mucosus_DSM_2162
Hyperthermus_butylicus_DSM_5456
Caldisphaera_lagunensis_DSM_15908
Acidianus_hospitalis_W1
Sulfolobus_islandicus_L_S_2_15
Sulfolobales_archaeon_Acd1
Vulcanisaeta_distributa_DSM_14429
Caldivirga_maquilingensis_IC_167
Thermoproteus_tenax_Kra_1
Thermofilum_pendens_Hrk_5
Crenarchaeote_SCGC_AAA261_N23
Candidatus_Geoarchaeota
Archaeon_V5_archaea
Archaeon_V2_archaea
Thaumarchaeota_archaeon_CSP1_1
Nitrosopumilus_maritimus_SCM1
Thaumarchaeota_archaeon_N4
Candidatus_Nitrososphaera_gargensis_Ga9
Thaumarchaeota_archaeon_JGI_OTU_3
Thaumarchaeota_archaeon_JGI_OTU_1
Candidatus_Caldiarchaeum_subterraneum
miscellaneous_Crenarchaeota_group_15_archaeon_DG_45
Bathyarchaeota_archaeon_BA2
Candidatus_Bathyarchaeota_archaeon_B24
Candidatus_Bathyarchaeota_archaeon_B25
Candidatus_Thorarchaeota_archaeon_AB_25
Candidatus_Heimdallarchaeota_archaeon_AB_125
Candidatus_Odinarchaeota_archaeon_LCB_4
Lokiarchaeum_sp_GC14_75
Hadesarchaea_archaeon_YNP_N21
Hadesarchaea_archaeon_DG_33
candidate_divison_MSBL1_archaeon_SCGC_AAA259I09
Thermococcus_barophilus_MP
Palaeococcus_pacificus_DY20341
Arc_I_group_archaeon_B15fssc0709_Meth_Bin003
Methanotorris_formicicus_Mc_S_70
Methanothermococcus_thermolithotrophicus_DSM_2095
Methanothermus_fervidus_DSM_2088
Methanothermobacter_thermautotrophicus_str_Delta_H
Candidatus_Altiarchaeales_archaeon_WOR_SM1_SCG
Candidatus_Altiarchaeales_archaeon_WOR_SM1_86_2
Methanospirillum_hungatei_JF_1
Methanoculleus_marisnigri_JR1
Methanosarcina_thermophila_TM_1
Methanocella_paludicola_SANAE
Archaeoglobus_veneficus_SNP6
Archaeoglobus_profundus_DSM_5631
Ferroglobus_placidus_DSM_10642
Thermoplasma_volcanium_GSS1
Aciduliprofundum_boonei_T469
Methanomassiliicoccus_luminyensis_B10
Thermoplasmatales_archaeon_SG8_52
0.99
0.4

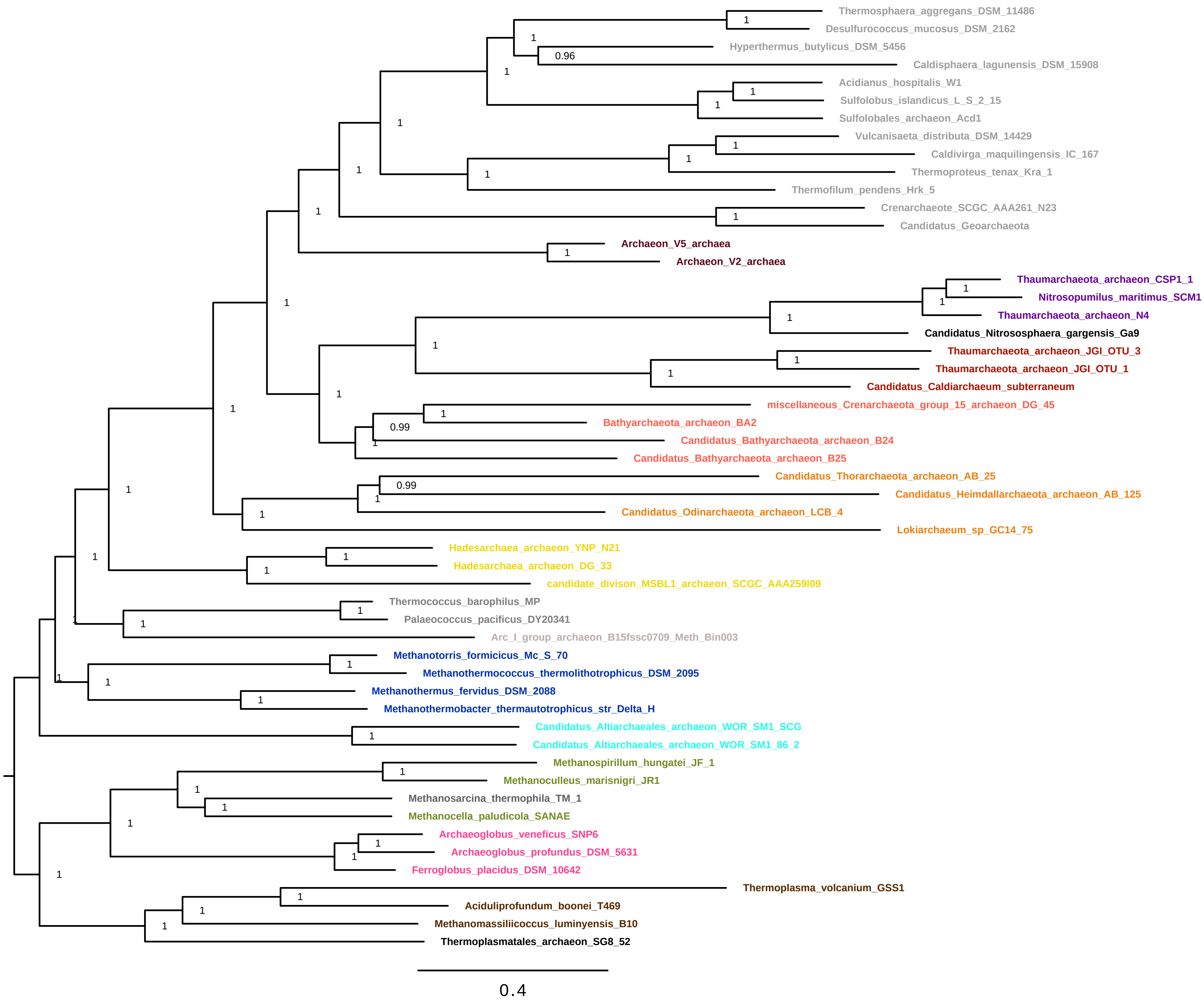

Thermosphaera_aggregans_DSM_11486
Desulfurococcus_mucosus_DSM_2162
Hyperthermus_butylicus_DSM_5456
Caldisphaera_lagunensis_DSM_15908
Acidianus_hospitalis_W1
Sulfolobus_islandicus_L_S_2_15
Sulfolobales_archaeon_Acd1
Vulcanisaeta_distributa_DSM_14429
Caldivirga_maquilingensis_IC_167
Thermoproteus_tenax_Kra_1
Thermofilum_pendens_Hrk_5
Crenarchaeote_SCGC_AAA261_N23
Candidatus_Geoarchaeota
Archaeon_V5_archaea
Archaeon_V2_archaea
Thaumarchaeota_archaeon_CSP1_1
Nitrosopumilus_maritimus_SCM1
Thaumarchaeota_archaeon_N4
Candidatus_Nitrososphaera_gargensis_Ga9
Thaumarchaeota_archaeon_JGI_OTU_3
Thaumarchaeota_archaeon_JGI_OTU_1
Candidatus_Caldiarchaeum_subterraneum
miscellaneous_Crenarchaeota_group_15_archaeon_DG_45
Bathyarchaeota_archaeon_BA2
Candidatus_Bathyarchaeota_archaeon_B24
Candidatus_Bathyarchaeota_archaeon_B25
Candidatus_Thorarchaeota_archaeon_AB_25
Candidatus_Heimdallarchaeota_archaeon_AB_125
Candidatus_Odinarchaeota_archaeon_LCB_4
Lokiarchaeum_sp_GC14_75
Hadesarchaea_archaeon_YNP_N21
Hadesarchaea_archaeon_DG_33
candidate_divison_MSBL1_archaeon_SCGC_AAA259I09
Thermococcus_barophilus_MP
Palaeococcus_pacificus_DY20341
Arc_I_group_archaeon_B15fssc0709_Meth_Bin003
Methanotorris_formicicus_Mc_S_70
Methanothermococcus_thermolithotrophicus_DSM_2095
Methanothermus_fervidus_DSM_2088
Methanothermobacter_thermautotrophicus_str_Delta_H
Candidatus_Altiarchaeales_archaeon_WOR_SM1_SCG
Candidatus_Altiarchaeales_archaeon_WOR_SM1_86_2
Methanospirillum_hungatei_JF_1
Methanoculleus_marisnigri_JR1
Methanosarcina_thermophila_TM_1
Methanocella_paludicola_SANAE
Archaeoglobus_veneficus_SNP6
Archaeoglobus_profundus_DSM_5631
Ferroglobus_placidus_DSM_10642
Thermoplasma_volcanium_GSS1
Aciduliprofundum_boonei_T469
Methanomassiliicoccus_luminyensis_B10
Thermoplasmatales_archaeon_SG8_52
0.96
0.99
0.99
0.4

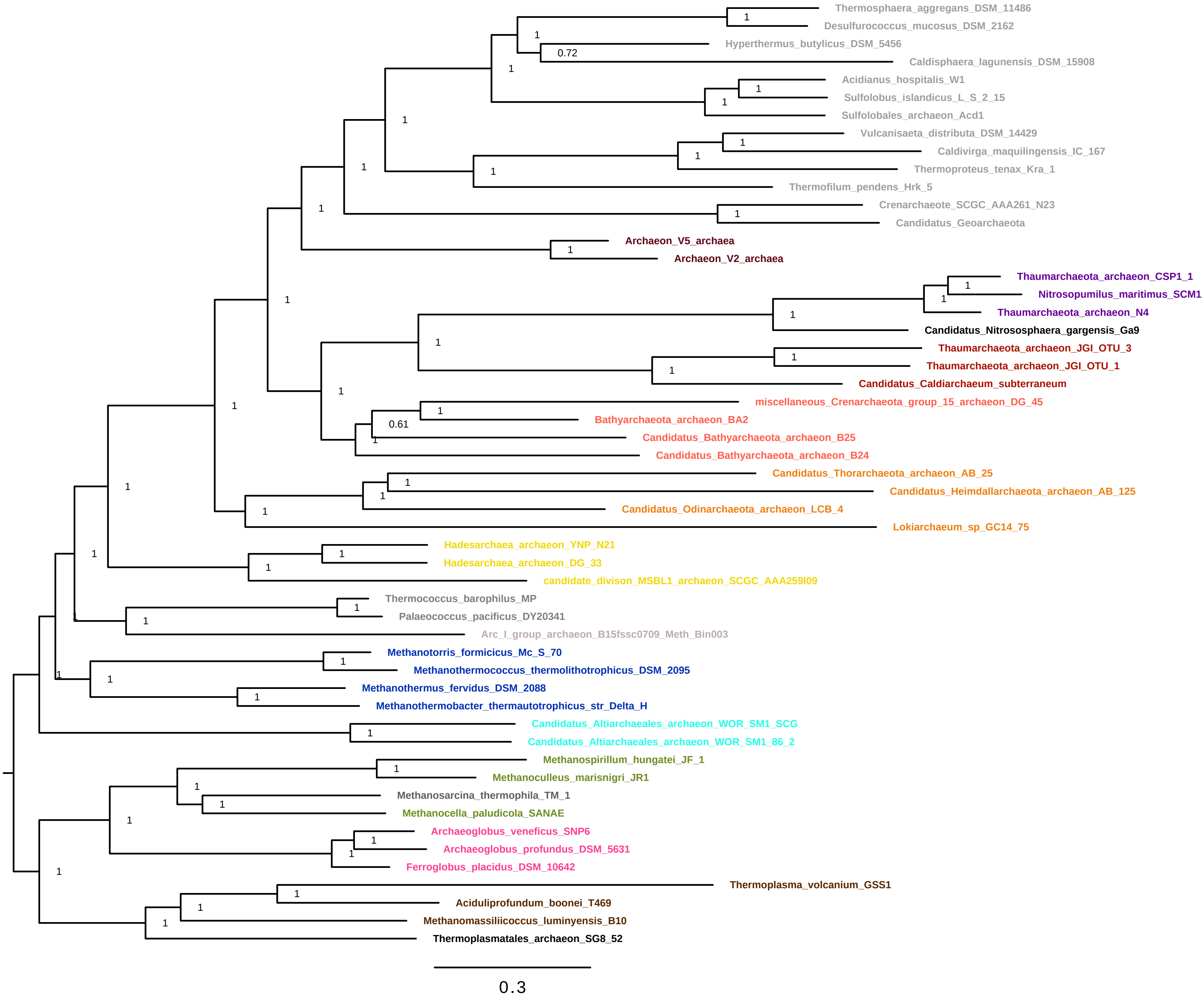

Thermosphaera_aggregans_DSM_11486
Desulfurococcus_mucosus_DSM_2162
Hyperthermus_butylicus_DSM_5456
Caldisphaera_lagunensis_DSM_15908
Acidianus_hospitalis_W1
Sulfolobus_islandicus_L_S_2_15
Sulfolobales_archaeon_Acd1
Vulcanisaeta_distributa_DSM_14429
Caldivirga_maquilingensis_IC_167
Thermoproteus_tenax_Kra_1
Thermofilum_pendens_Hrk_5
Crenarchaeote_SCGC_AAA261_N23
Candidatus_Geoarchaeota
Archaeon_V5_archaea
Archaeon_V2_archaea
Thaumarchaeota_archaeon_CSP1_1
Nitrosopumilus_maritimus_SCM1
Thaumarchaeota_archaeon_N4
Candidatus_Nitrososphaera_gargensis_Ga9
Thaumarchaeota_archaeon_JGI_OTU_3
Thaumarchaeota_archaeon_JGI_OTU_1
Candidatus_Caldiarchaeum_subterraneum
miscellaneous_Crenarchaeota_group_15_archaeon_DG_45
Bathyarchaeota_archaeon_BA2
Candidatus_Bathyarchaeota_archaeon_B25
Candidatus_Bathyarchaeota_archaeon_B24
Candidatus_Thorarchaeota_archaeon_AB_25
Candidatus_Heimdallarchaeota_archaeon_AB_125
Candidatus_Odinarchaeota_archaeon_LCB_4
Lokiarchaeum_sp_GC14_75
Hadesarchaea_archaeon_YNP_N21
Hadesarchaea_archaeon_DG_33
candidate_divison_MSBL1_archaeon_SCGC_AAA259I09
Thermococcus_barophilus_MP
Palaeococcus_pacificus_DY20341
Arc_I_group_archaeon_B15fssc0709_Meth_Bin003
Methanotorris_formicicus_Mc_S_70
Methanothermococcus_thermolithotrophicus_DSM_2095
Methanothermus_fervidus_DSM_2088
Methanothermobacter_thermautotrophicus_str_Delta_H
Candidatus_Altiarchaeales_archaeon_WOR_SM1_SCG
Candidatus_Altiarchaeales_archaeon_WOR_SM1_86_2
Methanospirillum_hungatei_JF_1
Methanoculleus_marisnigri_JR1
Methanosarcina_thermophila_TM_1
Methanocella_paludicola_SANAE
Archaeoglobus_veneficus_SNP6
Archaeoglobus_profundus_DSM_5631
Ferroglobus_placidus_DSM_10642
Thermoplasma_volcanium_GSS1
Aciduliprofundum_boonei_T469
Methanomassiliicoccus_luminyensis_B10
Thermoplasmatales_archaeon_SG8_52
0.72
0.61
0.3

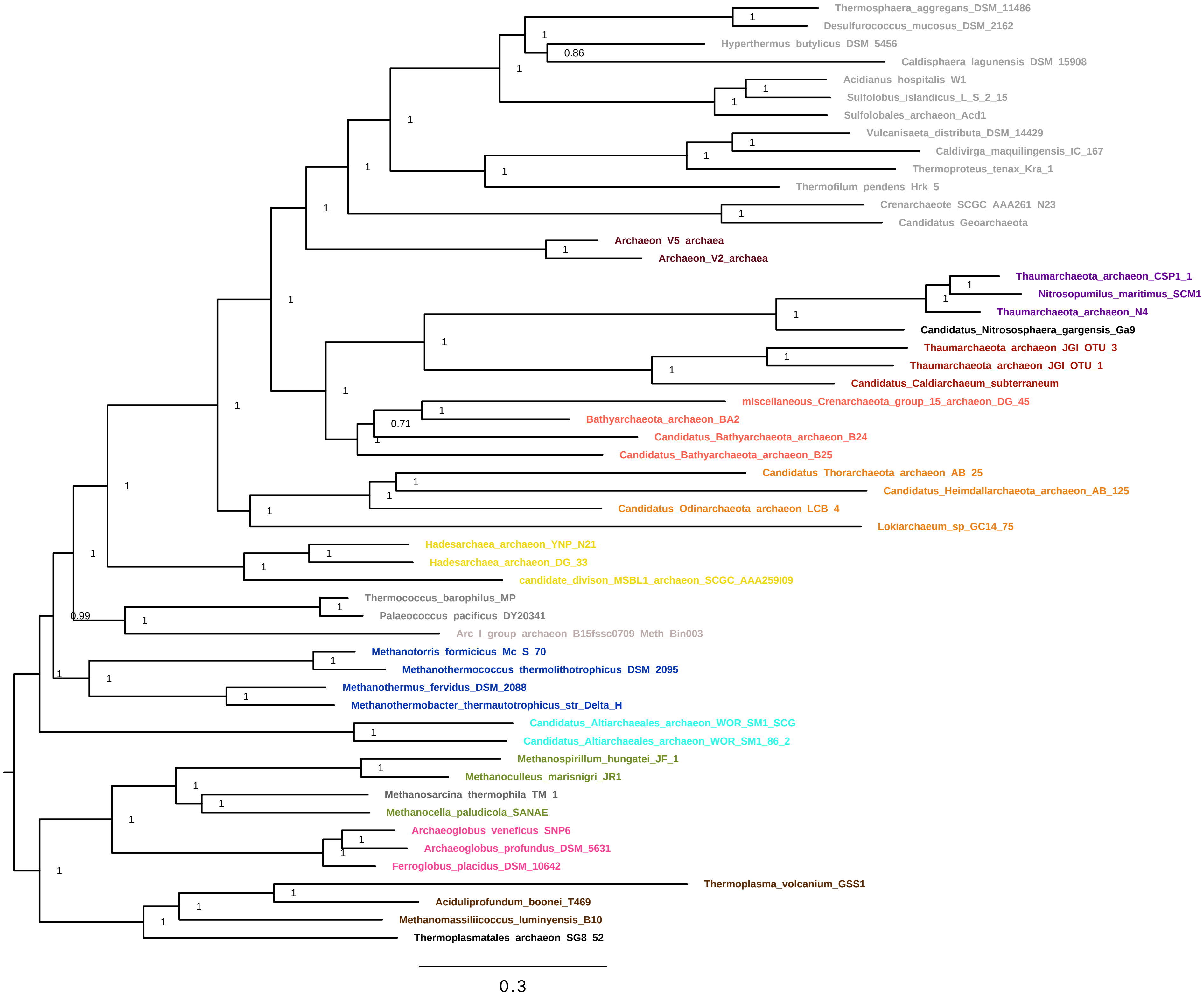
Thermosphaera_aggregans_DSM_11486
Desulfurococcus_mucosus_DSM_2162
Hyperthermus_butylicus_DSM_5456
Caldisphaera_lagunensis_DSM_15908
Acidianus_hospitalis_W1
Sulfolobus_islandicus_L_S_2_15
Sulfolobales_archaeon_Acd1
Vulcanisaeta_distributa_DSM_14429
Caldivirga_maquilingensis_IC_167
Thermoproteus_tenax_Kra_1
Thermofilum_pendens_Hrk_5
Crenarchaeote_SCGC_AAA261_N23
Candidatus_Geoarchaeota
Archaeon_V5_archaea
Archaeon_V2_archaea
Thaumarchaeota_archaeon_CSP1_1
Nitrosopumilus_maritimus_SCM1
Thaumarchaeota_archaeon_N4
Candidatus_Nitrososphaera_gargensis_Ga9
Thaumarchaeota_archaeon_JGI_OTU_3
Thaumarchaeota_archaeon_JGI_OTU_1
Candidatus_Caldiarchaeum_subterraneum
miscellaneous_Crenarchaeota_group_15_archaeon_DG_45
Bathyarchaeota_archaeon_BA2
Candidatus_Bathyarchaeota_archaeon_B24
Candidatus_Bathyarchaeota_archaeon_B25
Candidatus_Thorarchaeota_archaeon_AB_25
Candidatus_Heimdallarchaeota_archaeon_AB_125
Candidatus_Odinarchaeota_archaeon_LCB_4
Lokiarchaeum_sp_GC14_75
Hadesarchaea_archaeon_YNP_N21
Hadesarchaea_archaeon_DG_33
candidate_divison_MSBL1_archaeon_SCGC_AAA259I09
Thermococcus_barophilus_MP
Palaeococcus_pacificus_DY20341
Arc_I_group_archaeon_B15fssc0709_Meth_Bin003
Methanotorris_formicicus_Mc_S_70
Methanothermococcus_thermolithotrophicus_DSM_2095
Methanothermus_fervidus_DSM_2088
Methanothermobacter_thermautotrophicus_str_Delta_H
Candidatus_Altiarchaeales_archaeon_WOR_SM1_SCG
Candidatus_Altiarchaeales_archaeon_WOR_SM1_86_2
Methanospirillum_hungatei_JF_1
Methanoculleus_marisnigri_JR1
Methanosarcina_thermophila_TM_1
Methanocella_paludicola_SANAE
Archaeoglobus_veneficus_SNP6
Archaeoglobus_profundus_DSM_5631
Ferroglobus_placidus_DSM_10642
Thermoplasma_volcanium_GSS1
Aciduliprofundum_boonei_T469
Methanomassiliicoccus_luminyensis_B10
Thermoplasmatales_archaeon_SG8_52
0.86
0.71
0.99
1
0.3

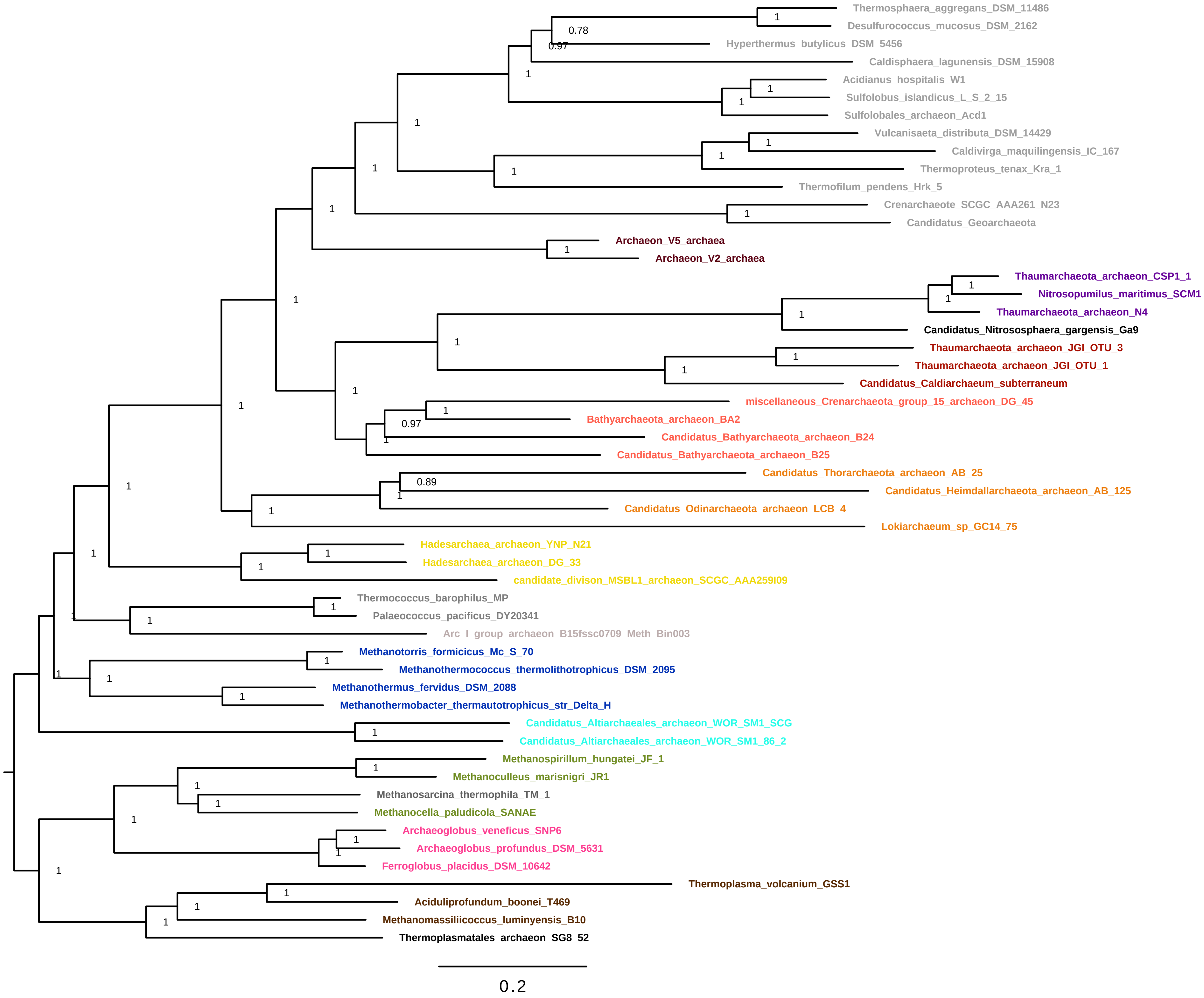
Thermosphaera_aggregans_DSM_11486
Desulfurococcus_mucosus_DSM_2162
Hyperthermus_butylicus_DSM_5456
Caldisphaera_lagunensis_DSM_15908
Acidianus_hospitalis_W1
Sulfolobus_islandicus_L_S_2_15
Sulfolobales_archaeon_Acd1
Vulcanisaeta_distributa_DSM_14429
Caldivirga_maquilingensis_IC_167
Thermoproteus_tenax_Kra_1
Thermofilum_pendens_Hrk_5
Crenarchaeote_SCGC_AAA261_N23
Candidatus_Geoarchaeota
Archaeon_V5_archaea
Archaeon_V2_archaea
Thaumarchaeota_archaeon_CSP1_1
Nitrosopumilus_maritimus_SCM1
Thaumarchaeota_archaeon_N4
Candidatus_Nitrososphaera_gargensis_Ga9
Thaumarchaeota_archaeon_JGI_OTU_3
Thaumarchaeota_archaeon_JGI_OTU_1
Candidatus_Caldiarchaeum_subterraneum
miscellaneous_Crenarchaeota_group_15_archaeon_DG_45
Bathyarchaeota_archaeon_BA2
Candidatus_Bathyarchaeota_archaeon_B24
Candidatus_Bathyarchaeota_archaeon_B25
Candidatus_Thorarchaeota_archaeon_AB_25
Candidatus_Heimdallarchaeota_archaeon_AB_125
Candidatus_Odinarchaeota_archaeon_LCB_4
Lokiarchaeum_sp_GC14_75
Hadesarchaea_archaeon_YNP_N21
Hadesarchaea_archaeon_DG_33
candidate_divison_MSBL1_archaeon_SCGC_AAA259I09
Thermococcus_barophilus_MP
Palaeococcus_pacificus_DY20341
Arc_I_group_archaeon_B15fssc0709_Meth_Bin003
Methanotorris_formicicus_Mc_S_70
Methanothermococcus_thermolithotrophicus_DSM_2095
Methanothermus_fervidus_DSM_2088
Methanothermobacter_thermautotrophicus_str_Delta_H
Candidatus_Altiarchaeales_archaeon_WOR_SM1_SCG
Candidatus_Altiarchaeales_archaeon_WOR_SM1_86_2
Methanospirillum_hungatei_JF_1
Methanoculleus_marisnigri_JR1
Methanosarcina_thermophila_TM_1
Methanocella_paludicola_SANAE
Archaeoglobus_veneficus_SNP6
Archaeoglobus_profundus_DSM_5631
Ferroglobus_placidus_DSM_10642
Thermoplasma_volcanium_GSS1
Aciduliprofundum_boonei_T469
Methanomassiliicoccus_luminyensis_B10
Thermoplasmatales_archaeon_SG8_52
0.78
0.97
0.89
0.2

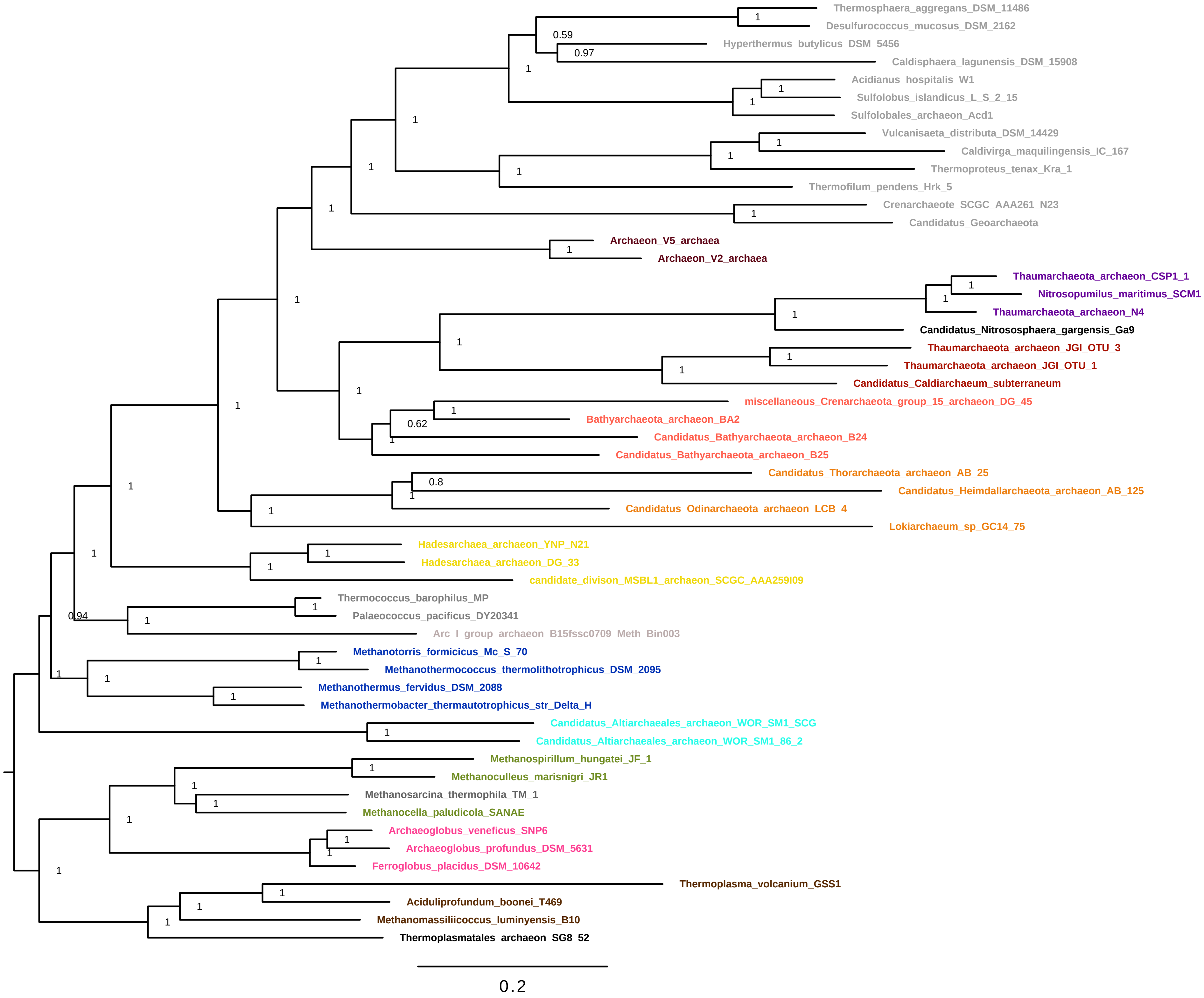
Thermosphaera_aggregans_DSM_11486
Desulfurococcus_mucosus_DSM_2162
Hyperthermus_butylicus_DSM_5456
Caldisphaera_lagunensis_DSM_15908
Acidianus_hospitalis_W1
Sulfolobus_islandicus_L_S_2_15
Sulfolobales_archaeon_Acd1
Vulcanisaeta_distributa_DSM_14429
Caldivirga_maquilingensis_IC_167
Thermoproteus_tenax_Kra_1
Thermofilum_pendens_Hrk_5
Crenarchaeote_SCGC_AAA261_N23
Candidatus_Geoarchaeota
Archaeon_V5_archaea
Archaeon_V2_archaea
Thaumarchaeota_archaeon_CSP1_1
Nitrosopumilus_maritimus_SCM1
Thaumarchaeota_archaeon_N4
Candidatus_Nitrososphaera_gargensis_Ga9
Thaumarchaeota_archaeon_JGI_OTU_3
Thaumarchaeota_archaeon_JGI_OTU_1
Candidatus_Caldiarchaeum_subterraneum
miscellaneous_Crenarchaeota_group_15_archaeon_DG_45
Bathyarchaeota_archaeon_BA2
Candidatus_Bathyarchaeota_archaeon_B24
Candidatus_Bathyarchaeota_archaeon_B25
Candidatus_Thorarchaeota_archaeon_AB_25
Candidatus_Heimdallarchaeota_archaeon_AB_125
Candidatus_Odinarchaeota_archaeon_LCB_4
Lokiarchaeum_sp_GC14_75
Hadesarchaea_archaeon_YNP_N21
Hadesarchaea_archaeon_DG_33
candidate_divison_MSBL1_archaeon_SCGC_AAA259I09
Thermococcus_barophilus_MP
Palaeococcus_pacificus_DY20341
Arc_I_group_archaeon_B15fssc0709_Meth_Bin003
Methanotorris_formicicus_Mc_S_70
Methanothermococcus_thermolithotrophicus_DSM_2095
Methanothermus_fervidus_DSM_2088
Methanothermobacter_thermautotrophicus_str_Delta_H
Candidatus_Altiarchaeales_archaeon_WOR_SM1_SCG
Candidatus_Altiarchaeales_archaeon_WOR_SM1_86_2
Methanospirillum_hungatei_JF_1
Methanoculleus_marisnigri_JR1
Methanosarcina_thermophila_TM_1
Methanocella_paludicola_SANAE
Archaeoglobus_veneficus_SNP6
Archaeoglobus_profundus_DSM_5631
Ferroglobus_placidus_DSM_10642
Thermoplasma_volcanium_GSS1
Aciduliprofundum_boonei_T469
Methanomassiliicoccus_luminyensis_B10
Thermoplasmatales_archaeon_SG8_52
0.2

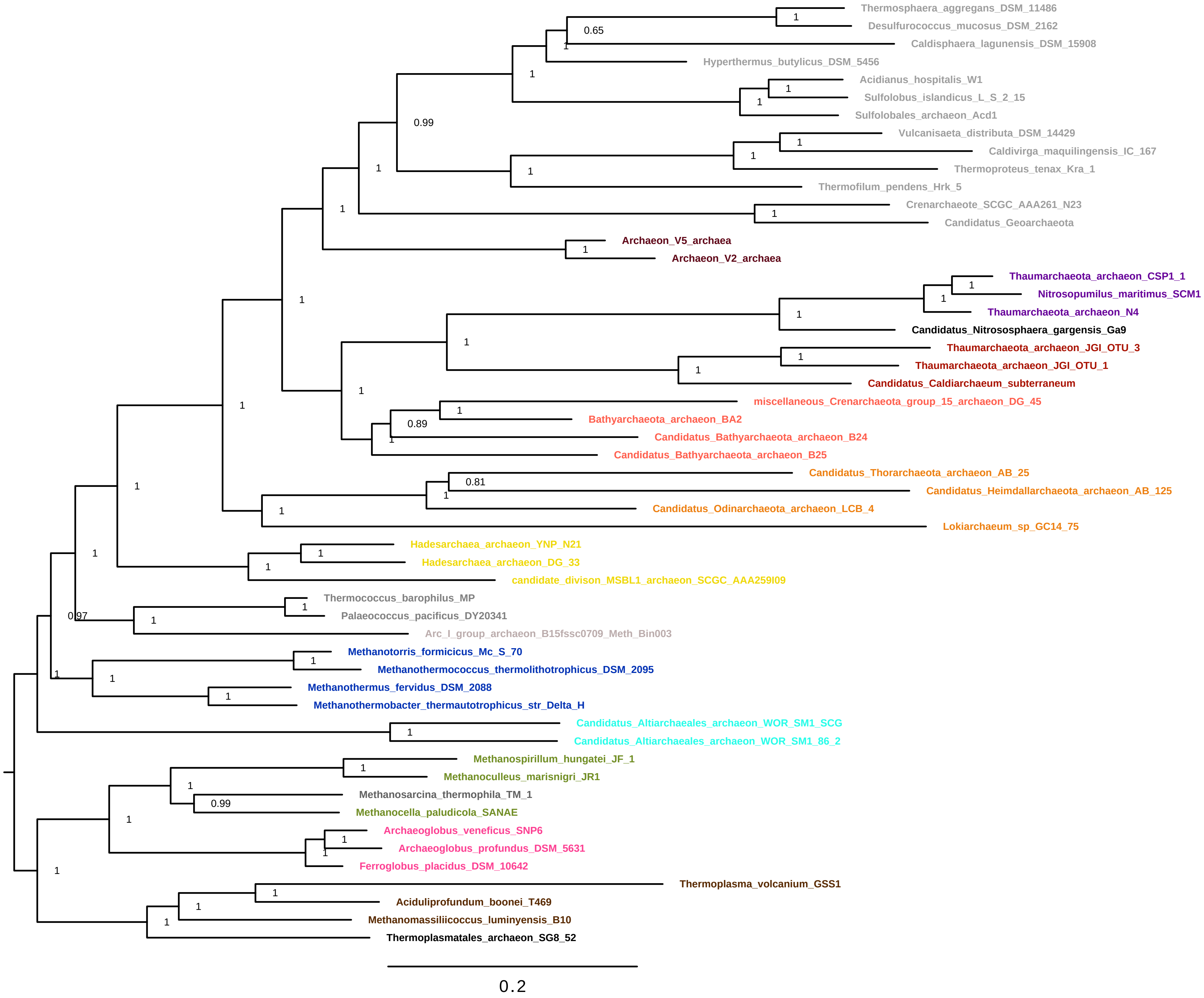
Thermosphaera_aggregans_DSM_11486
Desulfurococcus_mucosus_DSM_2162
Caldisphaera_lagunensis_DSM_15908
Hyperthermus_butylicus_DSM_5456
Acidianus_hospitalis_W1
Sulfolobus_islandicus_L_S_2_15
Sulfolobales_archaeon_Acd1
Vulcanisaeta_distributa_DSM_14429
Caldivirga_maquilingensis_IC_167
Thermoproteus_tenax_Kra_1
Thermofilum_pendens_Hrk_5
Crenarchaeote_SCGC_AAA261_N23
Candidatus_Geoarchaeota
Archaeon_V5_archaea
Archaeon_V2_archaea
Thaumarchaeota_archaeon_CSP1_1
Nitrosopumilus_maritimus_SCM1
Thaumarchaeota_archaeon_N4
Candidatus_Nitrososphaera_gargensis_Ga9
Thaumarchaeota_archaeon_JGI_OTU_3
Thaumarchaeota_archaeon_JGI_OTU_1
Candidatus_Caldiarchaeum_subterraneum
miscellaneous_Crenarchaeota_group_15_archaeon_DG_45
Bathyarchaeota_archaeon_BA2
Candidatus_Bathyarchaeota_archaeon_B24
Candidatus_Bathyarchaeota_archaeon_B25
Candidatus_Thorarchaeota_archaeon_AB_25
Candidatus_Heimdallarchaeota_archaeon_AB_125
Candidatus_Odinarchaeota_archaeon_LCB_4
Lokiarchaeum_sp_GC14_75
Hadesarchaea_archaeon_YNP_N21
Hadesarchaea_archaeon_DG_33
candidate_divison_MSBL1_archaeon_SCGC_AAA259I09
Thermococcus_barophilus_MP
Palaeococcus_pacificus_DY20341
Arc_I_group_archaeon_B15fssc0709_Meth_Bin003
Methanotorris_formicicus_Mc_S_70
Methanothermococcus_thermolithotrophicus_DSM_2095
Methanothermus_fervidus_DSM_2088
Methanothermobacter_thermautotrophicus_str_Delta_H
Candidatus_Altiarchaeales_archaeon_WOR_SM1_SCG
Candidatus_Altiarchaeales_archaeon_WOR_SM1_86_2
Methanospirillum_hungatei_JF_1
Methanoculleus_marisnigri_JR1
Methanosarcina_thermophila_TM_1
Methanocella_paludicola_SANAE
Archaeoglobus_veneficus_SNP6
Archaeoglobus_profundus_DSM_5631
Ferroglobus_placidus_DSM_10642
Thermoplasma_volcanium_GSS1
Aciduliprofundum_boonei_T469
Methanomassiliicoccus_luminyensis_B10
Thermoplasmatales_archaeon_SG8_52
0.2

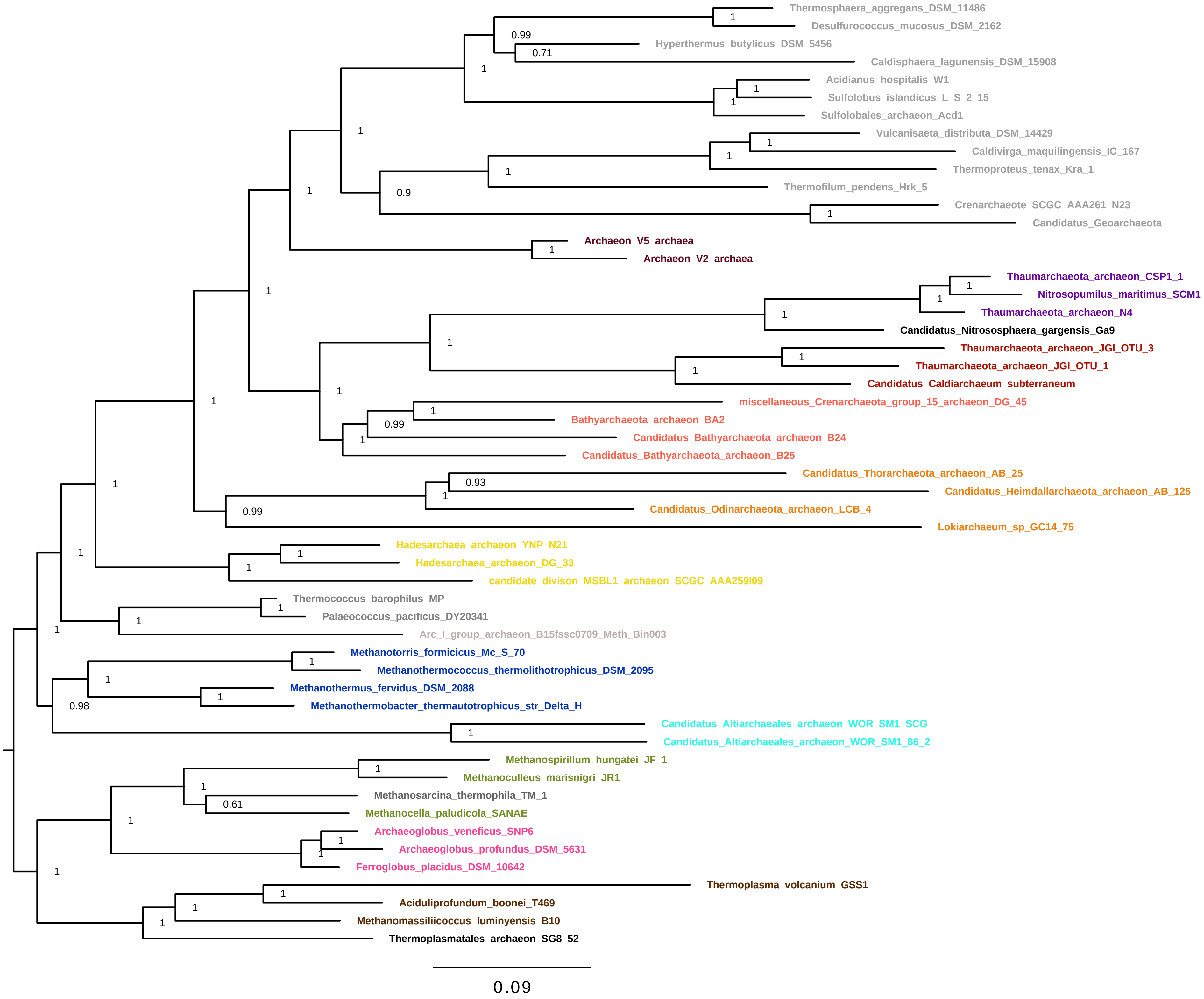
Thermosphaera_aggregans_DSM_11486
Desulfurococcus_mucosus_DSM_2162
Hyperthermus_butylicus_DSM_5456
Caldisphaera_lagunensis_DSM_15908
Acidianus_hospitalis_W1
Sulfolobus_islandicus_L_S_2_15
Sulfolobales_archaeon_Acd1
Vulcanisaeta_distributa_DSM_14429
Caldivirga_maquilingensis_IC_167
Thermoproteus_tenax_Kra_1
Thermofilum_pendens_Hrk_5
Crenarchaeote_SCGC_AAA261_N23
Candidatus_Geoarchaeota
Archaeon_V5_archaea
Archaeon_V2_archaea
Thaumarchaeota_archaeon_CSP1_1
Nitrosopumilus_maritimus_SCM1
Thaumarchaeota_archaeon_N4
Candidatus_Nitrososphaera_gargensis_Ga9
Thaumarchaeota_archaeon_JGI_OTU_3
Thaumarchaeota_archaeon_JGI_OTU_1
Candidatus_Caldiarchaeum_subterraneum
miscellaneous_Crenarchaeota_group_15_archaeon_DG_45
Bathyarchaeota_archaeon_BA2
Candidatus_Bathyarchaeota_archaeon_B24
Candidatus_Bathyarchaeota_archaeon_B25
Candidatus_Thorarchaeota_archaeon_AB_25
Candidatus_Heimdallarchaeota_archaeon_AB_125
Candidatus_Odinarchaeota_archaeon_LCB_4
Lokiarchaeum_sp_GC14_75
Hadesarchaea_archaeon_YNP_N21
Hadesarchaea_archaeon_DG_33
candidate_divison_MSBL1_archaeon_SCGC_AAA259I09
Thermococcus_barophilus_MP
Palaeococcus_pacificus_DY20341
Arc_I_group_archaeon_B15fssc0709_Meth_Bin003
Methanotorris_formicicus_Mc_S_70
Methanothermococcus_thermolithotrophicus_DSM_2095
Methanothermus_fervidus_DSM_2088
Methanothermobacter_thermautotrophicus_str_Delta_H
Candidatus_Altiarchaeales_archaeon_WOR_SM1_SCG
Candidatus_Altiarchaeales_archaeon_WOR_SM1_86_2
Methanospirillum_hungatei_JF_1
Methanoculleus_marisnigri_JR1
Methanosarcina_thermophila_TM_1
Methanocella_paludicola_SANAE
Archaeoglobus_veneficus_SNP6
Archaeoglobus_profundus_DSM_5631
Ferroglobus_placidus_DSM_10642
Thermoplasma_volcanium_GSS1
Aciduliprofundum_boonei_T469
Methanomassiliicoccus_luminyensis_B10
Thermoplasmatales_archaeon_SG8_52
0.09

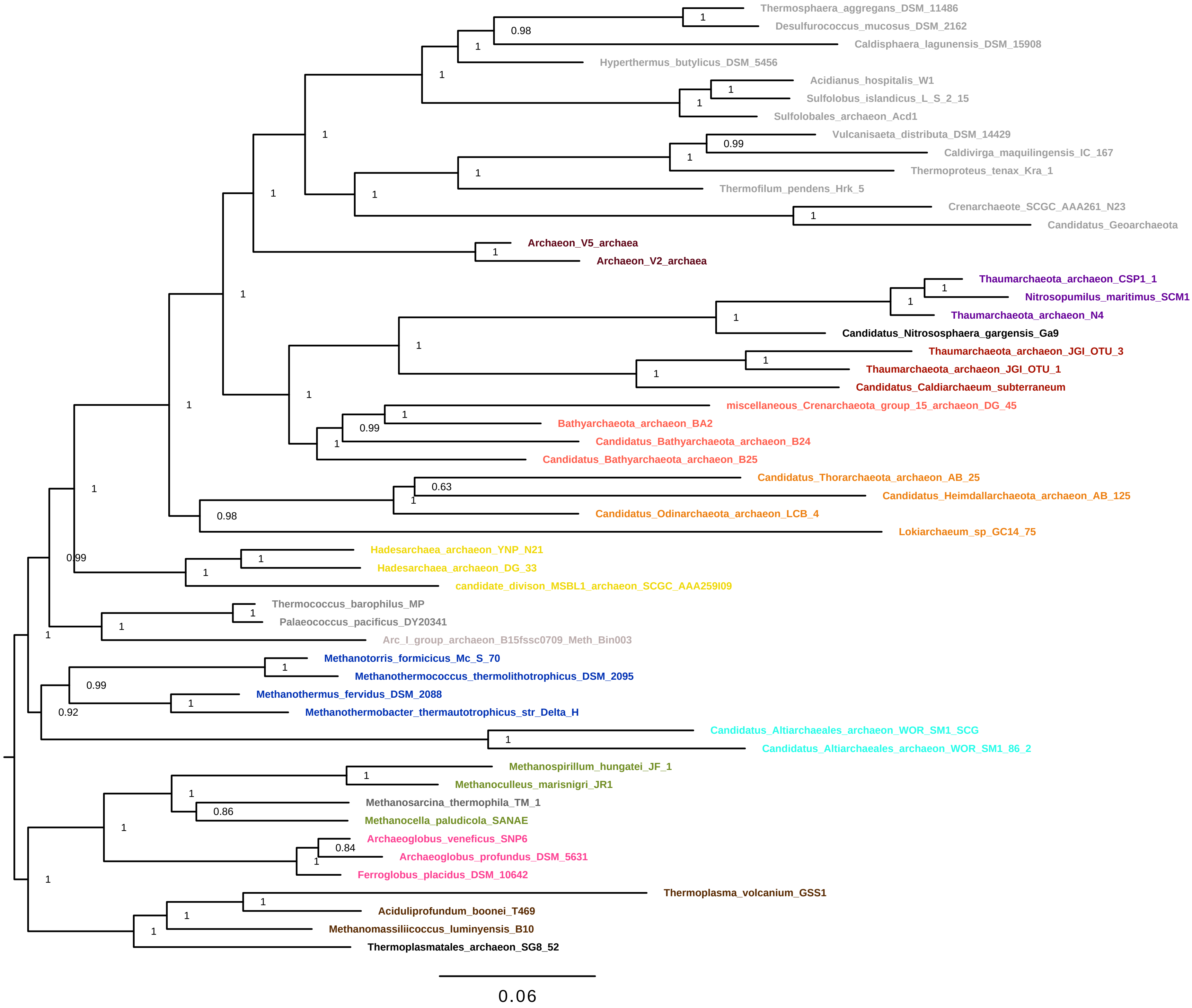
Thermosphaera_aggregans_DSM_11486
Desulfurococcus_mucosus_DSM_2162
Caldisphaera_lagunensis_DSM_15908
Hyperthermus_butylicus_DSM_5456
Acidianus_hospitalis_W1
Sulfolobus_islandicus_L_S_2_15
Sulfolobales_archaeon_Acd1
Vulcanisaeta_distributa_DSM_14429
Caldivirga_maquilingensis_IC_167
Thermoproteus_tenax_Kra_1
Thermofilum_pendens_Hrk_5
Crenarchaeote_SCGC_AAA261_N23
Candidatus_Geoarchaeota
Archaeon_V5_archaea
Archaeon_V2_archaea
Thaumarchaeota_archaeon_CSP1_1
Nitrosopumilus_maritimus_SCM1
Thaumarchaeota_archaeon_N4
Candidatus_Nitrososphaera_gargensis_Ga9
Thaumarchaeota_archaeon_JGI_OTU_3
Thaumarchaeota_archaeon_JGI_OTU_1
Candidatus_Caldiarchaeum_subterraneum
miscellaneous_Crenarchaeota_group_15_archaeon_DG_45
Bathyarchaeota_archaeon_BA2
Candidatus_Bathyarchaeota_archaeon_B24
Candidatus_Bathyarchaeota_archaeon_B25
Candidatus_Thorarchaeota_archaeon_AB_25
Candidatus_Heimdallarchaeota_archaeon_AB_125
Candidatus_Odinarchaeota_archaeon_LCB_4
Lokiarchaeum_sp_GC14_75
Hadesarchaea_archaeon_YNP_N21
Hadesarchaea_archaeon_DG_33
candidate_divison_MSBL1_archaeon_SCGC_AAA259I09
Thermococcus_barophilus_MP
Palaeococcus_pacificus_DY20341
Arc_I_group_archaeon_B15fssc0709_Meth_Bin003
Methanotorris_formicicus_Mc_S_70
Methanothermococcus_thermolithotrophicus_DSM_2095
Methanothermus_fervidus_DSM_2088
Methanothermobacter_thermautotrophicus_str_Delta_H
Candidatus_Altiarchaeales_archaeon_WOR_SM1_SCG
Candidatus_Altiarchaeales_archaeon_WOR_SM1_86_2
Methanospirillum_hungatei_JF_1
Methanoculleus_marisnigri_JR1
Methanosarcina_thermophila_TM_1
Methanocella_paludicola_SANAE
Archaeoglobus_veneficus_SNP6
Archaeoglobus_profundus_DSM_5631
Ferroglobus_placidus_DSM_10642
Thermoplasma_volcanium_GSS1
Aciduliprofundum_boonei_T469
Methanomassiliicoccus_luminyensis_B10
Thermoplasmatales_archaeon_SG8_52
0.06

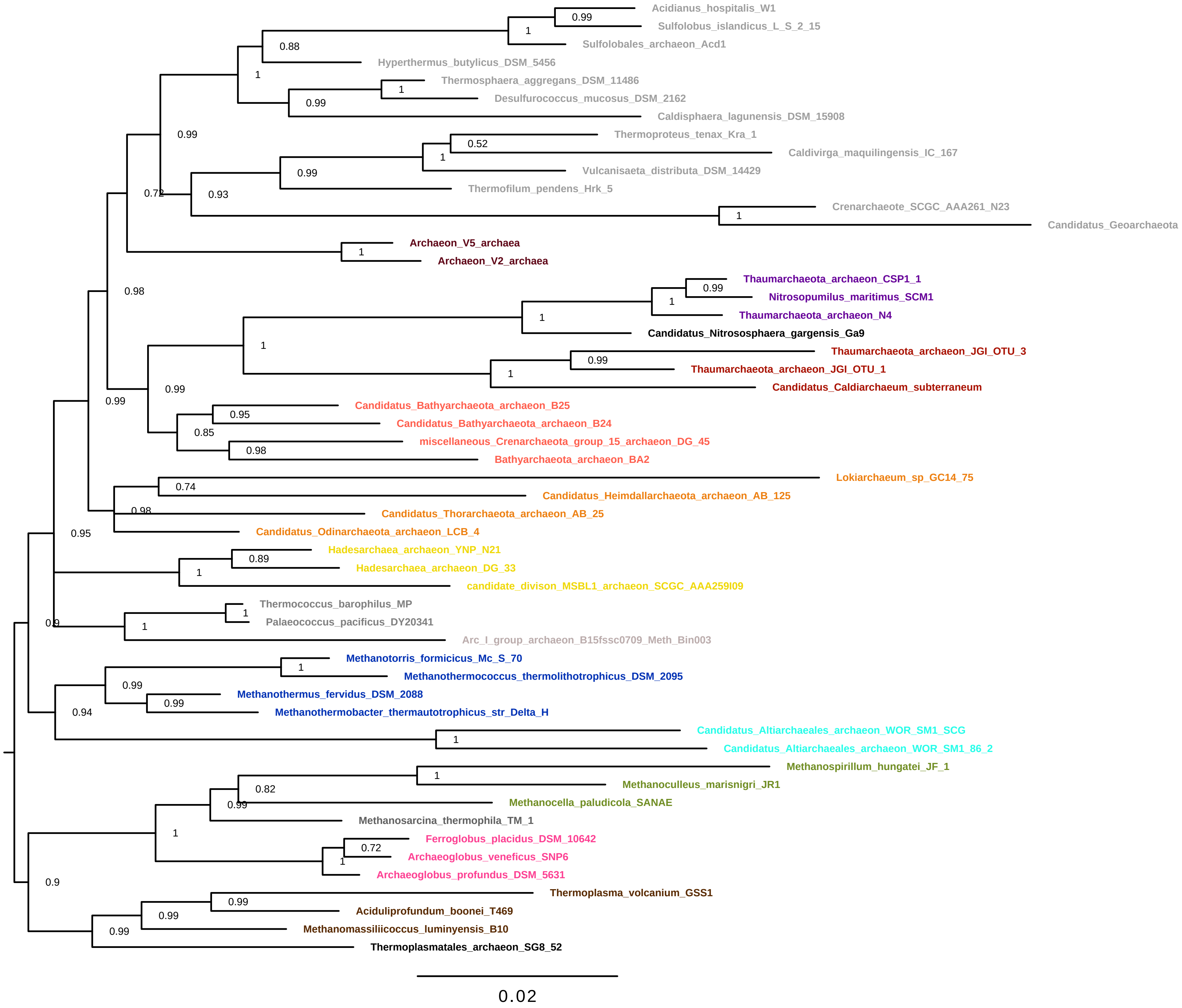
Acidianus_hospitalis_W1
Sulfolobus_islandicus_L_S_2_15
Sulfolobales_archaeon_Acd1
Hyperthermus_butylicus_DSM_5456
Thermosphaera_aggregans_DSM_11486
Desulfurococcus_mucosus_DSM_2162
Caldisphaera_lagunensis_DSM_15908
Thermoproteus_tenax_Kra_1
Caldivirga_maquilingensis_IC_167
Vulcanisaeta_distributa_DSM_14429
Thermofilum_pendens_Hrk_5
Crenarchaeote_SCGC_AAA261_N23
Candidatus_Geoarchaeota
Archaeon_V5_archaea
Archaeon_V2_archaea
Thaumarchaeota_archaeon_CSP1_1
Nitrosopumilus_maritimus_SCM1
Thaumarchaeota_archaeon_N4
Candidatus_Nitrososphaera_gargensis_Ga9
Thaumarchaeota_archaeon_JGI_OTU_3
Thaumarchaeota_archaeon_JGI_OTU_1
Candidatus_Caldiarchaeum_subterraneum
Candidatus_Bathyarchaeota_archaeon_B25
Candidatus_Bathyarchaeota_archaeon_B24
miscellaneous_Crenarchaeota_group_15_archaeon_DG_45
Bathyarchaeota_archaeon_BA2
Lokiarchaeum_sp_GC14_75
Candidatus_Heimdallarchaeota_archaeon_AB_125
Candidatus_Thorarchaeota_archaeon_AB_25
Candidatus_Odinarchaeota_archaeon_LCB_4
Hadesarchaea_archaeon_YNP_N21
Hadesarchaea_archaeon_DG_33
candidate_divison_MSBL1_archaeon_SCGC_AAA259I09
Thermococcus_barophilus_MP
Palaeococcus_pacificus_DY20341
Arc_I_group_archaeon_B15fssc0709_Meth_Bin003
Methanotorris_formicicus_Mc_S_70
Methanothermococcus_thermolithotrophicus_DSM_2095
Methanothermus_fervidus_DSM_2088
Methanothermobacter_thermautotrophicus_str_Delta_H
Candidatus_Altiarchaeales_archaeon_WOR_SM1_SCG
Candidatus_Altiarchaeales_archaeon_WOR_SM1_86_2
Methanospirillum_hungatei_JF_1
Methanoculleus_marisnigri_JR1
Methanocella_paludicola_SANAE
Methanosarcina_thermophila_TM_1
Ferroglobus_placidus_DSM_10642
Archaeoglobus_veneficus_SNP6
Archaeoglobus_profundus_DSM_5631
Thermoplasma_volcanium_GSS1
Aciduliprofundum_boonei_T469
Methanomassiliicoccus_luminyensis_B10
Thermoplasmatales_archaeon_SG8_52
0.99
1
0.88
1
0.99
1
0.99
0.52
1
0.99
0.72
0.93
1
1
0.98
0.99
1
1
1
0.99
1
0.99
0.99
0.95
0.85
0.98
0.74
0.98
0.95
0.89
1
1
1
0.9
1
0.99
0.99
0.94
1
1
0.82
0.99
1
0.72
1
0.9
0.99
0.99
0.99
0.02

Supplementary Figure S5. Unrooted Bayesian phylogeny of *Archaea and Eucarya*.

The tree corresponds to the AE supermatrix (61 protein families, 236 taxa, 13,468 amino acids positions). The tree was inferred with PHYLOBAYES using the CAT+GTR+G4 model. Values at branch correspond to branch lengths (top) and posterior probabilities (bottom). The scale bar indicates the average number of substitutions per site.

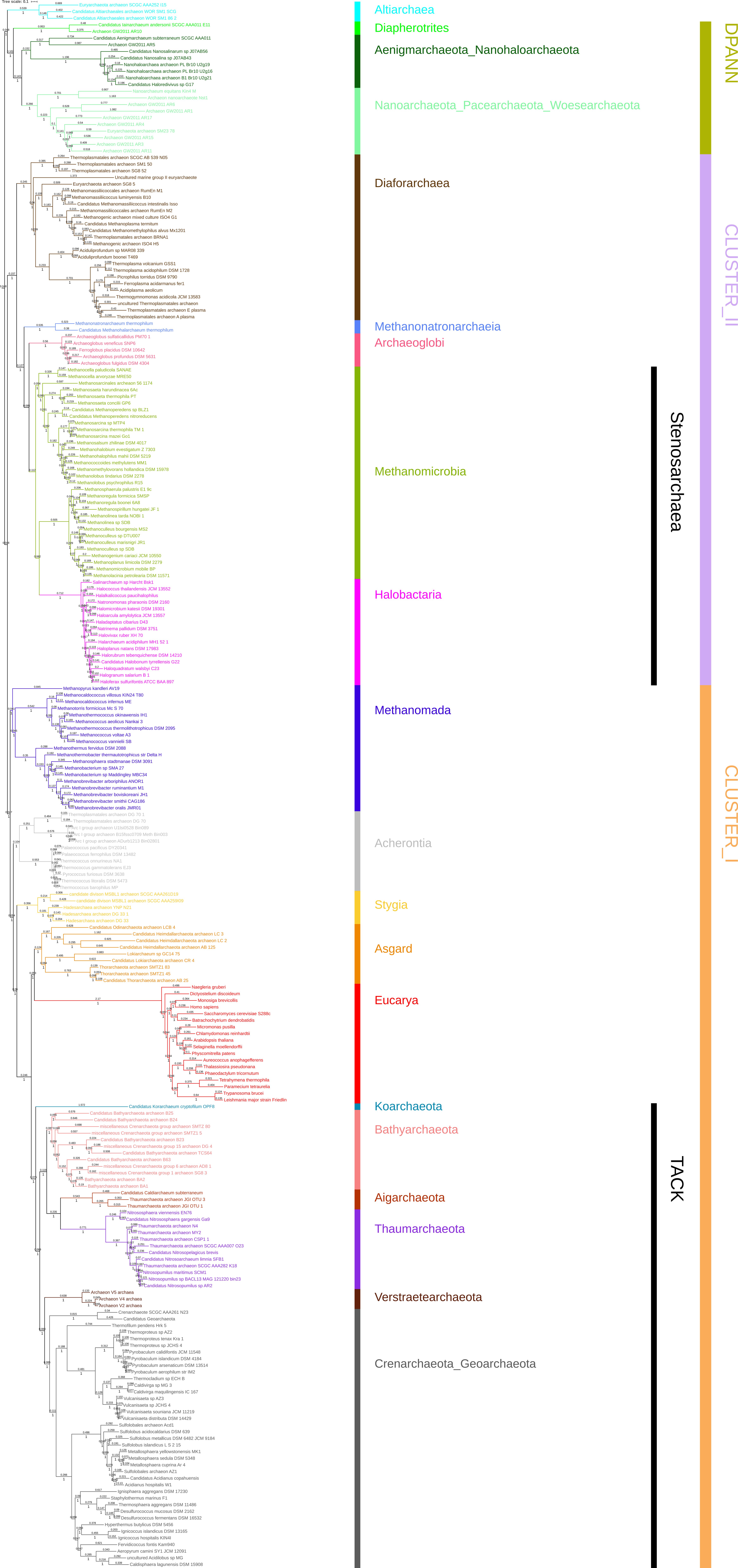
Tree scale: 0.1
Altiarchaea
Diapherotrites
Aenigmarchaeota_Nanohaloarchaeota
Nanoarchaeota_Pacearchaeota_Woesearchaeota
Diaforarchaea
Methanonatronarchaeia
Archaeoglobi
Methanomicrobia
Halobactaria
Methanomada
Acherontia
Stygia
Asgard
Eucarya
Koarchaeota
Bathyarchaeota
Aigarchaeota
Thaumarchaeota
Verstraetearchaeota
Crenarchaeota_Geoarchaeota
DPANN
CLUSTER_II
Stenosarchaea
CLUSTER_I
TACK

Supplementary Figure S6. Unrooted maximum likelihood phylogeny of *Archaea and Eucarya*.

The tree corresponds to the AE supermatrix (61 protein families, 236 taxa, 13,468 amino acids positions). The tree was inferred with IQ-TREE using the LG+C20+G4 model. Values at branch correspond to branch lengths (top) and bootstrap values (bottom). The scale bar indicates the average number of substitutions per site.

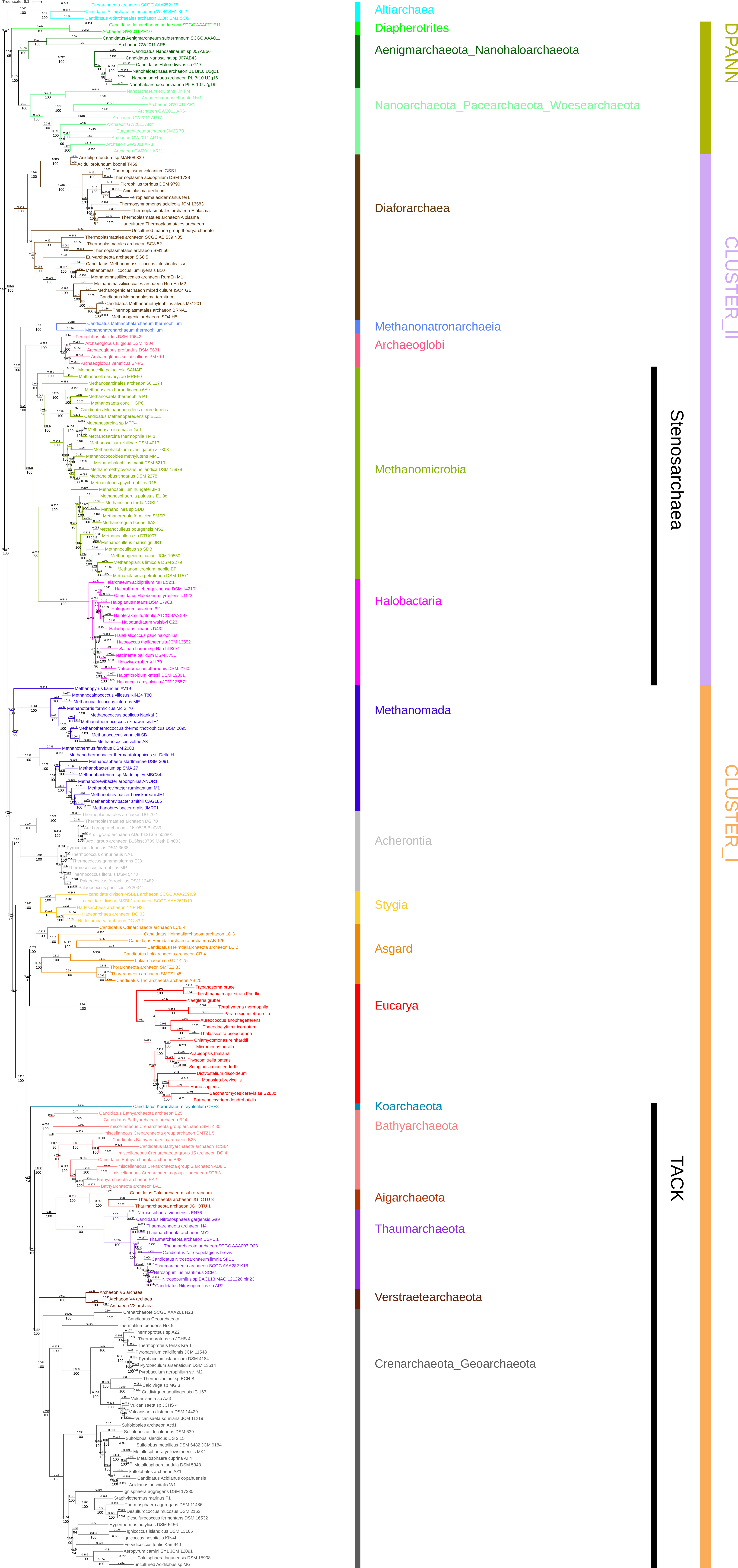

Tree scale: 0.1
Altiarchaea
Diapherotrites
Aenigmarchaeota_Nanohaloarchaeota
Nanoarchaeota_Pacearchaeota_Woesearchaeota
Diaforarchaea
Methanonatronarchaeia
Archaeoglobi
Methanomicrobia
Halobactaria
Methanomada
Acherontia
Stygia
Asgard
Eucarya
Koarchaeota
Bathyarchaeota
Aigarchaeota
Thaumarchaeota
Verstraetearchaeota
Crenarchaeota_Geoarchaeota
DPANN
CLUSTER_II
Stenosarchaea
CLUSTER_I
TACK

Supplementary Figure S7. Phylogenetic position of the *Eucarya* relatively to Cluster I lineages by Dayhoff4 recoding.

Unrooted Bayesian phylogenetic tree built using the AE supermatrix (64 protein families, 114 taxa, 13,468 amino acids positions). The tree was inferred with PHYLOBAYES using the CAT+GTR+G4 model and the amino acid recoding scheme Dayhoff4. The length of the branches corresponds to evolutionary distances. Supports at branch correspond to posterior probabilities.

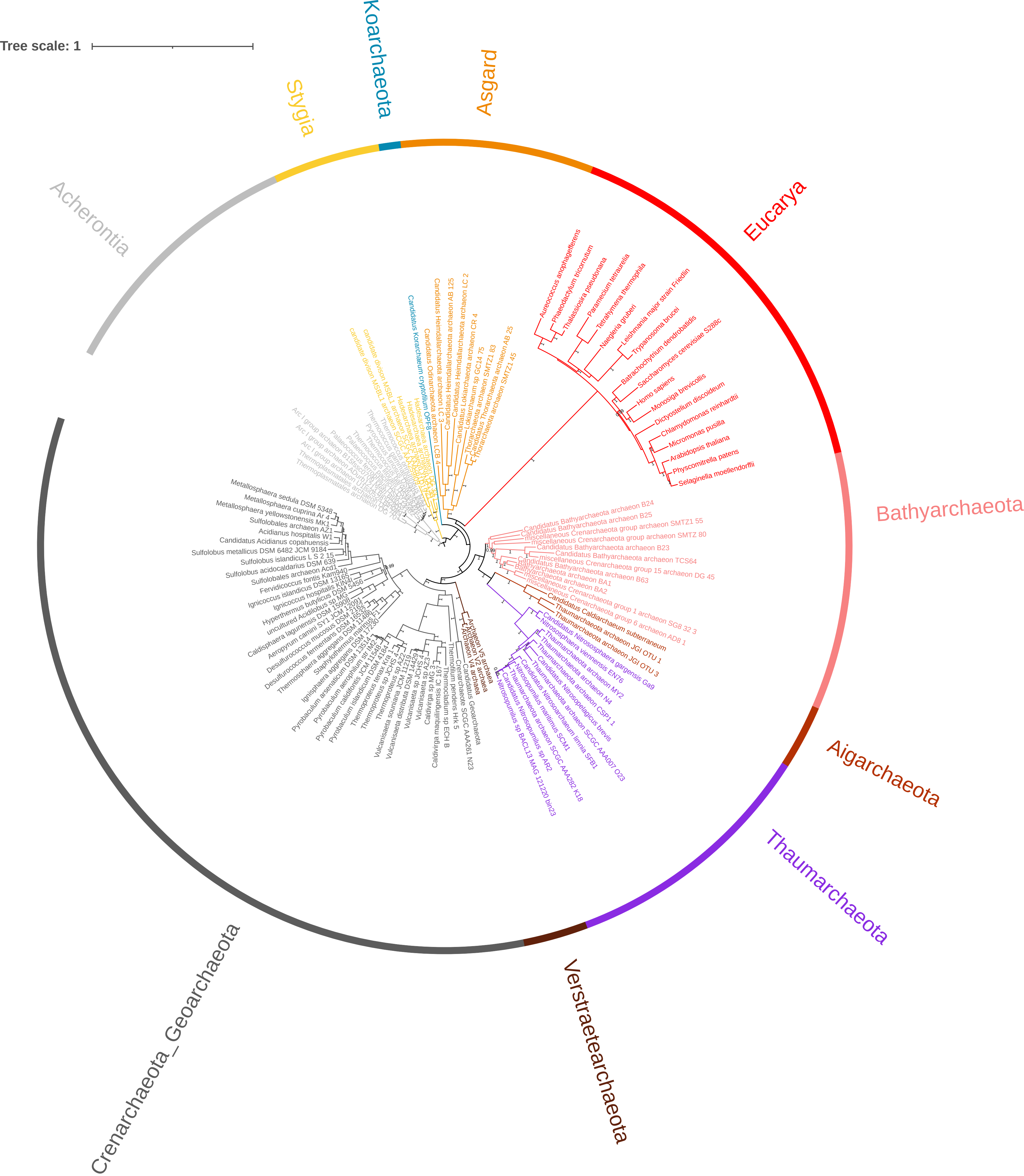
Tree scale: 1
Koarchaeota
Asgard
Stygia
Acherontia
Eucarya
Bathyarchaeota
Aigarchaeota
Thaumarchaeota
Verstraetearchaeota
Crenarchaeota_Geoarchaeota
Aureococcus anophagefferens
Phaeodactylum tricornutum
Thalassiosira pseudonana
Paramecium tetraurelia
Tetrahymena thermophila
Naegleria gruberi
Leishmania major strain Friedlin
Trypanosoma brucei
Batrachochytrium dendrobatidis
Saccharomyces cerevisiae S288c
Homo sapiens
Monosiga brevicollis
Dictyostelium discoideum
Chlamydomonas reinhardtii
Micromonas pusilla
Arabidopsis thaliana
Physcomitrella patens
Selaginella moellendorffii
Candidatus Bathyarchaeota archaeon B24
Candidatus Bathyarchaeota archaeon B25
miscellaneous Crenarchaeota group archaeon SMTZ1 55
miscellaneous Crenarchaeota group archaeon SMTZ 80
Candidatus Bathyarchaeota archaeon B23
Candidatus Bathyarchaeota archaeon TCS64
miscellaneous Crenarchaeota group 15 archaeon DG 45
Candidatus Bathyarchaeota archaeon B63
Bathyarchaeota archaeon BA1
Bathyarchaeota archaeon BA2
miscellaneous Crenarchaeota group 1 archaeon SG8 32 3
miscellaneous Crenarchaeota group 6 archaeon AD8 1
Candidatus Caldiarchaeum subterraneum
Thaumarchaeota archaeon JGI OTU 1
Thaumarchaeota archaeon JGI OTU 3
Candidatus Nitrososphaera gargensis Ga9
Nitrososphaera viennensis EN76
Thaumarchaeota archaeon MY2
Thaumarchaeota archaeon N4
Thaumarchaeota archaeon CSP1 1
Candidatus Nitrosopelagicus brevis
Thaumarchaeota archaeon SCGC AA007 O23
Candidatus Nitrosoarchaeum limnia SFB1
Nitrosopumilus maritimus SCM1
Thaumarchaeota archaeon SCGC AAA282 K18
Nitrosopumilus sp AR2
Candidatus Nitrosopumilus sp BACL13 MAG 121220 bin23
Archaeon V5 archaea
Archaeon V2 archaea
Archaeon V4 archaea
Candidatus Geoarchaeota
Crenarchaeote SCGC AAA261 N23
Thermofilum pendens Hrk 5
Thermocladium sp ECH B
Caldivirga maquilingensis IC 167
Caldivirga sp MG 3
Vulcanisaeta sp AZ3
Vulcanisaeta sp JCHS 4
Vulcanisaeta distributa DSM 14429
Vulcanisaeta souniana JCM 11219
Thermoproteus sp JCHS 4
Thermoproteus tenax Kra 1
Metallosphaera sedula DSM 5348
Metallosphaera cuprina Ar 4
Metallosphaera yellowstonensis MK1
Sulfolobales archaeon AZ1
Acidianus hospitalis W1
Candidatus Acidianus copahuensis
Sulfolobus metallicus DSM 6482 JCM 9184
Sulfolobus islandicus L S 2 15
Sulfolobus acidocaldarius DSM 639
Sulfolobales archaeon Acd1
Fervidicoccus fontis Kam940
Candidatus Korarchaeum cryptofilum OPF8
Candidatus Heimdallarchaeota archaeon AB 125
Candidatus Heimdallarchaeota archaeon LC 2
Candidatus Heimdallarchaeota archaeon LC 3
Candidatus Odinarchaeota archaeon LCB 4
Candidatus Lokiarchaeota archaeon CR 4
Lokiarchaeum sp GC14 75
Thorarchaeota archaeon SMTZ1 83
Candidatus Thorarchaeota archaeon AB 25
Thorarchaeota archaeon SMTZ1 45

Supplementary Figure S8. Phylogenetic position of the *Eucarya* relatively to Cluster I lineages by Dayhoff6 recoding.

Unrooted Bayesian phylogenetic tree built using the AE supermatrix (64 protein families, 114 taxa, 13,468 amino acids positions). The tree was inferred with PHYLOBAYES using the CAT+GTR+G4 model and the amino acid recoding scheme Dayhoff6. The length of the branches corresponds to evolutionary distances. Supports at branch correspond to posterior probabilities.

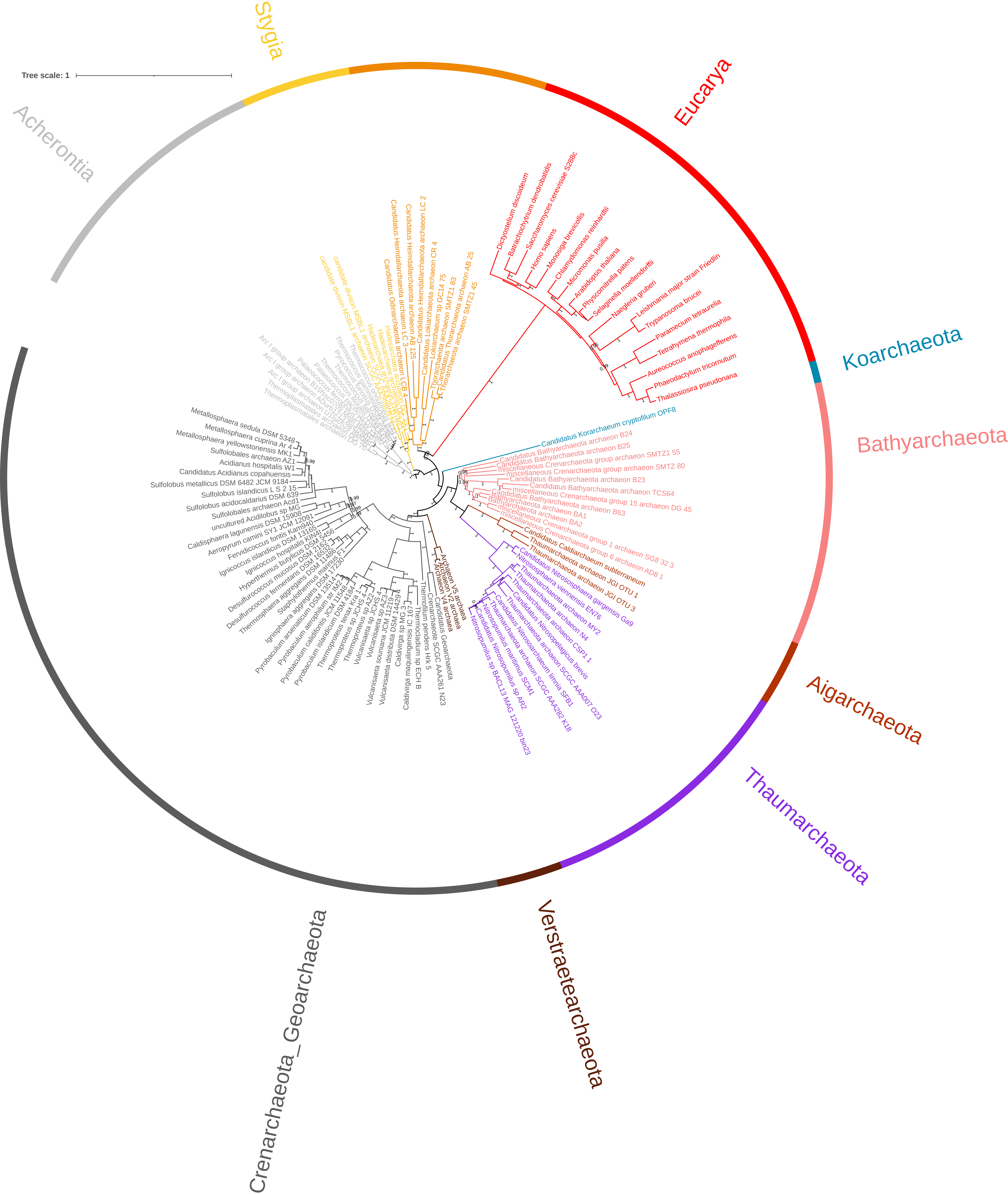
Tree scale: 1
Stygia
Eucarya
Koarchaeota
Bathyarchaeota
Aigarchaeota
Thaumarchaeota
Verstraetearchaeota
Crenarchaeota_Geoarchaeota
Acherontia
Dictyostelium discoideum
Batrachochytrium dendrobatidis
Saccharomyces cerevisiae S288c
Homo sapiens
Monosiga brevicollis
Chlamydomonas reinhardtii
Micromonas pusilla
Arabidopsis thaliana
Physcomitrella patens
Selaginella moellendorffii
Naegleria gruberi
Leishmania major strain Friedlin
Trypanosoma brucei
Paramecium tetraurelia
Tetrahymena thermophila
Aureococcus anophagefferens
Phaeodactylum tricornutum
Thalassiosira pseudonana
Candidatus Korarchaeum cryptofilum OPF8
Candidatus Bathyarchaeota archaeon B24
Candidatus Bathyarchaeota archaeon B25
miscellaneous Crenarchaeota group archaeon SMTZ1 55
miscellaneous Crenarchaeota group archaeon SMTZ 80
Candidatus Bathyarchaeota archaeon B23
Candidatus Bathyarchaeota archaeon TCS64
miscellaneous Crenarchaeota group 15 archaeon DG 45
Candidatus Bathyarchaeota archaeon B63
Bathyarchaeota archaeon BA1
Bathyarchaeota archaeon BA2
miscellaneous Crenarchaeota group 1 archaeon SG8 32 3
miscellaneous Crenarchaeota group 6 archaeon AD8 1
Candidatus Caldiarchaeum subterraneum
Thaumarchaeota archaeon JGI OTU 1
Thaumarchaeota archaeon JGI OTU 3
Candidatus Nitrososphaera gargensis Ga9
Nitrososphaera viennensis EN76
Thaumarchaeota archaeon MY2
Thaumarchaeota archaeon N4
Thaumarchaeota archaeon CSP1 1
Candidatus Nitrosopelagicus brevis
Thaumarchaeota archaeon SCGC AAA007 O23
Candidatus Nitrosoarchaeum limnia SFB1
Thaumarchaeota archaeon SCGC AAA282 K18
Candidatus Nitrosopumilus sp AR2
Nitrosopumilus maritimus SCM1
Candidatus Nitrosopumilus sp BACL13 MAG 121220 bin23
Archaeon V5 archaea
Archaeon V2 archaea
Archaeon V4 archaea
Candidatus Geoarchaeota
Crenarchaeote SCGC AAA261 N23
Thermofilum pendens Hrk 5
Thermocladium sp ECH B
Caldivirga maquilingensis IC 167
Caldivirga sp MG 3
Vulcanisaeta distributa DSM 14429
Vulcanisaeta souniana JCM 11219
Vulcanisaeta sp JCHS 4
Vulcanisaeta sp AZ3
Thermoproteus sp AZ2
Thermoproteus sp JCHS 4
Thermoproteus tenax Kra 1
Pyrobaculum islandicum DSM 4184
Pyrobaculum calidifontis JCM 11548
Pyrobaculum aerophilum str IM2
Pyrobaculum arsenaticum DSM 13514
Ignisphaera aggregans DSM 17230
Staphylothermus marinus F1
Thermosphaera aggregans DSM 11486
Desulfurococcus fermentans DSM 16532
Desulfurococcus mucosus DSM 2162
Hyperthermus butylicus DSM 5456
Ignicoccus hospitalis KIN4I
Ignicoccus islandicus DSM 13165
Fervidicoccus fontis Kam940
Aeropyrum camini SY1 JCM 12091
Caldisphaera lagunensis DSM 15908
uncultured Acidilobus sp MG
Sulfolobales archaeon Acd1
Sulfolobus acidocaldarius DSM 639
Sulfolobus islandicus L S 2 15
Sulfolobus metallicus DSM 6482 JCM 9184
Candidatus Acidianus copahuensis
Acidianus hospitalis W1
Sulfolobales archaeon AZ1
Metallosphaera yellowstonensis MK1
Metallosphaera cuprina Ar 4
Metallosphaera sedula DSM 5348
Candidatus Odinarchaeota archaeon LCB 4
Candidatus Heimdallarchaeota archaeon LC 3
Candidatus Heimdallarchaeota archaeon AB 125
Candidatus Heimdallarchaeota archaeon LC 2
Candidatus Lokiarchaeota archaeon CR 4
Lokiarchaeum sp GC14 75
Thorarchaeota archaeon SMTZ1 83
Candidatus Thorarchaeota archaeon AB 25
Thorarchaeota archaeon SMTZ1 45
candidate divison MSBL1 archaeon
Arc I group archaeon
Thermoplasmatales archaeon DG 70

Supplementary Figure S9. Rooted Bayesian phylogeny of the *Archaea*.

The tree corresponds to the AB supermatrix (41 protein families, 285 taxa, 7,853 amino acids positions). The tree was inferred with PHYLOBAYES using the CAT+GTR+G4 model. Values at branch correspond to branch lengths (top) and posterior probabilities (bottom). The scale bar indicates the average number of substitutions per site.

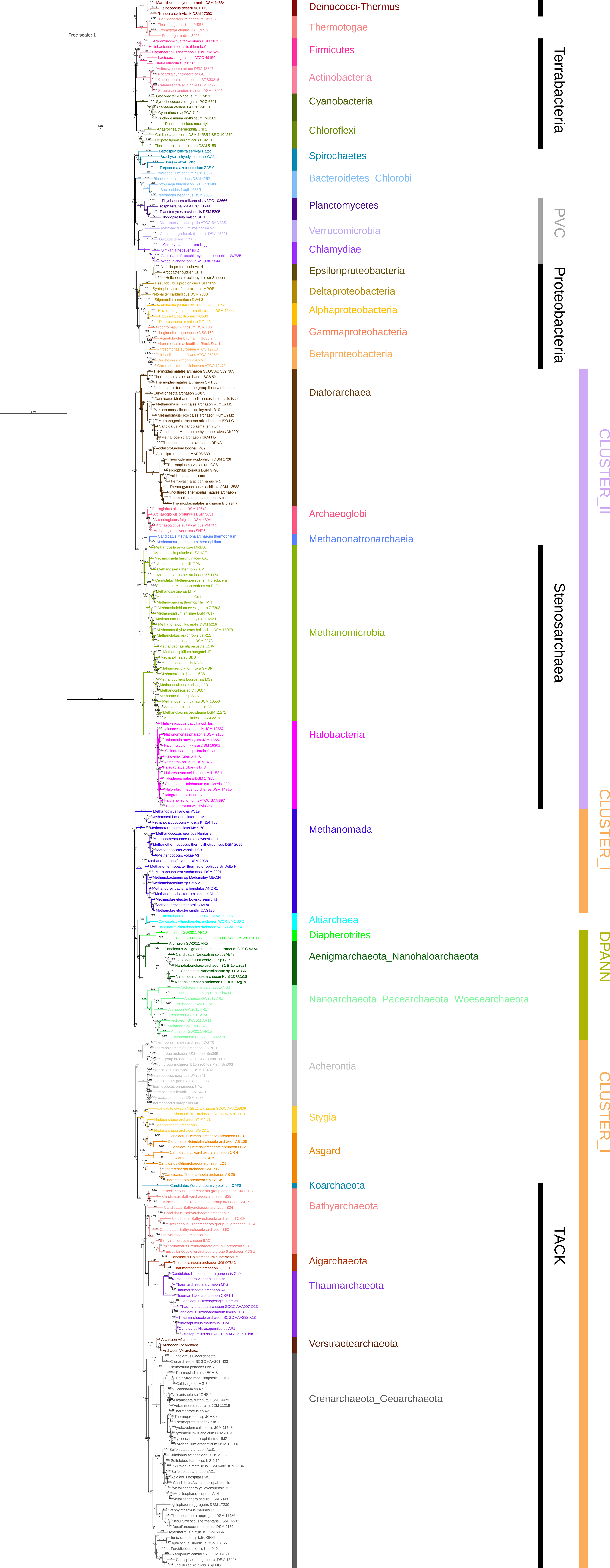

Tree scale: 1
Deinococci-Thermus
Thermotogae
Firmicutes
Actinobacteria
Cyanobacteria
Chloroflexi
Spirochaetes
Bacteroidetes_Chlorobi
Planctomycetes
Verrucomicrobia
Chlamydiae
Epsilonproteobacteria
Deltaproteobacteria
Alphaproteobacteria
Gammaproteobacteria
Betaproteobacteria
Diaforarchaea
Archaeoglobi
Methanonatronarchaeia
Methanomicrobia
Halobacteria
Methanomada
Altiarchaea
Diapherotrites
Aenigmarchaeota_Nanohaloarchaeota
Nanoarchaeota_Pacearchaeota_Woesearchaeota
Acherontia
Stygia
Asgard
Koarchaeota
Bathyarchaeota
Aigarchaeota
Thaumarchaeota
Verstraetearchaeota
Crenarchaeota_Geoarchaeota
Terrabacteria
PVC
Proteobacteria
Stenosarchaea
TACK
CLUSTER_II
CLUSTER_I
DPANN
CLUSTER_I

Supplementary Figure S10. Rooted maximum likelihood phylogeny of *Archaea*.

The tree corresponds to the AB supermatrix (41 protein families, 285 taxa, 7,853 amino acids positions). The tree was inferred with IQ-TREE using the LG+C20+G4 model. Values at branch correspond to branch lengths (top) and bootstrap values (bottom). The scale bar indicates the average number of substitutions per site.

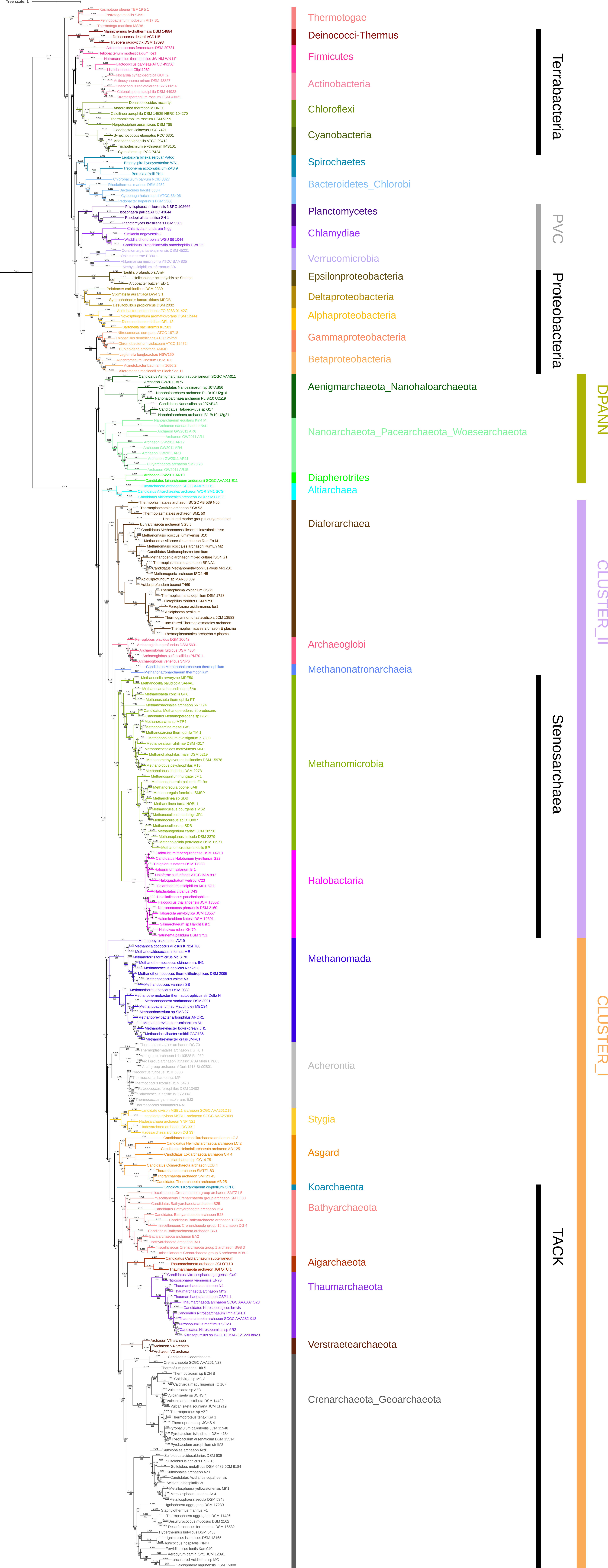

Tree scale: 1
Thermotogae
Deinococci-Thermus
Firmicutes
Actinobacteria
Chloroflexi
Cyanobacteria
Spirochaetes
Bacteroidetes_Chlorobi
Planctomycetes
Chlamydiae
Verrucomicrobia
Epsilonproteobacteria
Deltaproteobacteria
Alphaproteobacteria
Gammaproteobacteria
Betaproteobacteria
Terrabacteria
PVC
Proteobacteria
Aenigmarchaeota_Nanohaloarchaeota
Nanoarchaeota_Pacearchaeota_Woesearchaeota
Diapherotrites
Altiarchaea
DPANN
Diaforarchaea
Archaeoglobi
Methanonatronarchaeia
Methanomicrobia
Halobactaria
Methanomada
Acherontia
Stygia
Asgard
Koarchaeota
Bathyarchaeota
Aigarchaeota
Thaumarchaeota
Verstraetearchaeota
Crenarchaeota_Geoarchaeota
CLUSTER_II
CLUSTER_I
Stenosarchaea
TACK

Supplementary Figure S11. Rooted Bayesian phylogeny of *Archaea without DPANN and Altiarchaea*. The tree corresponds to a version AB supermatrix built without DPANN and *Altiarchaea* (41 protein families, 261 taxa, 7,952 amino acids positions). Values at branch correspond to branch lengths (top) and posterior probabilities (bottom). The scale bar indicates the average number of substitutions per site.

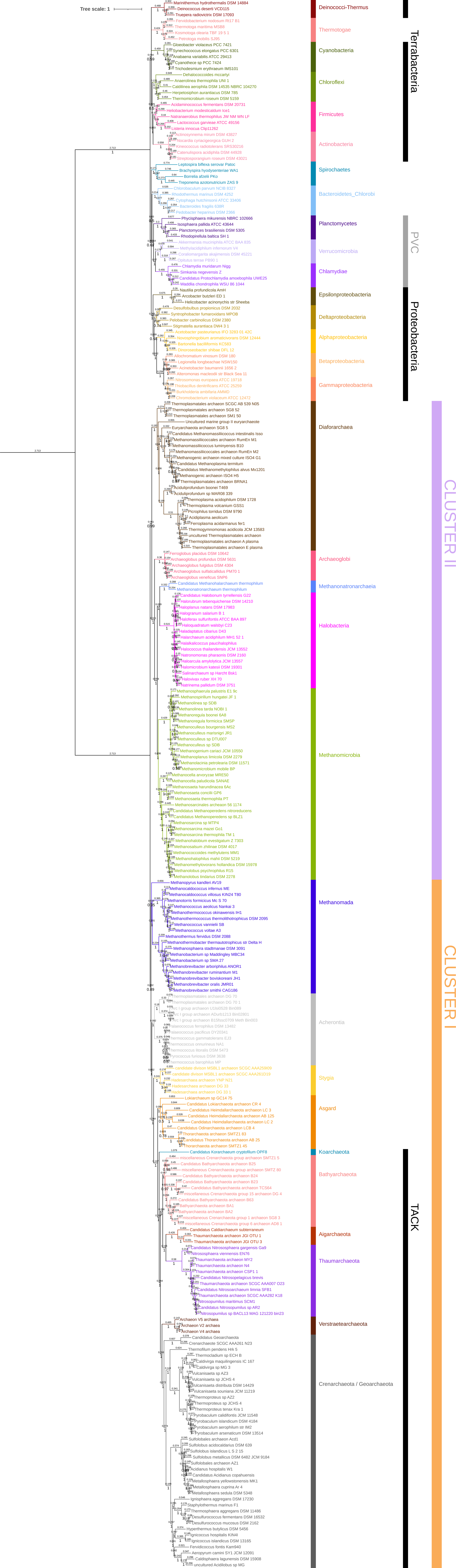
Tree scale: 1
Deinococci-Thermus
Thermotogae
Cyanobacteria
Chloroflexi
Firmicutes
Actinobacteria
Spirochaetes
Bacteroidetes_Chlorobi
Planctomycetes
Verrucomicrobia
Chlamydiae
Epsilonproteobacteria
Deltaproteobacteria
Alphaproteobacteria
Betaproteobacteria
Gammaproteobacteria
Terrabacteria
PVC
Proteobacteria
Diaforarchaea
Archaeoglobi
Methanonatronarchaeia
Halobacteria
Methanomicrobia
Methanomada
Acherontia
Stygia
Asgard
Koarchaeota
Bathyarchaeota
Aigarchaeota
Thaumarchaeota
Verstraetearchaeota
Crenarchaeota / Geoarchaeota
TACK
CLUSTER II
CLUSTER I

Supplementary Table S1: Liste of the taxa used in the study (435 archaea, 67 bacteria, and 18 eukaryotes). The 42 archaeal species useid in the study by Raymann et al. (2015) are indicated. The 218 representative archaeal species kept for phylogenetic analyses are in green. Their presence in the 72 archaeal protein families used in this study is indicated. Representative species kept for the Slow-Fast analysis are in dark green.

Domain	Taxon name	Superphylum or phylum	Present in Raymann et al.(2015) dataset	Presence in the 72 archaeal protein families	Selected for the Slow-Fast analysis
	Acidianus_hospitalis_W1	Crenarchaeota (TACK)		67	X
	Acidiplasma_aeolicum	Diaforarchaea		63	
	Acidiplasma_cupricumulans_JCM_13668	Diaforarchaea			
	Acidiplasma_sp_MBA_1	Diaforarchaea			
	Aciduliprofundum_boonei_T469	Diaforarchaea	X	68	X
	Aciduliprofundum_sp_MAR08_339	Diaforarchaea		68	
	Aeropyrum_camini_SY1___JCM_12091	Crenarchaeota (TACK)		66	
	Aeropyrum_pernix_K1	Crenarchaeota (TACK)			
	Altiarchaeales_archaeon_IMC4	Altiarchaea			
	ANME1	Methanotecta		65	
	Arc_I_group_archaeon_ADurb1013_Bin02101	Acherontia			
	Arc_I_group_archaeon_ADurb1113_Bin01801	Acherontia			
	Arc_I_group_archaeon_ADurb1213_Bin02801	Acherontia		61	
	Arc_I_group_archaeon_B03fssc0709_Meth_Bin005	Acherontia			
	Arc_I_group_archaeon_B15fssc0709_Meth_Bin003	Acherontia		66	X
	Arc_I_group_archaeon_BMIXfssc0709_Meth_Bin006	Acherontia			
	Arc_I_group_archaeon_U1lsi0528_Bin055	Acherontia			
	Arc_I_group_archaeon_U1lsi0528_Bin089	Acherontia		59	
	Archaeoglobus_fulgidus_DSM_4304	Methanotecta	X	70	
	Archaeoglobus_profundus_DSM_5631	Methanotecta	X	72	X
	Archaeoglobus_sulfaticallidus_PM70_1	Methanotecta		69	
	Archaeoglobus_veneficus_SNP6	Methanotecta	X	70	X
	Archaeon_GW2011_AR1	DPANN		72	X
	Archaeon_GW2011_AR10	DPANN		49	X
	Archaeon_GW2011_AR11	DPANN		41	
	Archaeon_GW2011_AR15	DPANN		52	
	Archaeon_GW2011_AR17	DPANN		39	
	Archaeon_GW2011_AR18	DPANN			
	Archaeon_GW2011_AR20	DPANN			
	Archaeon_GW2011_AR3	DPANN		39	
	Archaeon_GW2011_AR4	DPANN		43	X
	Archaeon_GW2011_AR5	DPANN		39	X
	Archaeon_GW2011_AR6	DPANN		19	
	Archaeon_nanoarchaeote_Nst1	DPANN		40	X
	Archaeon_V1_archaea	Verstraetarchaeota			
	Archaeon_V2_archaea	Verstraetarchaeota		68	X
	Archaeon_V4_archaea	Verstraetarchaeota		66	
	Archaeon_V5_archaea	Verstraetarchaeota		41	X
	Bathyarchaeota_archaeon_BA1	Bathyarchaeota (TACK)		59	
	Bathyarchaeota_archaeon_BA2	Bathyarchaeota (TACK)		63	X
	Caldisphaera_lagunensis_DSM_15908	Crenarchaeota (TACK)		61	X
	Caldivirga_maquilingensis_IC_167	Crenarchaeota (TACK)	X	70	X
	Caldivirga_sp_CIS_19	Crenarchaeota (TACK)			
	Caldivirga_sp_JCHS_4	Crenarchaeota (TACK)			
	Caldivirga_sp_MG_3	Crenarchaeota (TACK)		64	
	candidate_division_YNPFFA_archaeon_SCGC_AAA	Crenarchaeota (TACK)			
	candidate_division_YNPFFA_archaeon_SCGC_AAA	Crenarchaeota (TACK)			
	candidate_division_YNPFFA_archaeon_SCGC_AAA	Crenarchaeota (TACK)			
	candidate_divison_MSBL1_archaeon_SCGC_AAA25	Stygia		33	X

candidate_divison_MSBL1_archaeon_SCGC_AAA26	Stygia		23	
Candidatus_Acidianus_copahuensis	Crenarchaeota (TACK)		70	
Candidatus_Aenigmarchaeum_subterraneum_SCGC	DPANN		14	X
Candidatus_Altiarchaeales_archaeon_WOR_SM1_7	Altiarchaea			
Candidatus_Altiarchaeales_archaeon_WOR_SM1_8	Altiarchaea		48	
Candidatus_Altiarchaeales_archaeon_WOR_SM1_S	Altiarchaea		54	
Candidatus_Bathyarchaeota_archaeon_B23	Bathyarchaeota (TACK)		46	
Candidatus_Bathyarchaeota_archaeon_B24	Bathyarchaeota (TACK)		59	X
Candidatus_Bathyarchaeota_archaeon_B25	Bathyarchaeota (TACK)		59	X
Candidatus_Bathyarchaeota_archaeon_B26	Bathyarchaeota (TACK)			
Candidatus_Bathyarchaeota_archaeon_B63	Bathyarchaeota (TACK)		43	
Candidatus_Bathyarchaeota_archaeon_TCS64	Bathyarchaeota (TACK)		40	
Candidatus_Caldiarchaeum_subterraneum	Thaumarchaeota (TACK)	X	55	X
Candidatus_Geoarchaeota	Geoarchaeota (TACK)		57	X
Candidatus_Halobonum_tyrrellensis_G22	DPANN		69	
Candidatus_Haloredivivus_sp_G17	DPANN		59	
Candidatus_Heimdallarchaeota_archaeon_AB_125	Asgard		59	X
Candidatus_Heimdallarchaeota_archaeon_LC_2	Asgard		52	
Candidatus_Heimdallarchaeota_archaeon_LC_3	Asgard		56	
Candidatus_Korarchaeum_cryptofilum_OPF8	Korarchaeota (TACK)	X	56	
Candidatus_Lokiarchaeota_archaeon_CR_4	Asgard		51	
Candidatus_Methanohalarchaeum_thermophilum	Methanonatronarchaeia		68	
Candidatus_Methanomassiliicoccus_intestinalis_Isso	Diaforarchaea		63	
Candidatus_Methanomethylophilus_alvus_Mx1201	Diaforarchaea	X	66	
Candidatus_Methanomethylophilus_sp_1R26	Diaforarchaea			
Candidatus_Methanoperedens_nitroreducens	Methanomicrobia (Methanotecta)		69	
Candidatus_Methanoperedens_sp_BLZ1	Methanomicrobia (Methanotecta)		63	
Candidatus_Methanoplasma_termitum	Diaforarchaea		67	
Candidatus_Nanosalina_sp_J07AB43	DPANN		53	X
Candidatus_Nanosalinarum_sp_J07AB56	DPANN		46	
Candidatus_Nitrosoarchaeum_koreensis_MY1	Thaumarchaeota (TACK)			
Candidatus_Nitrosoarchaeum_limnia_SFB1	Thaumarchaeota (TACK)	X	72	
Candidatus_Nitrosopelagicus_brevis	Thaumarchaeota (TACK)		66	
Candidatus_Nitrosopumilus_koreensis_AR1	Thaumarchaeota (TACK)			
Candidatus_Nitrosopumilus_salaria_BD31	Thaumarchaeota (TACK)			
Candidatus_Nitrosopumilus_sp_AR2	Thaumarchaeota (TACK)		68	
Candidatus_Nitrosopumilus_sp_D3C	Thaumarchaeota (TACK)			
Candidatus_Nitrosopumilus_sp_NF5	Thaumarchaeota (TACK)			
Candidatus_Nitrososphaera_evergladensis_SR1	Thaumarchaeota (TACK)			
Candidatus_Nitrososphaera_gargensis_Ga9	Thaumarchaeota (TACK)	X	72	X
Candidatus_Odinarchaeota_archaeon_LCB_4	Asgard		66	X
Candidatus_Thorarchaeota_archaeon_AB_25	Asgard		65	X
Candidaus_Iainarchaeum_andersonii_SCGC_AAA01	DPANN		12	X
Crenarchaeota_archaeon_SCGC_AAA471_L13	Crenarchaeota (TACK)			
Crenarchaeote_JGI_OTU_1	Crenarchaeota (TACK)			
Crenarchaeote_SCGC_AAA261_C22	Crenarchaeota (TACK)			
Crenarchaeote_SCGC_AAA261_N23	Crenarchaeota (TACK)		43	X
Desulfurococcus_amylolyticus_Z_533	Crenarchaeota (TACK)	X		
Desulfurococcus_fermentans_DSM_16532	Crenarchaeota (TACK)		67	
Desulfurococcus_mobilis_DSM_2161	Crenarchaeota (TACK)			
Desulfurococcus_mucosus_DSM_2162	Crenarchaeota (TACK)		67	X
Euryarchaeota_archaeon_SCGC_AAA252_I15	unclassified Euryarchaeota		46	
Euryarchaeota_archaeon_SG8_5	unclassified Euryarchaeota		43	
Euryarchaeota_archaeon_SM23_78	unclassified Euryarchaeota		53	
Ferroglobus_placidus_DSM_10642	Methanotecta	X	66	X

Ferroplasma_acidarmanus_fer1	Diaforarchaea	X	62	
Ferroplasma_sp_Type_II	Diaforarchaea			
Fervidicoccus_fontis_Kam940	Crenarchaeota (TACK)		57	
Hadesarchaea_archaeon_DG_33	Stygia		48	X
Hadesarchaea_archaeon_DG_33_1	Stygia		56	
Hadesarchaea_archaeon_YNP_N21	Stygia		57	
Haladaptatus_cibarius_D43	Halobacteria (Methanotecta)		65	
Haladaptatus_paucihalophilus_DX253	Halobacteria (Methanotecta)			
Halalkalicoccus_jeotgali_B3	Halobacteria (Methanotecta)			
Halalkalicoccus_paucihalophilus	Halobacteria (Methanotecta)		64	
Halarchaeum_acidiphilum_MH1_52_1	Halobacteria (Methanotecta)		64	
haloarchaeon_3A1_DGR	Halobacteria (Methanotecta)			
Haloarcula_amylolytica_JCM_13557	Halobacteria (Methanotecta)		71	
Haloarcula_argentinensis_DSM_12282	Halobacteria (Methanotecta)			
Haloarcula_californiae_ATCC_33799	Halobacteria (Methanotecta)			
Haloarcula_hispanica_ATCC_33960	Halobacteria (Methanotecta)			
Haloarcula_japonica_DSM_6131	Halobacteria (Methanotecta)			
Haloarcula_sinaiiensis_ATCC_33800	Halobacteria (Methanotecta)			
Haloarcula_sp_CBA1115	Halobacteria (Methanotecta)			
Haloarcula_sp_CBA1127	Halobacteria (Methanotecta)			
Haloarcula_sp_CBA1128	Halobacteria (Methanotecta)			
Haloarcula_sp_SL3	Halobacteria (Methanotecta)			
Haloarcula_vallismortis_ATCC_29715	Halobacteria (Methanotecta)			
Halobacteriaceae_archaeon_SB9	Halobacteria (Methanotecta)			
Halobacterium_salinarum_R1	Halobacteria (Methanotecta)			
Halobacterium_sp_CBA1132	Halobacteria (Methanotecta)			
Halobacterium_sp_DL1	Halobacteria (Methanotecta)			
Halobiforma_lacisalsi_AJ5	Halobacteria (Methanotecta)			
Halobiforma_nitratireducens_JCM_10879	Halobacteria (Methanotecta)			
Halococcus_hamelinensis_100A6	Halobacteria (Methanotecta)			
Halococcus_morrhuae_DSM_1307	Halobacteria (Methanotecta)			
Halococcus_saccharolyticus_DSM_5350	Halobacteria (Methanotecta)			
Halococcus_salifodinae_DSM_8989	Halobacteria (Methanotecta)			
Halococcus_thailandensis_JCM_13552	Halobacteria (Methanotecta)		66	
Haloferax_denitrificans_ATCC_35960	Halobacteria (Methanotecta)			
Haloferax_elongans_ATCC_BAA_1513	Halobacteria (Methanotecta)			
Haloferax_larsenii_JCM_13917	Halobacteria (Methanotecta)			
Haloferax_lucentense_DSM_14919	Halobacteria (Methanotecta)			
Haloferax_mediterranei_ATCC_33500	Halobacteria (Methanotecta)			
Haloferax_mucosum_ATCC_BAA_1512	Halobacteria (Methanotecta)			
Haloferax_prahovense_DSM_18310	Halobacteria (Methanotecta)			
Haloferax_sp_ATCC_BAA_644	Halobacteria (Methanotecta)			
Haloferax_sp_ATCC_BAA_645	Halobacteria (Methanotecta)			
Haloferax_sp_ATCC_BAA_646	Halobacteria (Methanotecta)			
Haloferax_sp_BAB2207	Halobacteria (Methanotecta)			
Haloferax_sp_Q22	Halobacteria (Methanotecta)			
Haloferax_sp_SB29	Halobacteria (Methanotecta)			
Haloferax_sp_SB3	Halobacteria (Methanotecta)			
Haloferax_sulfurifontis_ATCC_BAA_897	Halobacteria (Methanotecta)		68	
Haloferax_volcanii_DS2	Halobacteria (Methanotecta)	X		
Halogeometricum_borinquense_DSM_11551	Halobacteria (Methanotecta)			
Halogeometricum_pallidum_JCM_14848	Halobacteria (Methanotecta)			
Halogranum_salarium_B_1	Halobacteria (Methanotecta)		68	
Halomicrobium_katesii_DSM_19301	Halobacteria (Methanotecta)		66	
Halomicrobium_mukohataei_DSM_12286	Halobacteria (Methanotecta)			

Halonotius_sp_J07HN4	Halobacteria (Methanotecta)			
Halonotius_sp_J07HN6	Halobacteria (Methanotecta)			
Halopenitus_sp_DYS4	Halobacteria (Methanotecta)			
Halophilic_archaeon_DL31	Halobacteria (Methanotecta)			
Halophilic_archaeon_J07HB67	Halobacteria (Methanotecta)			
Halophilic_archaeon_J07HX64	Halobacteria (Methanotecta)			
Halopiger_xanaduensis_SH_6	Halobacteria (Methanotecta)			
Haloplanus_natans_DSM_17983	Halobacteria (Methanotecta)		64	
Haloquadratum_sp_J07HQX50	Halobacteria (Methanotecta)			
Haloquadratum_walsbyi_C23	Halobacteria (Methanotecta)		68	
Halorhabdus_tiamatea_SARL4B	Halobacteria (Methanotecta)			
Halorhabdus_utahensis_DSM_12940	Halobacteria (Methanotecta)			
Halorubrum_aidingense_JCM_13560	Halobacteria (Methanotecta)			
Halorubrum_arcis_JCM_13916	Halobacteria (Methanotecta)			
Halorubrum_californiensis_DSM_19288	Halobacteria (Methanotecta)			
Halorubrum_coriense_DSM_10284	Halobacteria (Methanotecta)			
Halorubrum_distributum_JCM_9100	Halobacteria (Methanotecta)			
Halorubrum_ezzemoulense_DSM_17463	Halobacteria (Methanotecta)			
Halorubrum_halophilum	Halobacteria (Methanotecta)			
Halorubrum_hochstenium_ATCC_700873	Halobacteria (Methanotecta)			
Halorubrum_kocurii_JCM_14978	Halobacteria (Methanotecta)			
Halorubrum_lacusprofundi_ATCC_49239	Halobacteria (Methanotecta)			
Halorubrum_lipolyticum_DSM_21995	Halobacteria (Methanotecta)			
Halorubrum_litoreum_JCM_13561	Halobacteria (Methanotecta)			
Halorubrum_saccharovorum_DSM_1137	Halobacteria (Methanotecta)			
Halorubrum_sp_AJ67	Halobacteria (Methanotecta)			
Halorubrum_sp_BV1	Halobacteria (Methanotecta)			
Halorubrum_sp_J07HR59	Halobacteria (Methanotecta)			
Halorubrum_tebenquichense_DSM_14210	Halobacteria (Methanotecta)		66	
Halorubrum_terrestre_JCM_10247	Halobacteria (Methanotecta)			
Halostagnicola_larsenii_XH_48	Halobacteria (Methanotecta)			
Halostagnicola_sp_A56	Halobacteria (Methanotecta)			
Haloterrigena_jeotgali_A29	Halobacteria (Methanotecta)			
Haloterrigena_limicola_JCM_13563	Halobacteria (Methanotecta)			
Haloterrigena_salina_JCM_13891	Halobacteria (Methanotecta)			
Haloterrigena_thermotolerans_DSM_11522	Halobacteria (Methanotecta)			
Haloterrigena_turkmenica_DSM_5511	Halobacteria (Methanotecta)			
Halovivax_asiaticus_JCM_14624	Halobacteria (Methanotecta)			
Halovivax_ruber_XH_70	Halobacteria (Methanotecta)		63	
Hyperthermus_butylicus_DSM_5456	Crenarchaeota (TACK)		68	X
Ignicoccus_hospitalis_KIN4I	Crenarchaeota (TACK)	X	69	
Ignicoccus_islandicus_DSM_13165	Crenarchaeota (TACK)		67	X
Ignisphaera_aggregans_DSM_17230	Crenarchaeota (TACK)		64	
Lokiarchaeum_sp_GC14_75	Asgard		72	
Marine_Group_I_thaumarchaeote_SCGC_AAA799_E	Thaumarchaeota (TACK)			
Marine_Group_I_thaumarchaeote_SCGC_AAA799_D	Thaumarchaeota (TACK)			
Marine_Group_I_thaumarchaeote_SCGC_AAA799_E	Thaumarchaeota (TACK)			
Marine_Group_I_thaumarchaeote_SCGC_AAA799_N	Thaumarchaeota (TACK)			
Marine_Group_I_thaumarchaeote_SCGC_AAA799_F	Thaumarchaeota (TACK)			
Marine_Group_I_thaumarchaeote_SCGC_AB_629_I2	Thaumarchaeota (TACK)			
Marine_Group_I_thaumarchaeote_SCGC_RSA3	Thaumarchaeota (TACK)			
Metallosphaera_cuprina_Ar_4	Crenarchaeota (TACK)		69	
Metallosphaera_hakonensis_JCM_8857	Crenarchaeota (TACK)			
Metallosphaera_sedula_DSM_5348	Crenarchaeota (TACK)	X	70	
Metallosphaera_yellowstonensis_MK1	Crenarchaeota (TACK)		70	

Archaea

Methanobacterium_formicicum	Methanomada			
Methanobacterium_sp_Maddingley_MBC34	Methanomada		69	
Methanobacterium_sp_MB1	Methanomada			
Methanobacterium_sp_SMA_27	Methanomada		67	
Methanobrevibacter_arboriphilus_ANOR1	Methanomada		54	
Methanobrevibacter_boviskoreani_JH1	Methanomada		68	
Methanobrevibacter_oralis_JMR01	Methanomada		64	
Methanobrevibacter_ruminantium_M1	Methanomada		58	
Methanobrevibacter_smithii_ATCC_35061	Methanomada	X		
Methanobrevibacter_smithii_CAG186	Methanomada		68	
Methanobrevibacter_sp_AbM4	Methanomada			
Methanocaldococcus_fervens_AG86	Methanomada			
Methanocaldococcus_infernus_ME	Methanomada	X	69	
Methanocaldococcus_jannaschii_DSM_2661	Methanomada	X		
Methanocaldococcus_sp_FS406_22	Methanomada			
Methanocaldococcus_villosus_KIN24_T80	Methanomada		67	
Methanocaldococcus_vulcanius_M7	Methanomada			
Methanocella_arvoryzae_MRE50	Methanomicrobia (Methanotecta)	X	67	
Methanocella_conradii_HZ254	Methanomicrobia (Methanotecta)	X		
Methanocella_paludicola_SANAE	Methanomicrobia (Methanotecta)	X	69	X
Methanococcoides_burtonii_DSM_6242	Methanomicrobia (Methanotecta)	X		
Methanococcoides_methylutens_MM1	Methanomicrobia (Methanotecta)		69	
Methanococcus_aeolicus_Nankai_3	Methanomada		67	
Methanococcus_maripaludis_C7	Methanomada			
Methanococcus_vannielii_SB	Methanomada	X	69	
Methanococcus_voltae_A3	Methanomada		66	
Methanoculleus_bourgensis_MS2	Methanomicrobia (Methanotecta)		69	
Methanoculleus_chikugoensis_JCM_10825	Methanomicrobia (Methanotecta)			
Methanoculleus_marisnigri_JR1	Methanomicrobia (Methanotecta)	X	72	X
Methanoculleus_sp_CAG_1088	Methanomicrobia (Methanotecta)			
Methanoculleus_sp_DTU006	Methanomicrobia (Methanotecta)			
Methanoculleus_sp_DTU007	Methanomicrobia (Methanotecta)		68	
Methanoculleus_sp_MH98A	Methanomicrobia (Methanotecta)			
Methanoculleus_sp_S3Fa	Methanomicrobia (Methanotecta)			
Methanoculleus_sp_SDB	Methanomicrobia (Methanotecta)		69	
Methanogenic_archaeon_ISO4_H5	Diaforarchaea		66	
Methanogenic_archaeon_mixed_culture_ISO4_G1	Methanomicrobia (Methanotecta)		62	
Methanogenium_cariaci_JCM_10550	Methanomicrobia (Methanotecta)		52	
Methanohalobium_evestigatum_Z_7303	Methanomicrobia (Methanotecta)		68	
Methanohalophilus_mahii_DSM_5219	Methanomicrobia (Methanotecta)		70	
Methanohalophilus_sp_T328_1	Methanomicrobia (Methanotecta)			
Methanolacinia_petrolearia_DSM_11571	Methanomicrobia (Methanotecta)		69	
Methanolinea_sp_SDB	Methanomicrobia (Methanotecta)		64	
Methanolinea_tarda_NOBI_1	Methanomicrobia (Methanotecta)		69	
Methanolobus_psychrophilus_R15	Methanomicrobia (Methanotecta)		69	
Methanolobus_sp_T82_4	Methanomicrobia (Methanotecta)			
Methanolobus_tindarius_DSM_2278	Methanomicrobia (Methanotecta)		67	
Methanomassiliicoccales_archaeon_RumEn_M1	Diaforarchaea		62	
Methanomassiliicoccales_archaeon_RumEn_M2	Diaforarchaea		66	
Methanomassiliicoccus_luminyensis_B10	Diaforarchaea	X	67	X
Methanomethylovorans_hollandica_DSM_15978	Methanomicrobia (Methanotecta)		67	
Methanomicrobia_archaeon_DTU008	Methanomicrobia (Methanotecta)			
Methanomicrobium_mobile_BP	Methanomicrobia (Methanotecta)		69	
Methanonatronarchaeum_thermophilum	Methanonatronarchaeia		68	
Methanoplanus_limicola_DSM_2279	Methanomicrobia (Methanotecta)		69	

Methanopyrus_kandleri_AV19	Methanomada		53	
Methanoregula_boonei_6A8	Methanomicrobia (Methanotecta)	X	68	
Methanoregula_formicica_SMSP	Methanomicrobia (Methanotecta)		69	
Methanosaeta_concilii_GP6	Methanomicrobia (Methanotecta)		67	
Methanosaeta_harundinacea_6Ac	Methanomicrobia (Methanotecta)	X	69	
Methanosaeta_thermophila_PT	Methanomicrobia (Methanotecta)		69	
Methanosalsum_zhilinae_DSM_4017	Methanomicrobia (Methanotecta)		68	
Methanosarcina_acetivorans_C2A	Methanomicrobia (Methanotecta)			
Methanosarcina_horonobensis_HB_1___JCM_15518	Methanomicrobia (Methanotecta)			
Methanosarcina_lacustris_Z_7289	Methanomicrobia (Methanotecta)			
Methanosarcina_mazei_Go1	Methanomicrobia (Methanotecta)	X	68	
Methanosarcina_siciliae_T4M	Methanomicrobia (Methanotecta)			
Methanosarcina_sp_1_H_A_2_2	Methanomicrobia (Methanotecta)			
Methanosarcina_sp_1_H_T_1A_1	Methanomicrobia (Methanotecta)			
Methanosarcina_sp_2_H_A_1B_4	Methanomicrobia (Methanotecta)			
Methanosarcina_sp_2_H_T_1A_15	Methanomicrobia (Methanotecta)			
Methanosarcina_sp_2_H_T_1A_3	Methanomicrobia (Methanotecta)			
Methanosarcina_sp_795	Methanomicrobia (Methanotecta)			
Methanosarcina_sp_DTU009	Methanomicrobia (Methanotecta)			
Methanosarcina_sp_Kolksee	Methanomicrobia (Methanotecta)			
Methanosarcina_sp_MTP4	Methanomicrobia (Methanotecta)		67	
Methanosarcina_sp_WH1	Methanomicrobia (Methanotecta)			
Methanosarcina_sp_WWM596	Methanomicrobia (Methanotecta)			
Methanosarcina_thermophila_TM_1	Methanomicrobia (Methanotecta)		68	X
Methanosarcina_vacuolata_Z_761	Methanomicrobia (Methanotecta)			
Methanosarcinales_archeaon_56_1174	Methanomicrobia (Methanotecta)		57	
Methanosphaera_stadtmanae_DSM_3091	Methanomada		44	
Methanosphaerula_palustris_E1_9c	Methanomicrobia (Methanotecta)		68	
Methanospirillum_hungatei_JF_1	Methanomicrobia (Methanotecta)		69	X
Methanothermobacter_marburgensis_str_Marburg	Methanomada			
Methanothermobacter_sp_CaT2	Methanomada			
Methanothermobacter_thermautotrophicus_str_Delta	Methanomada	X	70	X
Methanothermococcus_okinawensis_IH1	Methanomada		68	
Methanothermococcus_thermolithotrophicus_DSM_2	Methanomada		71	X
Methanothermus_fervidus_DSM_2088	Methanomada	X	63	X
Methanotorris_formicicus_Mc_S_70	Methanomada		69	X
Methanotorris_igneus_Kol_5	Methanomada	X		
miscellaneous_Crenarchaeota_group_1_archaeon_S	Bathyarchaeota (TACK)		37	
miscellaneous_Crenarchaeota_group_15_archaeon_	Bathyarchaeota (TACK)		63	X
miscellaneous_Crenarchaeota_group_6_archaeon_A	Bathyarchaeota (TACK)		66	
miscellaneous_Crenarchaeota_group_archaeon_SM	Bathyarchaeota (TACK)		41	
miscellaneous_Crenarchaeota_group_archaeon_SM	Bathyarchaeota (TACK)		31	
Nanoarchaeota_archaeon_7A	DPANN			
Nanoarchaeota_archaeon_JGI_OTU_1	DPANN			
Nanoarchaeum_equitans_Kin4_M	DPANN		41	X
Nanohaloarchaea_archaeon_B1_Br10_U2g1	DPANN			
Nanohaloarchaea_archaeon_B1_Br10_U2g19	DPANN			
Nanohaloarchaea_archaeon_B1_Br10_U2g21	DPANN		46	
Nanohaloarchaea_archaeon_B1_Br10_U2g29	DPANN			
Nanohaloarchaea_archaeon_PL_Br10_U2g16	DPANN		59	
Nanohaloarchaea_archaeon_PL_Br10_U2g19	DPANN		56	
Nanohaloarchaea_archaeon_PL_Br10_U2g27	DPANN			
Nanohaloarchaea_archaeon_PL_Br10_U2g5	DPANN			
Natrialba_aegyptia_DSM_13077	Halobacteria (Methanotecta)			
Natrialba_asiatica_DSM_12278	Halobacteria (Methanotecta)			

Natrialba_chahannaoensis_JCM_10990	Halobacteria (Methanotecta)			
Natrialba_hulunbeirensis_JCM_10989	Halobacteria (Methanotecta)			
Natrialba_magadii_ATCC_43099	Halobacteria (Methanotecta)	X		
Natrialba_taiwanensis_DSM_12281	Halobacteria (Methanotecta)			
Natrinema_gari_JCM_14663	Halobacteria (Methanotecta)			
Natrinema_pallidum_DSM_3751	Halobacteria (Methanotecta)		67	
Natrinema_pellirubrum_DSM_15624	Halobacteria (Methanotecta)			
Natrinema_sp_J7	Halobacteria (Methanotecta)			
Natrinema_versiforme_JCM_10478	Halobacteria (Methanotecta)			
Natronobacterium_gregoryi_SP2	Halobacteria (Methanotecta)			
Natronococcus_amylolyticus_DSM_10524	Halobacteria (Methanotecta)			
Natronococcus_jeotgali_DSM_18795	Halobacteria (Methanotecta)			
Natronococcus_occultus_SP4	Halobacteria (Methanotecta)			
Natronolimnobius_baerhuensis_JCM_12253	Halobacteria (Methanotecta)			
Natronolimnobius_innermongolicus_JCM_12255	Halobacteria (Methanotecta)			
Natronomonas_pharaonis_DSM_2160	Halobacteria (Methanotecta)		63	
Natronorubrum_bangense_JCM_10635	Halobacteria (Methanotecta)			
Natronorubrum_sulfidifaciens_JCM_14089	Halobacteria (Methanotecta)			
Natronorubrum_tibetense_GA33	Halobacteria (Methanotecta)			
Nitrosopumilus_maritimus_SCM1	Thaumarchaeota (TACK)	X	69	X
Nitrosopumilus_sp_AR	Thaumarchaeota (TACK)			
Nitrosopumilus_sp_BACL13_MAG_120910_bin56	Thaumarchaeota (TACK)			
Nitrosopumilus_sp_BACL13_MAG_121220_bin23	Thaumarchaeota (TACK)		68	
Nitrosopumilus_sp_SJ	Thaumarchaeota (TACK)			
Nitrososphaera_viennensis_EN76	Thaumarchaeota (TACK)		70	
Palaeococcus_ferrophilus_DSM_13482	Acherontia		69	
Palaeococcus_pacificus_DY20341	Acherontia		71	X
Picrophilus_torridus_DSM_9790	Diaforarchaea		61	
Pyrobaculum_aerophilum_str_IM2	Crenarchaeota (TACK)	X	69	
Pyrobaculum_arsenaticum_DSM_13514	Crenarchaeota (TACK)		69	
Pyrobaculum_calidifontis_JCM_11548	Crenarchaeota (TACK)		68	
Pyrobaculum_islandicum_DSM_4184	Crenarchaeota (TACK)		70	
Pyrobaculum_neutrophilum_V24Sta	Crenarchaeota (TACK)			
Pyrobaculum_oguniense_TE7	Crenarchaeota (TACK)			
Pyrobaculum_sp_WP30	Crenarchaeota (TACK)			
Pyrococcus_abyssi_GE5	Acherontia	X		
Pyrococcus_furiosus_DSM_3638	Acherontia		69	
Pyrococcus_horikoshii_OT3	Acherontia			
Pyrococcus_sp_NA2	Acherontia			
Pyrococcus_sp_ST04	Acherontia			
Pyrococcus_yayanosii_CH1	Acherontia	X		
Salinarchaeum_sp_Harcht_Bsk1	Halobacteria (Methanotecta)		62	
Staphylothermus_hellenicus_DSM_12710	Crenarchaeota (TACK)			
Staphylothermus_marinus_F1	Crenarchaeota (TACK)		66	
Sulfolobales_archaeon_Acd1	Crenarchaeota (TACK)		68	X
Sulfolobales_archaeon_AZ1	Crenarchaeota (TACK)		69	
Sulfolobus_acidocaldarius_DSM_639	Crenarchaeota (TACK)		68	
Sulfolobus_islandicus_L_S_2_15	Crenarchaeota (TACK)		68	
Sulfolobus_metallicus_DSM_6482___JCM_9184	Crenarchaeota (TACK)		57	
Sulfolobus_solfataricus_P2	Crenarchaeota (TACK)			
Sulfolobus_sp_JCM_16833	Crenarchaeota (TACK)			
Thaumarchaeota_archaeon_casp_thauma4	Thaumarchaeota (TACK)			
Thaumarchaeota_archaeon_CSP1_1	Thaumarchaeota (TACK)		72	X
Thaumarchaeota_archaeon_JGI_OTU_1	Thaumarchaeota (TACK)		45	X
Thaumarchaeota_archaeon_JGI_OTU_3	Thaumarchaeota (TACK)		37	X

Thaumarchaeota_archaeon_MY2	Thaumarchaeota (TACK)		72	
Thaumarchaeota_archaeon_MY3	Thaumarchaeota (TACK)			
Thaumarchaeota_archaeon_N4	Thaumarchaeota (TACK)		72	X
Thaumarchaeota_archaeon_SAT1	Thaumarchaeota (TACK)			
Thaumarchaeota_archaeon_SCGC_AAA007_O23	Thaumarchaeota (TACK)		68	
Thaumarchaeota_archaeon_SCGC_AAA282_K18	Thaumarchaeota (TACK)		56	
Thaumarchaeota_archaeon_SCGC_AAA287_E17	Thaumarchaeota (TACK)			
Thermocladium_modestius_JCM_10088	Crenarchaeota (TACK)			
Thermocladium_sp_ECH_B	Crenarchaeota (TACK)		66	
Thermococcus_barophilus_MP	Acherontia	X	70	X
Thermococcus_celer_JCM_8558	Acherontia			
Thermococcus_gammatolerans_EJ3	Acherontia		71	
Thermococcus_kodakarensis_KOD1	Acherontia			
Thermococcus_litoralis_DSM_5473	Acherontia	X	68	
Thermococcus_onnurineus_NA1	Acherontia		70	
Thermococcus_peptonophilus_JCM_9653	Acherontia			
Thermococcus_sibiricus_MM_739	Acherontia			
Thermococcus_sp_AM4	Acherontia			
Thermococcus_sp_EP1	Acherontia			
Thermococcus_sp_JCM_11816	Acherontia			
Thermococcus_sp_PK	Acherontia			
Thermococcus_zilligii_AN1	Acherontia			
Thermofilum_pendens_Hrk_5	Crenarchaeota (TACK)	X	56	X
Thermogymnomonas_acidicola_JCM_13583	Diaforarchaea		44	
Thermoplasma_acidophilum_DSM_1728	Diaforarchaea		60	
Thermoplasma_volcanium_GSS1	Diaforarchaea		60	X
Thermoplasmatales_archaeon_A_plasma	Diaforarchaea		59	
Thermoplasmatales_archaeon_BRNA1	Diaforarchaea		62	
Thermoplasmatales_archaeon_DG_70	Diaforarchaea		43	
Thermoplasmatales_archaeon_DG_70_1	Diaforarchaea		50	
Thermoplasmatales_archaeon_E_plasma	Diaforarchaea		62	
Thermoplasmatales_archaeon_SCGC_AB_539_N05	Diaforarchaea		41	
Thermoplasmatales_archaeon_SG8_52	Diaforarchaea		72	X
Thermoplasmatales_archaeon_SM1_50	Diaforarchaea		62	
Thermoproteus_sp_AZ2	Crenarchaeota (TACK)		70	
Thermoproteus_sp_CIS_19	Crenarchaeota (TACK)			
Thermoproteus_sp_JCHS_4	Crenarchaeota (TACK)		64	
Thermoproteus_tenax_Kra_1	Crenarchaeota (TACK)	X	70	X
Thermosphaera_aggregans_DSM_11486	Crenarchaeota (TACK)		67	X
Thorarchaeota_archaeon_SMTZ_45	Asgard			
Thorarchaeota_archaeon_SMTZ1_45	Asgard		58	
Thorarchaeota_archaeon_SMTZ1_83	Asgard		70	
uncultured_Acidilobus_sp_MG	Crenarchaeota (TACK)		64	
Uncultured_archaeon_A07HB70	uncultured Archaea			
Uncultured_archaeon_A07HN63	uncultured Archaea			
Uncultured_archaeon_A07HR60	uncultured Archaea			
Uncultured_archaeon_A07HR67	uncultured Archaea			
Uncultured_marine_group_II_euryarchaeote	Diaforarchaea	X	42	
Vulcanisaeta_distributa_DSM_14429	Crenarchaeota (TACK)		70	X
Vulcanisaeta_souniana_JCM_11219	Crenarchaeota (TACK)		52	
Vulcanisaeta_sp_AZ3	Crenarchaeota (TACK)		56	
Vulcanisaeta_sp_CIS_19	Crenarchaeota (TACK)			
Vulcanisaeta_sp_JCHS_4	Crenarchaeota (TACK)		58	
Vulcanisaeta_sp_JCM_14467	Crenarchaeota (TACK)			
Vulcanisaeta_sp_JCM_16159	Crenarchaeota (TACK)			

	Vulcanisaeta_sp_JCM_16161	Crenarchaeota (TACK)			
**Bacteria**	Acetobacter_pasteurianus_IFO_3283_01_42C	Alphaproteobacteria	X		
	Acidaminococcus_fermentans_DSM_20731	Firmicutes	X		
	Acinetobacter_baumannii_1656_2	Gammaproteobacteria	X		
	Actinosynnema_mirum_DSM_43827	Actinobacteria	X		
	Akkermansia_muciniphila_ATCC_BAA_835	Verrucomicrobia	X		
	Allochromatium_vinosum_DSM_180	Gammaproteobacteria	X		
	Alteromonas_macleodii_str_Black_Sea_11	Gammaproteobacteria	X		
	Anabaena_variabilis_ATCC_29413	Cyanobacteria	X		
	Anaerolinea_thermophila_UNI_1	Chloroflexi	X		
	Arcobacter_butzleri_ED_1	Epsilonproteobacteria	X		
	Bacteroides_fragilis_638R	Bacteroidetes_Chlorobi	X		
	Bartonella_bacilliformis_KC583	Alphaproteobacteria	X		
	Borrelia_afzelii_PKo	Spirochaetes	X		
	Brachyspira_hyodysenteriae_WA1	Spirochaetes	X		
	Burkholderia_ambifaria_AMMD	Betaproteobacteria	X		
	Caldilinea_aerophila_DSM_14535_NBRC_104270	Chloroflexi	X		
	Candidatus_Protochlamydia_amoebophila_UWE25	Chlamydiae	X		
	Catenulispora_acidiphila_DSM_44928	Actinobacteria	X		
	Chlamydia_muridarum_Nigg	Chlamydiae	X		
	Chlorobaculum_parvum_NCIB_8327	Bacteroidetes_Chlorobi	X		
	Chromobacterium_violaceum_ATCC_12472	Betaproteobacteria	X		
	Coraliomargarita_akajimensis_DSM_45221	Verrucomicrobia	X		
	Cyanothece_sp_PCC_7424	Cyanobacteria	X		
	Cytophaga_hutchinsonii_ATCC_33406	Bacteroidetes_Chlorobi	X		
	Dehalococcoides_mccartyi	Chloroflexi	X		
	Deinococcus_deserti_VCD115	Deinococci-Thermus	X		
	Desulfobulbus_propionicus_DSM_2032	Deltaproteobacteria	X		
	Dinoroseobacter_shibae_DFL_12	Alphaproteobacteria	X		
	Fervidobacterium_nodosum_Rt17_B1	Thermotogae	X		
	Gloeobacter_violaceus_PCC_7421	Cyanobacteria	X		
	Helicobacter_acinonychis_str_Sheeba	Epsilonproteobacteria	X		
	Heliobacterium_modesticaldum_Ice1	Firmicutes	X		
	Herpetosiphon_aurantiacus_DSM_785	Chloroflexi	X		
	Isosphaera_pallida_ATCC_43644	Planctomycetes	X		
	Kineococcus_radiotolerans_SRS30216	Actinobacteria	X		
	Kosmotoga_olearia_TBF_19_5_1	Thermotogae	X		
	Lactococcus_garvieae_ATCC_49156	Firmicutes	X		
	Legionella_longbeachae_NSW150	Gammaproteobacteria	X		
	Leptospira_biflexa_serovar_Patoc	Spirochaetes	X		
	Listeria_innocua_Clip11262	Firmicutes	X		
	Marinithermus_hydrothermalis_DSM_14884	Deinococci-Thermus	X		
	Methylacidiphilum_infernorum_V4	Verrucomicrobia	X		
	Natranaerobius_thermophilus_JW_NM_WN_LF	Firmicutes	X		
	Nautilia_profundicola_AmH	Epsilonproteobacteria	X		
	Nitrosomonas_europaea_ATCC_19718	Betaproteobacteria	X		
	Nocardia_cyriacigeorgica_GUH_2	Actinobacteria	X		
	Novosphingobium_aromaticivorans_DSM_12444	Alphaproteobacteria	X		
	Opitutus_terrae_PB90_1	Verrucomicrobia	X		
	Pedobacter_heparinus_DSM_2366	Bacteroidetes_Chlorobi	X		
	Pelobacter_carbinolicus_DSM_2380	Deltaproteobacteria	X		
	Petrotoga_mobilis_SJ95	Thermotogae	X		
	Phycisphaera_mikurensis_NBRC_102666	Planctomycetes	X		
	Planctomyces_brasiliensis_DSM_5305	Planctomycetes	X		
	Rhodopirellula_baltica_SH_1	Planctomycetes	X		

	Rhodothermus_marinus_DSM_4252	Bacteroidetes_Chlorobi	X		
	Simkania_negevensis_Z	Chlamydiae	X		
	Stigmatella_aurantiaca_DW4_3_1	Deltaproteobacteria	X		
	Streptosporangium_roseum_DSM_43021	Actinobacteria	X		
	Synechococcus_elongatus_PCC_6301	Cyanobacteria	X		
	Syntrophobacter_fumaroxidans_MPOB	Deltaproteobacteria	X		
	Thermomicrobium_roseum_DSM_5159	Chloroflexi	X		
	Thermotoga_maritima_MSB8	Thermotogae	X		
	Thiobacillus_denitrificans_ATCC_25259	Betaproteobacteria	X		
	Treponema_azotonutricium_ZAS_9	Spirochaetes	X		
	Trichodesmium_erythraeum_IMS101	Cyanobacteria	X		
	Truepera_radiovictrix_DSM_17093	Deinococci-Thermus	X		
	Waddlia_chondrophila_WSU_86_1044	Chlamydiae	X		
**Eucarya**	Arabidopsis_thaliana		X		
	Aureococcus_anophagefferens		X		
	Batrachochytrium_dendrobatidis		X		
	Chlamydomonas_reinhardtii		X		
	Dictyostelium_discoideum		X		
	Homo_sapiens		X		
	Leishmania_major_strain_Friedlin		X		
	Micromonas_pusilla		X		
	Monosiga_brevicollis		X		
	Naegleria_gruberi		X		
	Paramecium_tetraurelia		X		
	Phaeodactylum_tricornutum		X		
	Physcomitrella_patens		X		
	Saccharomyces_cerevisiae_S288c		X		
	Selaginella_moellendorffii		X		
	Tetrahymena_thermophila		X		
	Thalassiosira_pseudonana		X		
	Trypanosoma_brucei		X		

Supplementary Table S2: List of markers from Raymann et al. (2015) used in this study. For each marker, the corresponding COG name and general description are also indicated. The nine markers displaying complex evolutionary histories are indicated in red. These markers were not retained for supermatrices construction.

Protein families shared by Archaea and eukaryotes

FAM000125	COG1718	Serine/threonine protein kinase involved in cell cycle control	Signal transduction mechanisms, Cell cycle control; cell division; chromosome partitioning	CELLULAR PROCESSES AND SIGNALING
FAM000138	COG1632	Ribosomal protein L15E	Translation; ribosomal structure and biogenesis	INFORMATION STORAGE AND PROCESSING
FAM000158	COG1889	Fibrillarin-like rRNA methylase	Translation; ribosomal structure and biogenesis	INFORMATION STORAGE AND PROCESSING
FAM000164	COG1269	Archaeal/vacuolar-type H+-ATPase subunit I	Energy production and conversion	METABOLISM
FAM000447	COG0051	Ribosomal protein S10	Translation; ribosomal structure and biogenesis	INFORMATION STORAGE AND PROCESSING
FAM000500	COG1890	Ribosomal protein S3AE	Translation; ribosomal structure and biogenesis	INFORMATION STORAGE AND PROCESSING
FAM000504	COG1500	Predicted exosome subunit	Translation; ribosomal structure and biogenesis	INFORMATION STORAGE AND PROCESSING
FAM000527	COG1471	Ribosomal protein S4E	Translation; ribosomal structure and biogenesis	INFORMATION STORAGE AND PROCESSING
FAM000533	COG2147	Ribosomal protein L19E	Translation; ribosomal structure and biogenesis	INFORMATION STORAGE AND PROCESSING
FAM000740	COG0197	Ribosomal protein L16/L10E	Translation; ribosomal structure and biogenesis	INFORMATION STORAGE AND PROCESSING
FAM000754	COG1093	Translation initiation factor 2, alpha subunit (eIF-2alpha)	Translation; ribosomal structure and biogenesis	INFORMATION STORAGE AND PROCESSING
FAM000768	COG1976	Translation initiation factor 6 (eIF-6)	Translation; ribosomal structure and biogenesis	INFORMATION STORAGE AND PROCESSING
FAM000828	COG1867	N2,N2-dimethylguanosine tRNA methyltransferase	Translation; ribosomal structure and biogenesis	INFORMATION STORAGE AND PROCESSING
FAM000834	COG2101	TATA-box binding protein (TBP), component of TFIID and TFIIIB	Transcription	INFORMATION STORAGE AND PROCESSING
FAM002097	COG0638	20S proteasome, beta subunit	Posttranslational modification; protein turnover; chaperones	CELLULAR PROCESSES AND SIGNALING
FAM005140	COG1155	Archaeal/vacuolar-type H+-ATPase subunit A	Energy production and conversion	METABOLISM
RFC_L	COG0470/1	ATPase involved in DNA replication	General function prediction only eplication; recombination and repair	INFORMATION STORAGE AND PROCESSING
FAM000509	COG0423	Glycyl-tRNA synthetase (class II)	Translation; ribosomal structure and biogenesis	INFORMATION STORAGE AND PROCESSING
FAM000524	COG0186	Ribosomal protein S17	Translation; ribosomal structure and biogenesis	INFORMATION STORAGE AND PROCESSING
FAM000531	COG0097	Ribosomal protein L6P/L9E	Translation; ribosomal structure and biogenesis	INFORMATION STORAGE AND PROCESSING
FAM000520	COG0091	Ribosomal protein L22	Translation; ribosomal structure and biogenesis	INFORMATION STORAGE AND PROCESSING
FAM000340	COG1358	Ribosomal protein HS6-type (S12/L30/L7a)	Translation; ribosomal structure and biogenesis	INFORMATION STORAGE AND PROCESSING
FAM000299	COG0456	Acetyltransferases	General function prediction only	POORLY CHARACTERIZED
FAM000395	COG0522/3	Ribosomal protein S4 and related proteins	Translation; ribosomal structure and biogenesis	INFORMATION STORAGE AND PROCESSING
FAM000431	COG1095	DNA-directed RNA polymerase, subunit E'	Transcription	INFORMATION STORAGE AND PROCESSING
FAM000532	COG1717	Ribosomal protein L32E	Translation; ribosomal structure and biogenesis	INFORMATION STORAGE AND PROCESSING
FAM000702	COG1394	Archaeal/vacuolar-type H+-ATPase subunit D	Energy production and conversion	METABOLISM
FAM000752	COG1631	Ribosomal protein L44E	Translation; ribosomal structure and biogenesis	INFORMATION STORAGE AND PROCESSING
FAM000825	COG1498	Protein implicated in ribosomal biogenesis, Nop56p homolog	Translation; ribosomal structure and biogenesis	INFORMATION STORAGE AND PROCESSING
FAM001196	COG0585	Uncharacterized conserved protein	Nucleotide transport and metabolismunction unknown	POORLY CHARACTERIZED
PCNA	COG0592	DNA polymerase sliding clamp subunit (PCNA homolog)	General function prediction only eplication; recombination and repair	INFORMATION STORAGE AND PROCESSING
FAM000496	COG2139	Ribosomal protein L21E	Translation; ribosomal structure and biogenesis	INFORMATION STORAGE AND PROCESSING
FAM001881	COG1537	Predicted RNA-binding proteins	General function prediction only	POORLY CHARACTERIZED
FEN_1	COG0258	5'-3' exonuclease (including N-terminal domain of PolI)	General function prediction only eplication; recombination and repair	INFORMATION STORAGE AND PROCESSING

Protein families shared by Archaea, Bacteria and eukaryotes

FAM000121	COG0017	Aspartyl/asparaginyl-tRNA synthetases	Translation; ribosomal structure and biogenesis	INFORMATION STORAGE AND PROCESSING
FAM000251	COG5256	Translation elongation factor EF-1alpha (GTPase)	Translation; ribosomal structure and biogenesis	INFORMATION STORAGE AND PROCESSING
FAM000308	COG0541	Signal recognition particle GTPase	Intracellular trafficking; secretion; and vesicular transport	CELLULAR PROCESSES AND SIGNALING
FAM000364	COG0459	Chaperonin GroEL (HSP60 family)	Posttranslational modification; protein turnover; chaperones	CELLULAR PROCESSES AND SIGNALING
FAM000388	COG0052	Ribosomal protein S2	Translation; ribosomal structure and biogenesis	INFORMATION STORAGE AND PROCESSING
FAM000390	COG0103	Ribosomal protein S9	Translation; ribosomal structure and biogenesis	INFORMATION STORAGE AND PROCESSING
FAM000391	COG0102	Ribosomal protein L13	Translation; ribosomal structure and biogenesis	INFORMATION STORAGE AND PROCESSING
FAM000448	COG0049	Ribosomal protein S7	Translation; ribosomal structure and biogenesis	INFORMATION STORAGE AND PROCESSING
FAM000452	COG0086	DNA-directed RNA polymerase, beta' subunit/160 kD subunit	Transcription	INFORMATION STORAGE AND PROCESSING
FAM000453	COG0085	DNA-directed RNA polymerase, beta subunit/140 kD subunit	Transcription	INFORMATION STORAGE AND PROCESSING
FAM000518	COG0090	Ribosomal protein L2	Translation; ribosomal structure and biogenesis	INFORMATION STORAGE AND PROCESSING
FAM000521	COG0092	Ribosomal protein S3	Translation; ribosomal structure and biogenesis	INFORMATION STORAGE AND PROCESSING

FAM000525	COG0093	Ribosomal protein L14	Translation; ribosomal structure and biogenesis	INFORMATION STORAGE AND PROCESSING
FAM000528	COG0094	Ribosomal protein L5	Translation; ribosomal structure and biogenesis	INFORMATION STORAGE AND PROCESSING
FAM000535	COG0098	Ribosomal protein S5	Translation; ribosomal structure and biogenesis	INFORMATION STORAGE AND PROCESSING
FAM000537	COG0201	Preprotein translocase subunit SecY	Intracellular trafficking; secretion; and vesicular transport	CELLULAR PROCESSES AND SIGNALING
FAM000773	COG0080	Ribosomal protein L11	Translation; ribosomal structure and biogenesis	INFORMATION STORAGE AND PROCESSING
FAM000774	COG0081	Ribosomal protein L1	Translation; ribosomal structure and biogenesis	INFORMATION STORAGE AND PROCESSING
FAM001256	COG0480	Translation elongation factors (GTPases)	Translation; ribosomal structure and biogenesis	INFORMATION STORAGE AND PROCESSING
FAM002715	COG0533	Metal-dependent proteases with possible chaperone activity	Posttranslational modification; protein turnover; chaperones	CELLULAR PROCESSES AND SIGNALING
FAM003194	COG0532	Translation initiation factor 2 (IF-2; GTPase)	Translation; ribosomal structure and biogenesis	INFORMATION STORAGE AND PROCESSING
FAM005139	COG1156	Archaeal/vacuolar-type H+-ATPase subunit B	Energy production and conversion	METABOLISM
FAM008311	COG0470	ATPase involved in DNA replication	General function prediction only eplication; recombination and repair	INFORMATION STORAGE AND PROCESSING
FAM000450	COG0048	Ribosomal protein S12	Translation; ribosomal structure and biogenesis	INFORMATION STORAGE AND PROCESSING
FAM000394	COG0100	Ribosomal protein S11	Translation; ribosomal structure and biogenesis	INFORMATION STORAGE AND PROCESSING
Sig_reg_part	COG0552	Signal recognition particle GTPase	Intracellular trafficking; secretion; and vesicular transport	CELLULAR PROCESSES AND SIGNALING
FAM000396	COG0099	Ribosomal protein S13	Translation; ribosomal structure and biogenesis	INFORMATION STORAGE AND PROCESSING
FAM000515	COG0087	Ribosomal protein L3	Translation; ribosomal structure and biogenesis	INFORMATION STORAGE AND PROCESSING
FAM000516	COG0088	Ribosomal protein L4	Translation; ribosomal structure and biogenesis	INFORMATION STORAGE AND PROCESSING
FAM000530	COG0096	Ribosomal protein S8	Translation; ribosomal structure and biogenesis	INFORMATION STORAGE AND PROCESSING
FAM003192	COG5257	Translation initiation factor 2, gamma subunit (eIF-2gamma; GTPase)	Translation; ribosomal structure and biogenesis	INFORMATION STORAGE AND PROCESSING
FAM000058	COG0030	Dimethyladenosine transferase (rRNA methylation)	Translation; ribosomal structure and biogenesis	INFORMATION STORAGE AND PROCESSING
FAM000392	COG0200	Ribosomal protein L15	Translation; ribosomal structure and biogenesis	INFORMATION STORAGE AND PROCESSING
FAM000445	COG0130/1	Pseudouridine synthase	Translation; ribosomal structure and biogenesis	INFORMATION STORAGE AND PROCESSING
FAM001381	COG1222	ATP-dependent 26S proteasome regulatory subunit	Posttranslational modification; protein turnover; chaperones	CELLULAR PROCESSES AND SIGNALING
FAM000551	COG0020	Undecaprenyl pyrophosphate synthase	General function prediction only eplication; recombination and repair ipid transport and metabolism	METABOLISM
FAM001151	COG0062/1	Uncharacterized conserved protein	Nucleotide transport and metabolismunction unknown	POORLY CHARACTERIZED
**Protein families shared by Archaea and Bacteria**				
2_FAM000028	COG0528	Uridylate kinase	Nucleotide transport and metabolism	METABOLISM
DnaG	COG0358/3	DNA primase (bacterial type)	General function prediction only eplication; recombination and repair	INFORMATION STORAGE AND PROCESSING
FAM000023	COG0343/1	Queuine/archaeosine tRNA-ribosyltransferase	Translation; ribosomal structure and biogenesis	INFORMATION STORAGE AND PROCESSING
FAM000366	COG0462	Phosphoribosylpyrophosphate synthetase	Nucleotide transport and metabolism Amino acid transport and metabolism	METABOLISM
FAM000371	COG0195	Transcription elongation factor	Transcription	INFORMATION STORAGE AND PROCESSING
FAM000737	COG0012	Predicted GTPase, probable translation factor	Translation; ribosomal structure and biogenesis	INFORMATION STORAGE AND PROCESSING
FAM000772	COG0250	Transcription antiterminator	Transcription	INFORMATION STORAGE AND PROCESSING
FAM000782	COG0126	3-phosphoglycerate kinase	Energy production and conversionarbohydrate transport and metabolism	METABOLISM
FAM003264	COG0149	Triosephosphate isomerase	Energy production and conversionarbohydrate transport and metabolism	METABOLISM
